# LiTMP Trans‐Metal‐Trapping of Fluorinated Aromatic Molecules: A Comparative Study of Aluminum and Gallium Carbanion Traps

**DOI:** 10.1002/anie.201706064

**Published:** 2017-07-17

**Authors:** Ross McLellan, Marina Uzelac, Alan R. Kennedy, Eva Hevia, Robert E. Mulvey

**Affiliations:** ^1^ WestCHEM Department of Pure and Applied Chemistry University of Strathclyde Glasgow G1 1XL UK

**Keywords:** aluminum, carbanions, fluoroaromatic compounds, gallium, metalation

## Abstract

Fluoroaromatic scaffolds pose a challenge to lithiation due to low stability of lithiated intermediates. Here we apply trans‐metal‐trapping (TMT) to a series of key fluorinated aromatics. In TMT, LiTMP performs the metalation, while an organometallic trap intercepts the emergent carbanion. This study contrasts the trapping abilities of *i*Bu_2_AlTMP and Ga(CH_2_SiMe_3_)_3_, structurally mapping their TMT reactions and probing relative stabilities of metalated fluoroaromatic intermediates by NMR studies. Results show the installed Al−C(aryl) bonds are more prone to decomposition by benzyne formation and Li‐F liberation, than the Ga−C(aryl) species. The latter are thus better for onward reactivity as demonstrated in cross‐coupling reactions with benzoyl chloride that produce ketones.

In 2017, we witness the centenary of the advent of organolithium chemistry by Schlenk and Holtz.^1^ Since then organolithium reagents have played leading roles in the synthesis of organic compounds especially through metalation (C−H to C−metal) applications.^2^ Fluorinated aromatic compounds represent a special challenge to organolithium reagents on account of the instability of metalated intermediates.^3^ This point and the profound complexity involved (e.g., benzyne formation, autometalation, cascade processes) are exemplified in Schlosser's classic report of multiple hydrogen/lithium interconversions induced by lithiation of 1,3,5‐trifluorobenzene.^4^ Significantly, fluoroaromatic compounds are rarely found in nature,^5^ meaning that nearly all aryl fluorides utilized in pharmaceutical manufacture (as synthetic building blocks) must be generated synthetically. Incentivized by the growing importance of fluorinated aromatic compounds in active pharmaceutical ingredients (where it is estimated that 20–25 % of forthcoming drugs contain at least one F atom),^6^, ^7^ we pondered whether emerging metalation methodologies could improve on the performances of the classical organolithium reagents. Notable advances to this end have been made with respect to either reaction rate or regioselectivity. Collum and co‐workers demonstrated a rate enhancement on lithiating various fluorinated aromatics using lithium diisopropylamide in THF at −78 °C by adding catalytic quantities of LiCl.^8^ Knochel and co‐workers disclosed that specific aryllithium species can be selectively trapped from a mixture of isomers by transmetalation with a substoichiometric quantity of dichlorozirconocene.^9^ Here we approach these challenging metalations through trans‐metal‐trapping (TMT), where two non‐interacting organometallic reagents work in tandem (Scheme 1).^10^ The first stage of TMT harnesses the bulky amide base, LiTMP (TMP=2,2,6,6‐tetramethylpiperidide) to deprotonate a substrate (these metalations can exist in equilibria lying towards starting materials). The second stage utilizes a bulky, soluble Lewis acidic organometallic trap to rapidly intercept and stabilise emergent carbanions, thereby driving equilibria toward metalated products. Still in its infancy, TMT has only been reported with a handful of organic/organometallic substrates using *i*Bu_2_AlTMP as the trap11a,11b and with a series of diazines using a Ga(CH_2_SiMe_3_)_3_ trap.11c We also note that Knochel's Group used a similar Al reagent, *i*Bu_2_AlCl, to trap aromatic carbanions after lithium halogen exchange, though this proceeded with LiX elimination and thus gave neutral Al species as opposed to the ate species discussed here.^12^ Here, in applying TMT to challenging fluorinated aromatic substrates we present the first comparative study between Al and Ga traps, structurally mapping TMT reactions both crystallographically and spectroscopically, elucidating the complex reaction pathways that diminish the effectiveness of the Al trap, and establishing that the greater carbophilicity and, or the reduced fluorophilicity of the Ga trap makes its products the preferred candidates for performing follow on reactions with electrophiles.

**Scheme 1:**
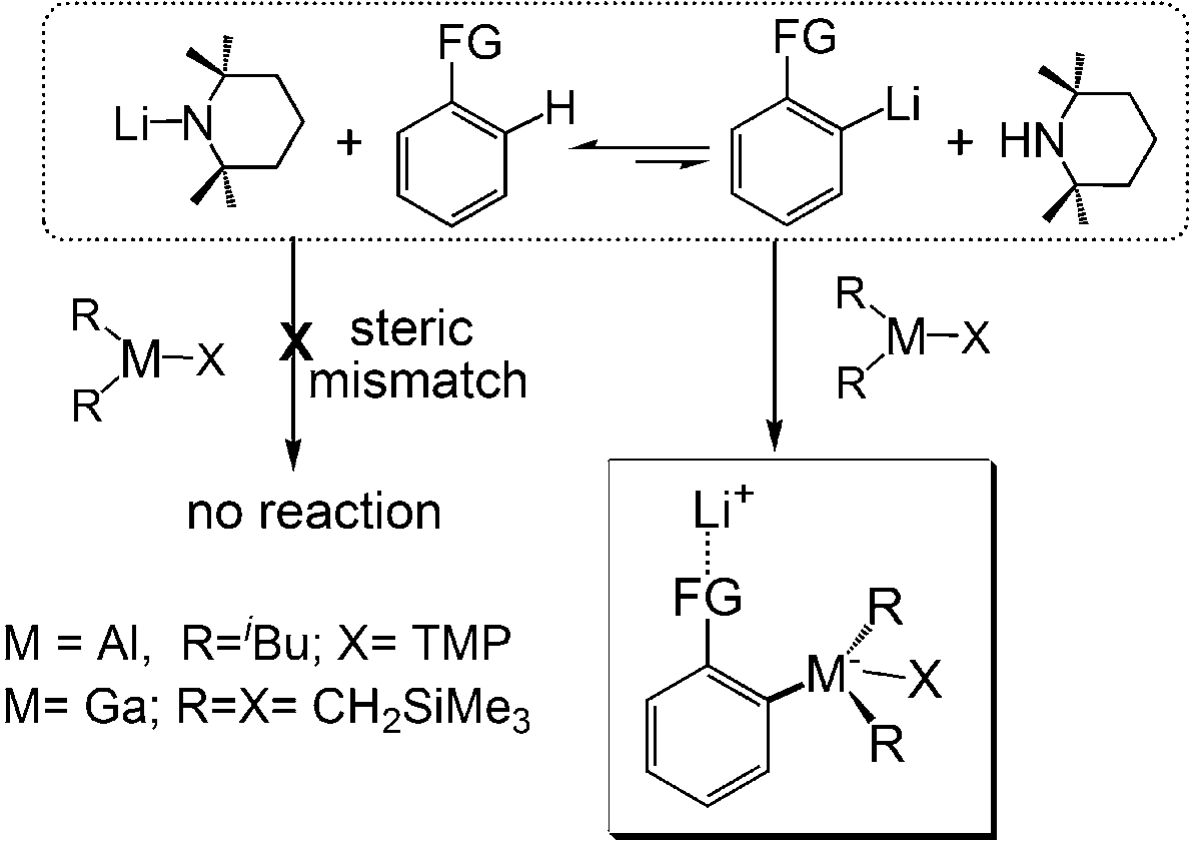
Generic concept of trans‐metal‐trapping (TMT).

Initial TMT studies focused on the LiTMP/*i*Bu_2_AlTMP system with a range of fluorinated aromatic substrates. Reaction of 3‐F‐anisole with LiTMP and *i*Bu_2_AlTMP in hexane at −78 °C gave a solid that ^1^H NMR analysis confirms contains metalated substrate (in the 2‐position) as indicated by three new resonances between 6.94 and 6.19 ppm. ^19^F and ^7^Li NMR spectra support formation of one product, displaying one resonance in each case. X‐ray crystallography revealed this product to be the contacted ion pair **1** [2‐{(*i*Bu)_2_Al(μ‐TMP)Li⋅THF}‐3‐fluoroanisyl] (Figure 1 A), confirming regioselective *ortho*‐metalation of 3‐F‐anisole. Al bonds to the 2‐position of the substrate (C1−Al1 2.0872(17) Å).

**Figure 1:**
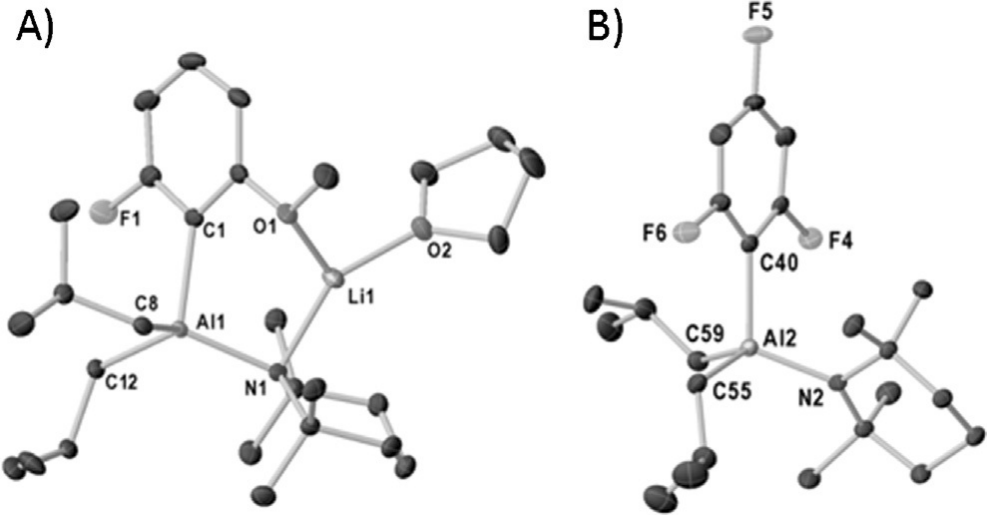
A) Molecular structure of **1**. B) Structure of aluminate anion in **3**. Hydrogen atoms are omitted for clarity and thermal ellipsoids at 30 % probability.

Solvated by the MeO oxygen atom and a THF molecule, Li further bonds to a μ‐TMP ligand. Two *i*Bu groups complete the distorted tetrahedral Al coordination. Demonstrating generality, substrate scope was extended to fluoroarenes containing 2–4 fluorine atoms (Scheme 2): 3,5‐difluoroanisole (giving [4‐{(*i*Bu)_2_(TMP)Al}‐3,5‐difluoroanisyl][Li(THF)_4_], **2**), 1,3,5‐trifluorobenzene (giving [2‐{(*i*Bu)_2_(TMP)Al}‐1,3,5‐F_3_‐C_6_H_2_][Li(THF)_4_], **3**), and 1,2,4,5‐tetrafluorobenzene (giving [3‐{(*i*Bu)_2_(TMP)Al}‐1,2,4,5‐F_4_‐C_6_H_1_][Li(THF)_4_], **4**). Due to the lack of a suitably positioned Lewis basic group on the substrate, all three adopt solvent‐separated ion pair (SSIP) structures (Figure 1 B shows the anion of **3**) with a Li(THF)_4_ countercation (see the Supporting Information for details). Note, the C(aryl)–Al distances reveal an increasing trend with additional F substituents from **1**–**4** (**1** 2.0872(17) Å; **2** 2.076(4) Å; **3** 2.090(3) Å; **4** 2.106(4) Å). Thus it is anticipated that as the F content increases the carbanionic charge decreases, and in theory the trapping step should become less facile. Unfortunately full characterization of **2**–**4** was hampered by poor yields and propensity of crystals to decompose into oils.

**Scheme 2:**
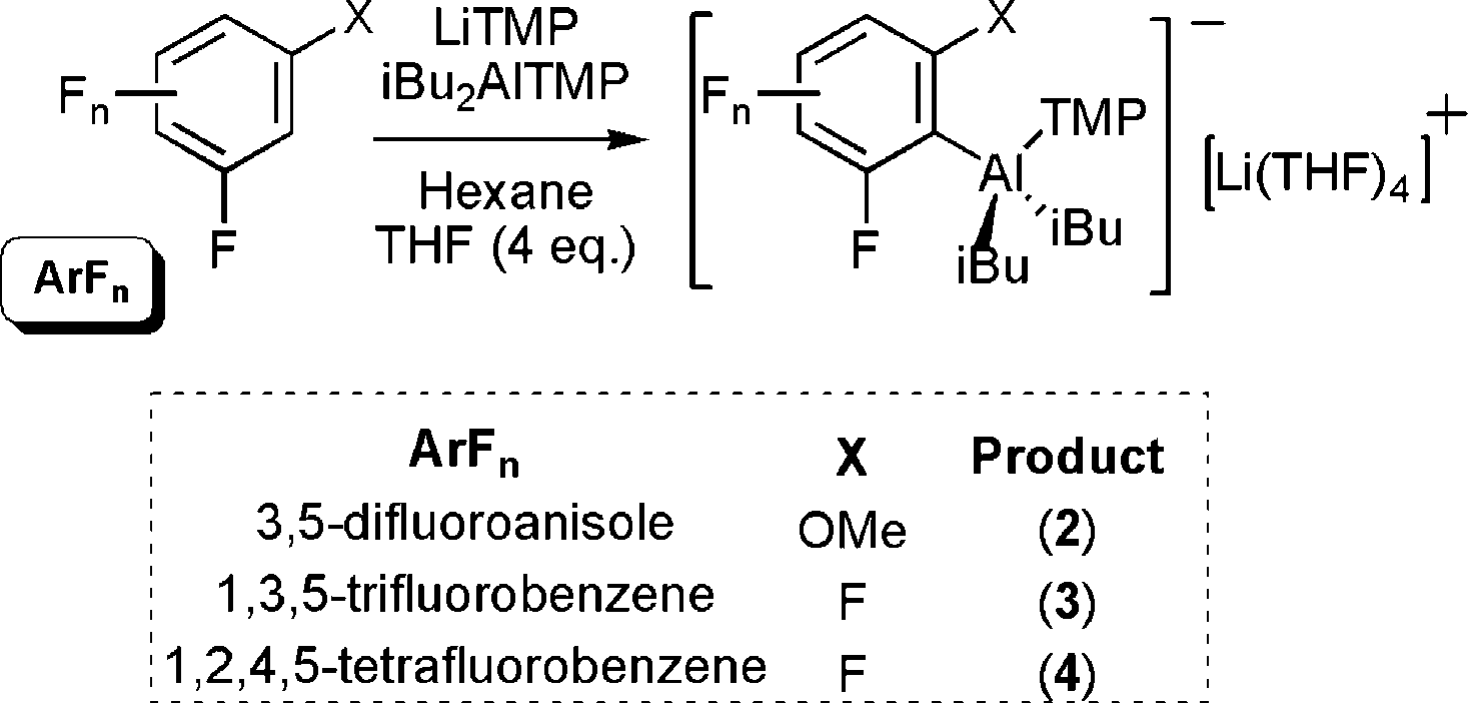
Synthesis of **2**–**4**.

The moderate yields of **1**–**4** prompted more investigation. Using **1** as a representative example, the ^1^H NMR spectrum of its reaction filtrate revealed four aromatic resonances each integrating to one H, consistent with an asymmetric 1,3‐disubstituted anisole. From this we suggest that a secondary competing process is occurring. After initial metalation with LiTMP, rapid loss of aluminate LiAlF(TMP)*i*Bu_2_ occurs to generate a benzyne intermediate and TMPH, which can add across and trap the incipient benzyne affording 1‐(3‐methoxyphenyl)‐2,2,6,6‐tetramethylpiperidine (**I**; confirmed by aqueous work‐up and ^1^H and ^13^C NMR spectra of the resulting oil). Importantly, this process could not be arrested even at cryogenic temperatures. A control reaction between **1** and TMPH in C_6_D_6_ in a J. Young NMR tube established that **I** can be prepared via this pathway (see the Supporting Information). However, we cannot rule out the possibility that an autometalation process may also be contributing to the formation of the TMP‐substituted product. LiTMP is more nucleophilic than neutral TMPH, thus any present in solution (due to variations in stoichiometry or rapid generation of the benzyne before the LiTMP has all reacted) could also react with the benzyne, whereupon the generated lithiated species could deprotonate a second substrate molecule.

The decomposition pathways were probed further. Reaction between 3‐F‐anisole, LiTMP, and *i*Bu_2_AlTMP⋅THF was monitored over time in a J. Young NMR tube in C_6_D_6_ at room temperature. Initially the ^1^H NMR spectrum displayed signals corresponding to **1** and coproduct **I**, albeit after forming the metalated compound slowly decomposes. Confirming that coproduct TMPH, or potentially some unreacted LiTMP, is necessary for formation of **I**, the ^1^H NMR of **1** was recorded over 48 h. During this time resonances of **1** are essentially lost, and crucially no resonances corresponding to **I** emerge. Decomposition of **1** was confirmed operate via formation of a benzyne intermediate as demonstrated in an intentional trapping experiment by reacting either **1** or the in situ reaction mixture with 1,3‐diphenylisobenzofuran in hexane (Scheme 3). After filtration a solid was collected and identified by NMR spectroscopy as the known Diels–Alder cycloaddition product 1‐methoxy‐9‐10‐diphenyl‐9‐10‐epoxyanthracene in 86 % (from **1**) or 49 % (in situ mixture) yield. That **1**, an aryl aluminum decomposes via benzyne formation is interesting albeit not entirely unknown. A related process was seen during the sodium mediated *ortho*‐zincation of chlorobenzene using [TMEDA)⋅Na(μ‐TMP)(μ‐tBu)Zn(tBu)].^13^ Metalation of fluoroarenes using the LiTMP/*i*Bu_2_AlTMP TMT system is thus more complex than seen with other non‐fluorobenzene‐based systems. Specifically, trapping appears too sluggish to prevent benzyne formation and autometalation side reactions even at low temperature. Further, even metalated products are unstable in relatively innocent hydrocarbon solvents suggesting that the propensity of the aluminated species to eliminate Li‐F as part of an aluminate has a particularly deleterious effect on C‐Al bond stability.

**Scheme 3:**
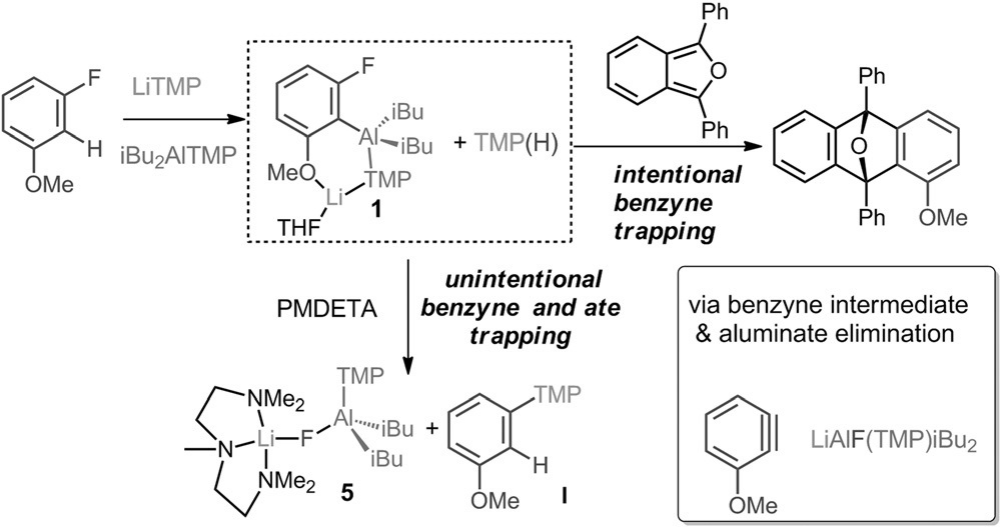
The effect of benzyne formation in the trans‐metal‐trapping procedure showing the target trapping product **1**, its trapped inorganic and organic decomposition products **5** and **I** respectively, and the Diels–Alder control reaction.

Next, *N*,*N*,*N*′,*N*′′*N*′′‐pentamethyldiethylenetriamine (PMDETA) was added to the reaction mixture containing LiTMP, *i*Bu_2_AlTMP, and a fluoroaromatic (3‐F‐anisole, fluorobenzene, or 1,3,5‐trifluorobenzene). We reasoned that the donor would draw Li away from the carbanionic centre, hastening transmetalation with Al. With 3‐F‐anisole, [PMDETA⋅Li(F)Al(*i*Bu)_2_TMP], **5** was obtained (Scheme 3). X‐ray crystallography revealed a CIP aluminate containing a μ‐F (expelled from the metalated anisole) between metal ions (Figure 2). Note that **5** can alternatively be made by adding PMDETA to **1** in hexane, whereas it cannot be accessed via co‐complexation of LiF, PMDETA, and the Al trap.

**Figure 2:**
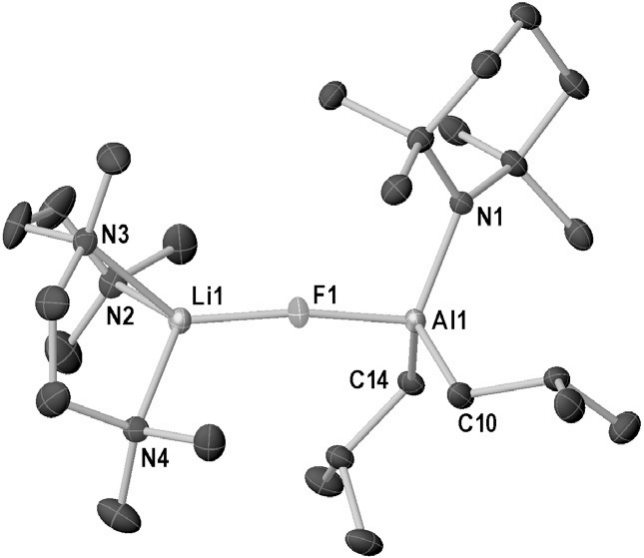
Molecular structure of **5**. All hydrogen atoms are omitted for clarity and thermal ellipsoids drawn at 30 % probability.

Importantly, **5** establishes that TMT can be used not only to trap carbanions but also to trap novel monomeric modifications of high lattice energy salts, that is, species that usually exist as polymeric or network lattices. This study unequivocally maps out structurally and spectroscopically the varied reaction pathways available to metallo‐fluoroarenes, by trapping both organic and inorganic components of decomposition alongside that of the target metalated product. Clearly the new TMT‐installed Al−C bonds are sensitive enough to facilitate decomposition by benzyne formation and concomitant ate elimination.

Next we turned to the gallium trapping reagent (Ga(CH_2_SiMe_3_)_3_). Reaction of LiTMP and Ga(CH_2_SiMe_3_)_3_ with fluorobenzene in hexane at −78 °C for one hour, followed by PMDETA addition gave a precipitate, that was recrystallized in 67 % yield. An X‐ray diffraction study of these crystals revealed 2‐Ga(CH_2_SiMe_3_)_3_‐1‐F‐C_6_H_4_⋅Li(PMDETA), **6** (see the Supporting Information) proving that, as expected, fluorobenzene was selectively metalated *ortho* to the F substituent (Ga1−C1 2.051(3) Å). Interestingly, this distance is shorter than the Al–C_Ar_ distances in **1**–**4**, signifying enhanced Ga carbophilicity. The F atom interacts with a Li⋅PMDETA unit (F1−Li1 1.867(6) Å), resulting in a CIP structure. The ^1^H NMR spectrum of **6** in C_6_D_6_ displayed four aromatic resonances consistent with the solid‐state arrangement. The ^19^F NMR spectrum displays a singlet at −111.35 ppm whereas the ^7^Li NMR spectrum has two singlets at 0.52 and −0.22 ppm with a broad featureless hump in‐between suggestive of a fluxional process.

Reaction scope was extended to 1,3‐difluorobenzene (giving 2‐Ga(CH_2_SiMe_3_)_3_‐1,3‐F_2_‐C_6_H_3_⋅Li(PMDETA), **7**, 63 %), 1,3,5‐trifluorobenzene (giving (2‐Ga(CH_2_SiMe_3_)_3_‐1,3,5‐F_3_‐C_6_H_2_⋅Li(PMDETA), **8**, 58 %), and 1,3,4,5‐tetrafluorobenzene (giving (2‐Ga(CH_2_SiMe_3_)_3_‐1,3,4,5‐F_4_‐C_6_H_1_⋅Li(PMDETA), **9**, 70 %). In each case NMR data share the fluxional characteristics of **6**, and are in agreement with regioselective *ortho*‐metalation of the fluorinated arenes. Complexes **6**–**9** all crystallize as mixtures of two conformers as evidenced by NMR data in C_6_D_6_, though in [D_8_]THF they all adopt a single SSIP arrangement (see the Supporting Information for spectroscopic characterization). Each “gallation” proceeds in good isolated yield and leads to stable crystalline solids, in contrast to that observed with Al. Crystal structures of **8** (Figure 3) and **9** (see the Supporting Information) enabled a comparison of C_Ar_−Ga distances, which elongate with increased fluorination of the aromatic ring (**6** Ga−C1 2.051(3) Å, **8** Ga−C1 2.086(4) Å, and **9** Ga1−C1 (2.093(3) Å), in line with a reduced carbanionic character of the metalated carbon atom. Notably, **6**–**9** are the first structurally characterized examples prepared by metalation.

**Figure 3:**
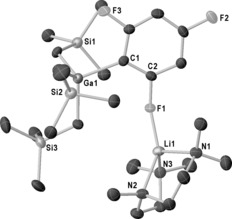
Molecular structure of **8**. All hydrogen atoms are omitted for clarity and thermal ellipsoids drawn at 30 % probability.

The solution stability of gallated fluoroarene **6** was probed by monitoring its ^1^H NMR spectra in C_6_D_6_ over time against ferrocene as an internal standard. In contrast to the aluminated fluoroarenes, ca. 77 % of **6** is intact after 48 h, and 65 % after 160 h, highlighting the profound synthetic advantage of the Ga trap over Al for stabilization of sensitive fluoroaromatic anions. Furthermore a sample of **8** in [D_8_]THF shows little sign of decomposition, even after 6 days, signifying that donor solvents enhance the stability of these systems, and evidence points to SSIP constitutions of these gallated structures. A related factor regarding the stability enhancement of the gallium complexes is the greater fluorophilicity of aluminum. Thus formation of Al−F bonds is promoted, hastening decomposition. A similar effect was reported by the group of Gessner in the stabilization of fluorine carbenoids with the heavier alkali metals.^14^

The final piece of this comparative study was to quantify how the distinct properties of these Al and Ga TMT systems would affect onward reactivity in an organic application. We chose a Pd(PPh_3_)_4_‐catalyzed cross‐coupling reaction between the metalated TMT products of 1,3,5‐trifluorobenzene with benzoyl chloride (Table 1). Note that LiTMP on its own proved ineffective in this reaction over a variety of conditions. The aluminated product **3** gave poor yields of ketone **10** (6–8 %) with ^19^F NMR spectra of isolated solid from the quench reaction in [D_8_]THF solution implying several F‐containing side products. In contrast, the analogous gallated product **8** reacted with benzoyl chloride more efficiently affording a best yield of **10** of 80 % as quantified by NMR studies using ferrocene as internal standard. Hydrolysis at the onset of the reaction, presumably through moisture contamination, appears to be the only side reaction (see the Supporting Information for experimental details). Though Huang and co‐workers have previously prepared ketones in good yield from benzoyl chloride and assorted lithium tetraorganogallates without a catalyst, the transferred nucleophiles were much less sensitive than the fluorinated examples probed here.^15^ In our case reactions were more efficient with the catalyst. Note, however, that examples of organogallium participation in organic synthesis is relatively uncommon,^16^ and furthermore, in cross‐coupling chemistry it is exceptionally rare.^15^, ^17^

**Table 1:** Metalation reactions (using Al and Ga traps), and subsequent cross‐coupling with benzoyl chloride and Pd(PPh_3_)_4_. 
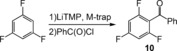

	M‐trap		Yield [%]^[a]^		*T* [°C]	
	None		0–20^[b]^		25^[c]^	
	*i*Bu_2_Al(TMP)		<10^[d]^		25	
	Ga(CH_2_SiMe_3_)_3_		79^[d}^		70	

[a] Yield of product determined by ^1^H NMR of the hydrolyzed mixture with ferrocene as an internal standard. [b] 5 mol % Pd(PPh_3_)_4_ does not improve the yield. [c] Reaction at room temperature results in complete decomposition of the substrate. Reaction at −78 °C afforded the product in 20 %. [d] 5 mol % of Pd(PPh_3_)_4_.

In conclusion, this study has (i) shown the ability of TMT to generate and stabilize sensitive fluoroaromatic carbanions, (ii) extended TMT for the trapping of molecular forms of inorganic salts, (iii) unravelled key complex decomposition pathways involved in metalation of fluoroarenes, and (iv) established the greater robustness of arylgallium intermediates versus arylaluminum species thus opening potential new synthetic uses for the heavier group 13 metal.

*Dedicated to Professor Snieckus on the occasion of his 80th birthday*

## Conflict of interest

The authors declare no conflict of interest.

## Supporting information

As a service to our authors and readers, this journal provides supporting information supplied by the authors. Such materials are peer reviewed and may be re‐organized for online delivery, but are not copy‐edited or typeset. Technical support issues arising from supporting information (other than missing files) should be addressed to the authors.

Supplementary
