# Supplementary material for: LiTMP Trans‐Metal‐Trapping of Fluorinated Aromatic Molecules: A Comparative Study of Aluminum and Gallium Carbanion Traps
Source: Angew Chem Int Ed Engl. 2017 Jul 17;56(32):9566–70. doi: 10.1002/anie.201706064 (PMC5577511; doi:10.1002/anie.201706064)
Supplement: Supplementary file 1 — Supplementary [file ANIE-56-9566-s001.pdf]

Angewandte Chemie
Eine Zeitschrift der Gesellschaft Deutscher Chemiker

Supporting Information

LiTMP Trans-Metal-Trapping of Fluorinated Aromatic Molecules: A Comparative Study of Aluminum and Gallium Carbanion Traps

Ross McLellan, Marina Uzelac, Alan R. Kennedy, Eva Hevia, and Robert E. Mulvey**

anie_201706064_sm_miscellaneous_information.pdf

Table of Contents **Pg**

General experimental considerations	3
Preparation and characterisation of **1**-**4**	4
Isolation and characterisation of 1-(3-methoxyphenyl)-2,2,6,6-tetramethylpiperidine **I**	7
Decomposition studies of [2-{(*i*Bu)$_2$Al(μ-TMP)Li·THF}-3-fluoroanisyl] **1**	8
Benzyne trapping of [2-{(*i*Bu)$_2$Al(-TMP)Li·THF}-3-fluoroanisyl] **1**	10
Preparation and characterisation of [PMDETA·Li(F)Al(*i*Bu)$_2$TMP] **5**	11
Preparation of **6**-**9**	16
Characterisation of 2-Ga(CH_2SiMe_3)$_3$-1-F-C_6H_4·Li(PMDETA), **6**	17
Characterisation of 2-Ga(CH_2SiMe_3)$_3$-1,3-F_2-C_6H_3·Li(PMDETA), **7**	22
Characterisation of (2-Ga(CH_2SiMe_3)$_3$-1,3,5-F_3-C_6H_2·Li(PMDETA), **8**	27
Characterisation of (2-Ga(CH_2SiMe_3)$_3$-1,3,4,5-F_4-C_6H_1·Li(PMDETA), **9**	32
Decomposition studies of **6** and **8**	34
Electrophilic quenching studies	36
Characterisation of phenyl(2,4,6-trifluorophenyl)methanone, **10**	40
Molecular structures of **1**-**6**, **8** and **9**	43
Crystallographic data for **1**-**6**, **8** and **9**	44
References	46

General experimental considerations

All reactions and manipulations were conducted under a protective argon atmosphere using either standard Schlenk techniques or an MBraun glove box fitted with a gas purification and recirculation unit. NMR experiments were conducted in J. Youngs tubes oven dried and flushed with Argon prior to use. Hexane, toluene and THF were dried by heating to reflux over sodium benzophenone ketyl and then distilled under nitrogen prior to use. All other reagents were purchased commercially from Sigma-Aldrich or Fluorochem, dried via distillation from the appropriate drying agent prior to use. LiTMP,[1] iBu_2AlTMP[2] and $Ga(CH_2Me_3)_3$[3] were prepared as previously described or by slight variations thereof.

NMR Spectroscopy NMR spectra were recorded on a Bruker AV3 or AV 400 MHz spectrometer operating at 400.13 MHz for ^1H, 128.38 MHz for ^{19}F, 376.46 MHz for ^7Li and 100.62 MHz for ^{13}C. All ^{13}C spectra were proton decoupled. ^1H and ^{13}C NMR spectra were referenced against the appropriate solvent signal. ^7Li NMR spectra were referenced against LiCl in D_2O at 0.00 ppm and ^{19}F spectra were referenced against $CFCl_3$ 0.00 ppm.

X-ray Crystallography Crystallographic data were collected on Oxford Diffraction instruments with Mo Kα radiation (λ = 0.71073 Å) or Cu Kα radiation (λ = 1.54184 Å). Structures were solved using SHELXS-97[4] or OLEX2,[5] while refinement was carried out on F2 against all independent reflections by the full matrix least-squares method using the SHELXL-97 program or by the GaussNewton algorithm using OLEX2. All non-hydrogen atoms were refined using anisotropic thermal parameters. Selected crystallographic details and refinement details are provided in table S1. CCDC 1555587-1555594 contains the supplementary crystallographic data for this structure. These data can be obtained free of charge from the Cambridge Crystallographic Data Centre via www.ccdc.cam.ac.uk/data_request/cif.

Preparation of 1-4

Preparation of [2-{(*i*Bu)$_2$Al(μ-TMP)Li·THF}-3-fluoroanisyl] 1: A mixture of LiTMP (147 mg, 1 mmol) and iBu$_2$AlTMP (281 mg, 1 mmol) in hexane 8 mL were cooled to -78 °C and 3-F-anisole (0.11 ml, 1 mmol) was added via syringe. The reaction was stirred for 1 hour causing formation of a white precipitate. THF was added dropwise until the precipitate dissolved and placed at -20 °C overnight, resulting in formation of crystals of **1** (228 mg, 0.47 mmol, 47 % yield).

Preparation of 2-4: Complexes **2**-**4** were prepared by a directly analogous procedure.

Characterisation of [2-{(*i*Bu)$_2$Al(μ-TMP)Li·THF}-3-fluoroanisyl] 1

Elemental analysis (%) calculated for $C_{28}H_{50}Al_1F_1Li_1N_1O_2$: C 69.25, H 10.38, N 2.88; found: C 68.18, H 9.92, N 2.54.

^{1}H NMR (400.1 MHz, C_6D_6 300K): δ 6.94 (1H, td, Ar*H*), 6.74 (1H, m, Ar*H*), 6.19 (1H, d, Ar*H*), 3.28 (3H, s O*Me*), 3.01 (4H, m, THF), 2.40 (2H, m, iBu methine*H)*, 1.89 (1H, m, TMP γ-*H*), 1.68 (6H, s, TMP *Me*), 1.56 (2H, m, TMP *β-H*), 1.54 (1H, m, TMP *γ-H*) 1.45(6H, d, iBu *Me*), 1.40 (6H, s, TMP *Me*), 1.16 (6H, d, iBu *Me*), 1.08 (6H, m, *THF*), 0.91 (2H, m, TMP *β-H*). 0.87 (2H, m, iBu methylene*H*) and 0.70 ppm (2H, m, iBu methylene*H*).

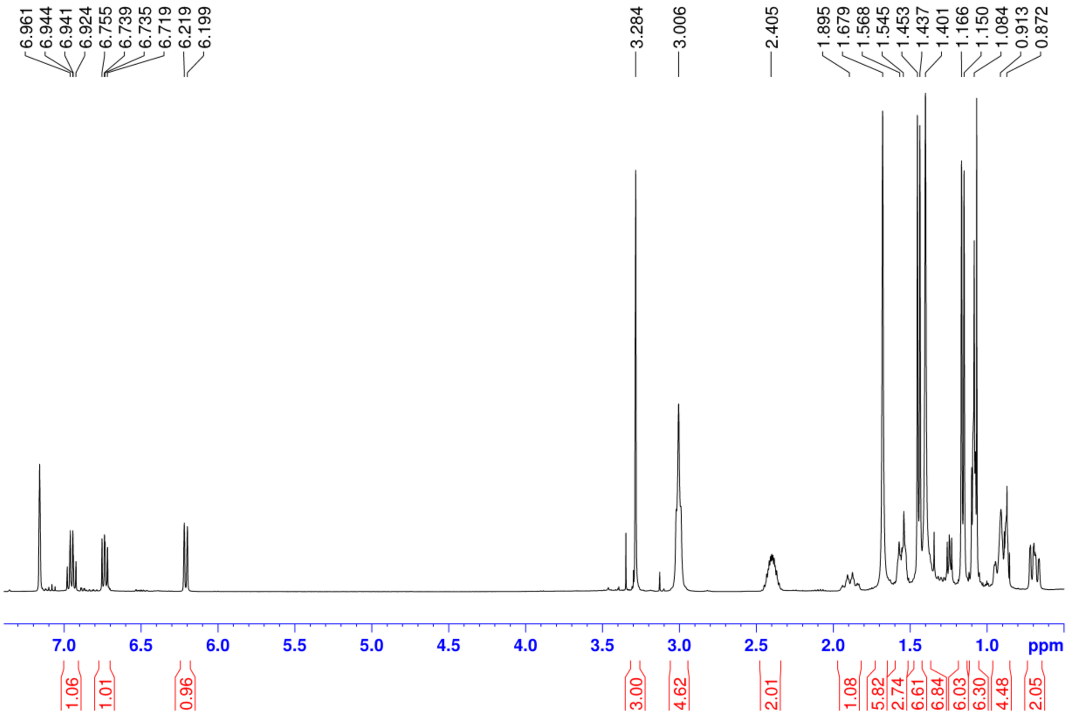

Figure S1 ^{1}H NMR spectrum of [2-{(*i*Bu)$_2$Al(μ-TMP)Li·THF}-3-fluoroanisyl] **1** in C_6D_6

^{13}C NMR (100.6 MHz, C_6D_6 300K) δ 170.4 (d, Ar*C*-F, $^1J_{C-F}$ 236 Hz),164.3 (d, Ar*C*-OMe, $^3J_{C-F}$ 24.6 Hz), 129.5 (d, Ar*C*H, $^3J_{C-F}$ 9.2 Hz), 110.9 (d, Ar*C*H, $^2J_{C-F}$ 32.8 Hz), 105.8 (d, Ar*C*H, $^4J_{C-F}$ 2.9 Hz), 68.5 (s, *THF*), 55.3 (s, O*Me*), 53.3 (s, TMP α-*C*), 44.3 (s, TMP β-*C*), 36.7 (s, TMP *Me*), 31.2 (s, iBu *Me*), 30.3 (s, TMP *Me*), 29.6 (br. S, iBu *C*H_2), 27.6 (s, iBu *Me*), 27.6 (s, iBu *C*H), 25.0 (s, *THF*) and 18.8 ppm (s, TMP γ-C).

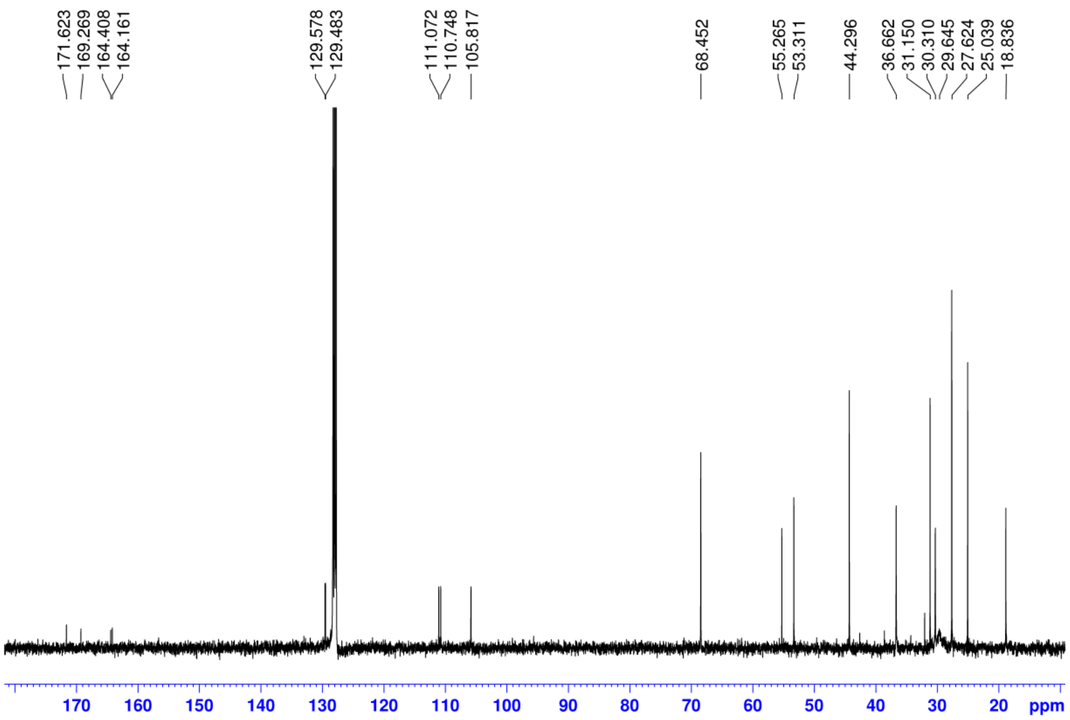

Figure S2 ^{13}C NMR spectrum of [2-{(*i*Bu)$_2$Al(μ-TMP)Li·THF}-3-fluoroanisyl] **1** in C_6D_6.

^{7}Li NMR (155.5 MHz, C_6D_6 300K): δ 0.43 ppm, singlet.

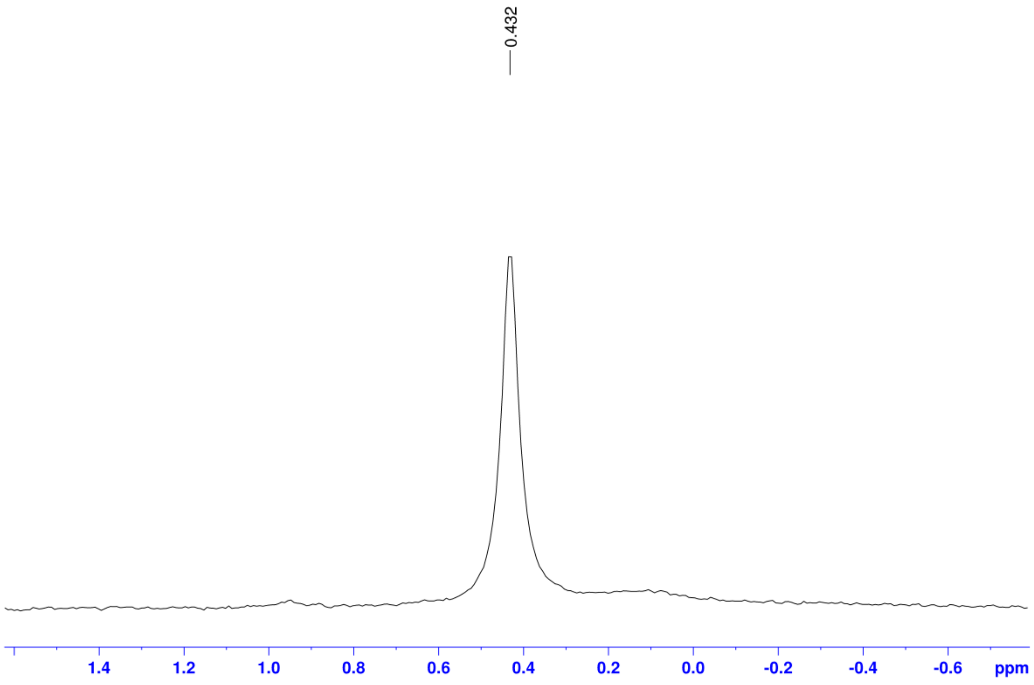

Figure S3 ^{7}Li NMR spectrum of [2-{(*i*Bu)$_2$Al(μ-TMP)Li·THF}-3-fluoroanisyl] **1** in C_6D_6.

^{19}F NMR (376.5 MHz, C_6D_6 300K): δ -85.05 ppm, singlet.

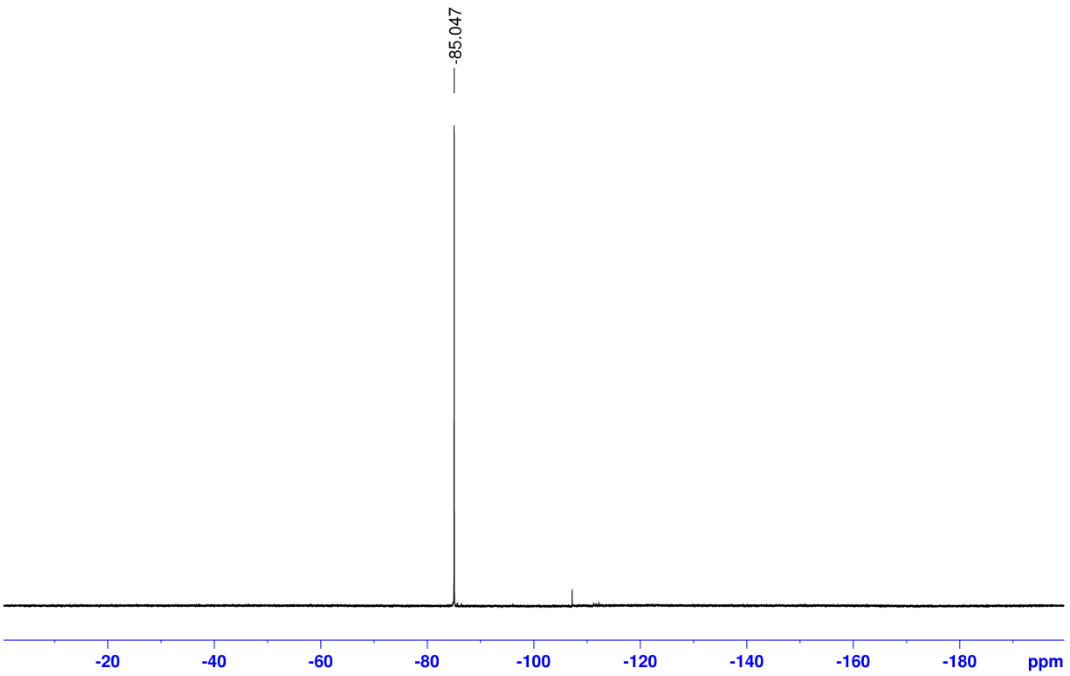

Figure S4 ^{19}F NMR spectrum of [2-{(*i*Bu)$_2$Al(μ-TMP)Li·THF}-3-fluoroanisyl] **1** in C_6D_6.

Analysis of reaction filtrate

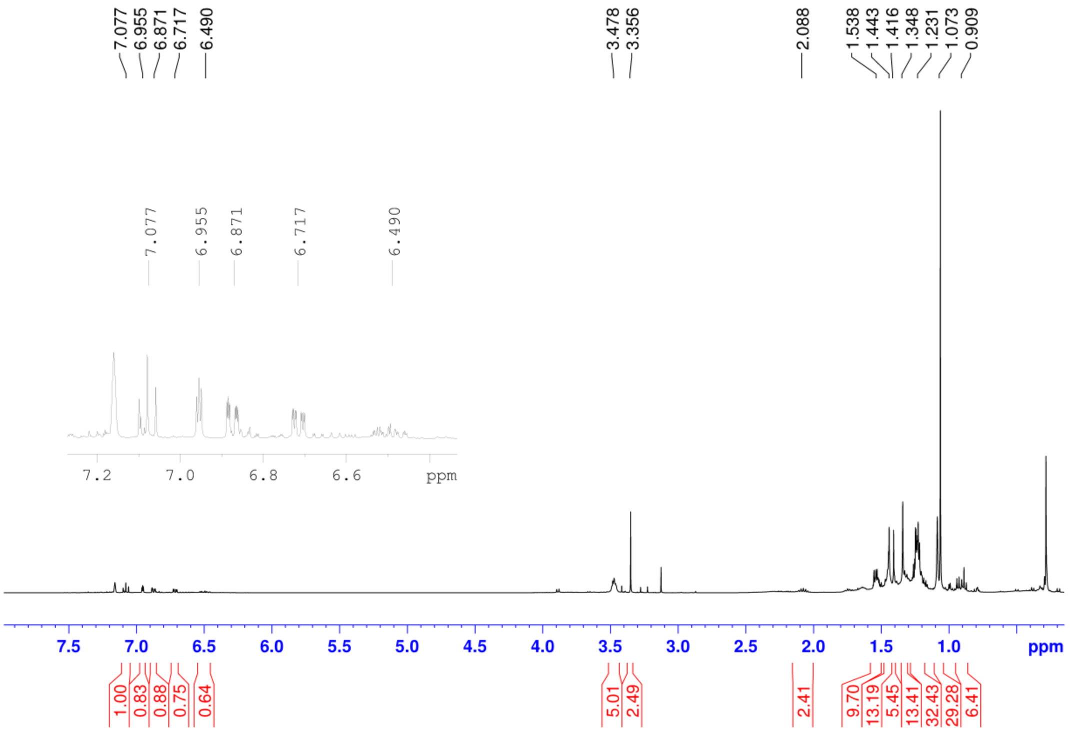

Figure S5 ^{1}H NMR spectrum of **1** reaction filtrate in C_6D_6.

Isolation of coproduct 1-(3-methoxyphenyl)-2,2,6,6-tetramethylpiperidine (I)

EtOAc (10 mL) and H_2O (10 mL) was added to the reaction filtrate and the organic fraction collected. The organic layer was washed twice with water and then brine, then dried over $MgSO_4$. Removal of all volatiles resulted in a colourless oil.

^{1}H NMR (400.1 MHz, $CDCl_3$ 300K): δ 7.17 (1H, t, Ar*H*), 6.83 (1H, td, Ar*H*), 6.79 (2H, m, Ar*H*), 3.81 (3H, s O*Me*), 1.74 (2H, m, TMP γ-*H*), 1.58 (4H, m, TMP β-*H*) and 1.05 ppm (12H, m, TMP *Me*).

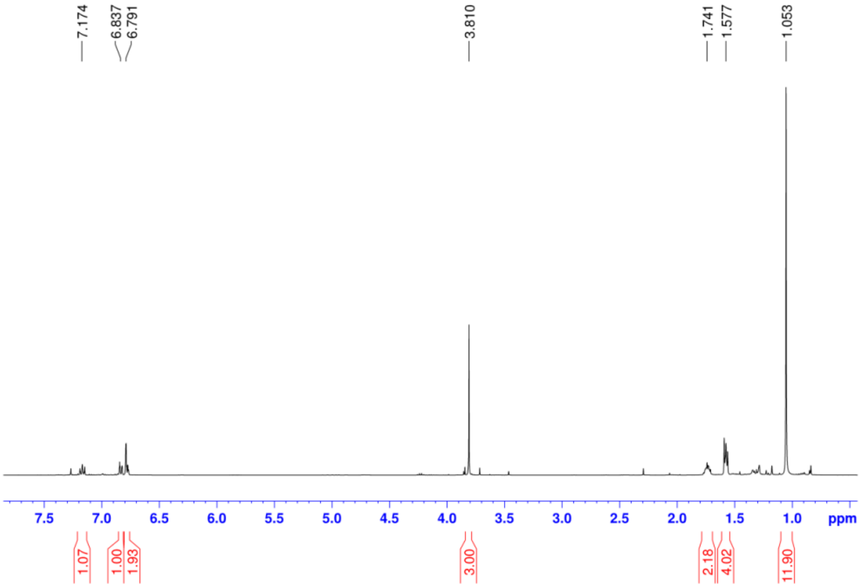

Figure S6 ^{1}H NMR spectrum of isolated 1-(3-methoxyphenyl)-2,2,6,6-tetramethylpiperidine (**I**) in $CDCl_3$

^{13}C NMR (100.6 MHz, $CDCl_3$ 300K) δ 159.0 (s, Ar*C* quaternary), 148.1 (s, Ar*C* quaternary), 127.7 (s, Ar*C*H), 126.6 (s, Ar*C*H), 120.2 (d, Ar*C*H), 110.7 (s, Ar*C*H), 55.1 (s, O*Me*), 54.0 (s, TMP α-*C*), 42.3 (s, TMP γ-*C*), 29.6 (s, TMP *Me*) and 18.3 ppm (s, TMP β-C).

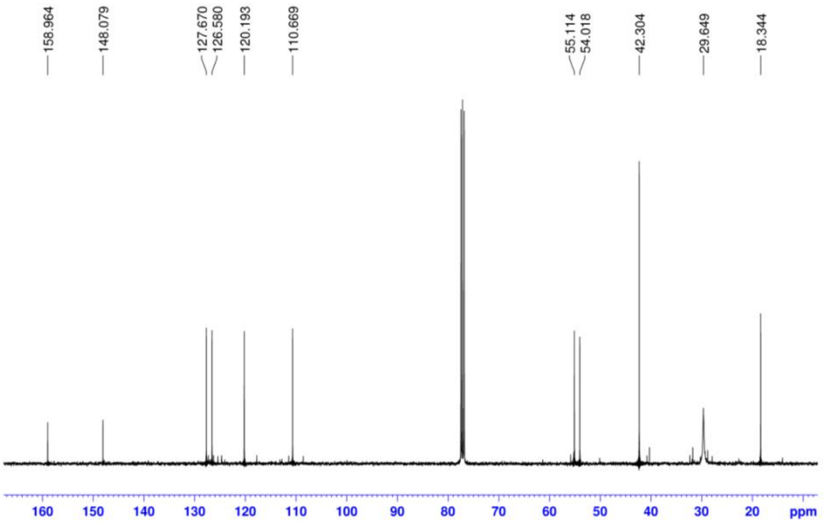

Figure S7 ^{13}C NMR spectrum of isolated 1-(3-methoxyphenyl)-2,2,6,6-tetramethylpiperidine (**I**) in $CDCl_3$.

Reaction of 1 and TMPH in C_6D_6 at room temperature

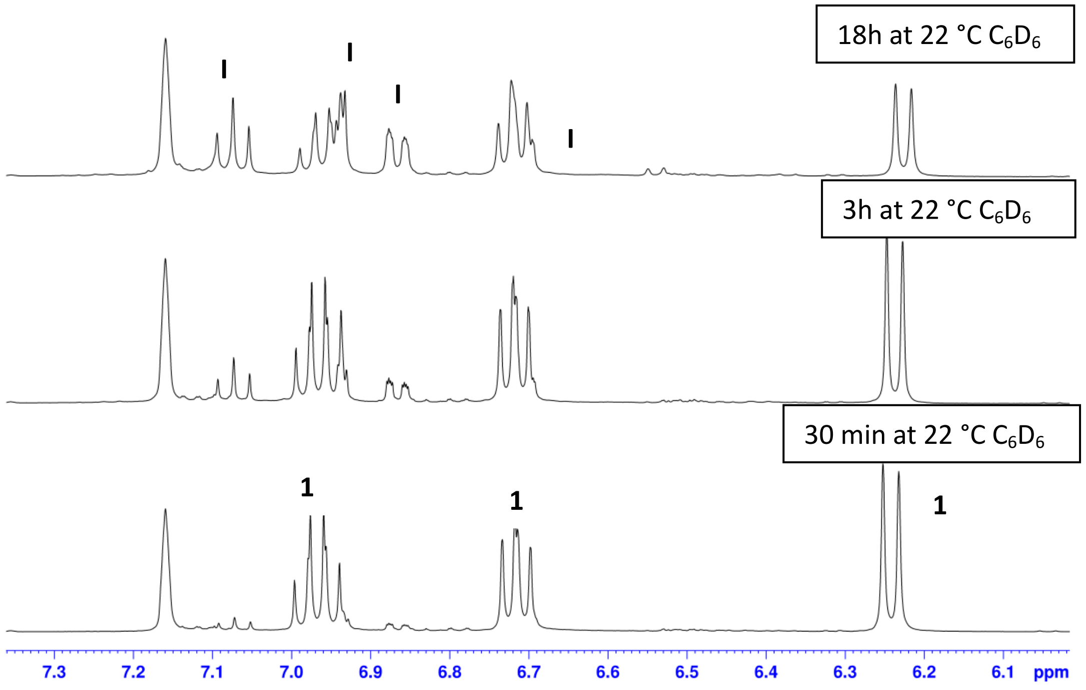

Figure S8 ^{1}H NMR spectra (aromatic region) following formation of **I** over time.

***In situ* reaction of LiTMP, *i*Bu$_2$AlTMP and 3-F-anisole at room temperature in C_6D_6 in a J. Young NMR tube.**

Reaction displays formation of both **1** and **I** followed by the gradual decomposition of **1**.

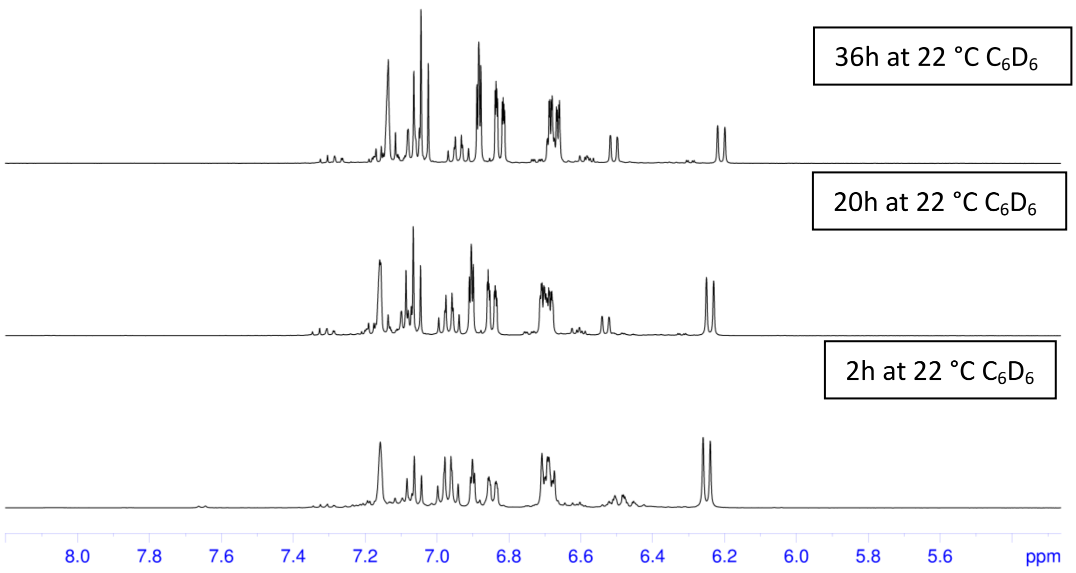

Figure S9 ^{1}H NMR spectra following the formation of **1** and **I** over time.

Reaction of 1 over time at room temperature in C_6D_6 in a J. Young NMR tube.

Reaction shows that over time **1** decomposes but does not form **I**. The inference here is that ladditional TMPH/LiTMP is required to form **I** from **1**.

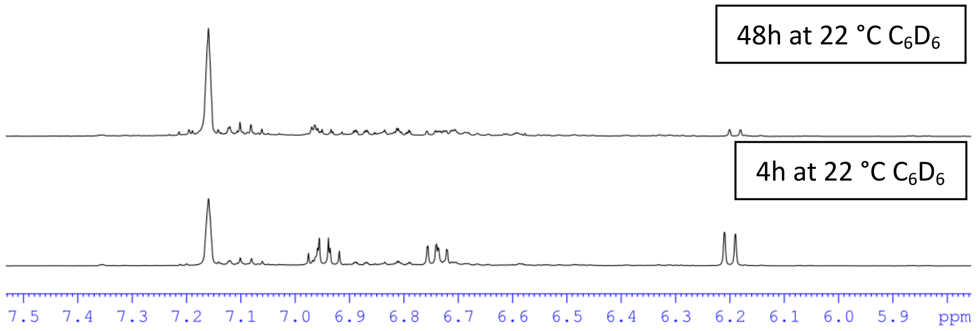

Figure S10 ^{1}H NMR spectra following the decomposition of **1** over time.

Benzyne trapping studies

Reaction of either *in situ* reaction mixture or **1** with diphenylisobenzofuran results in formation of Diels-Alder cycloaddition product 1-methoxy-9-10-diphenyl-9-10-epoxyanthracene.

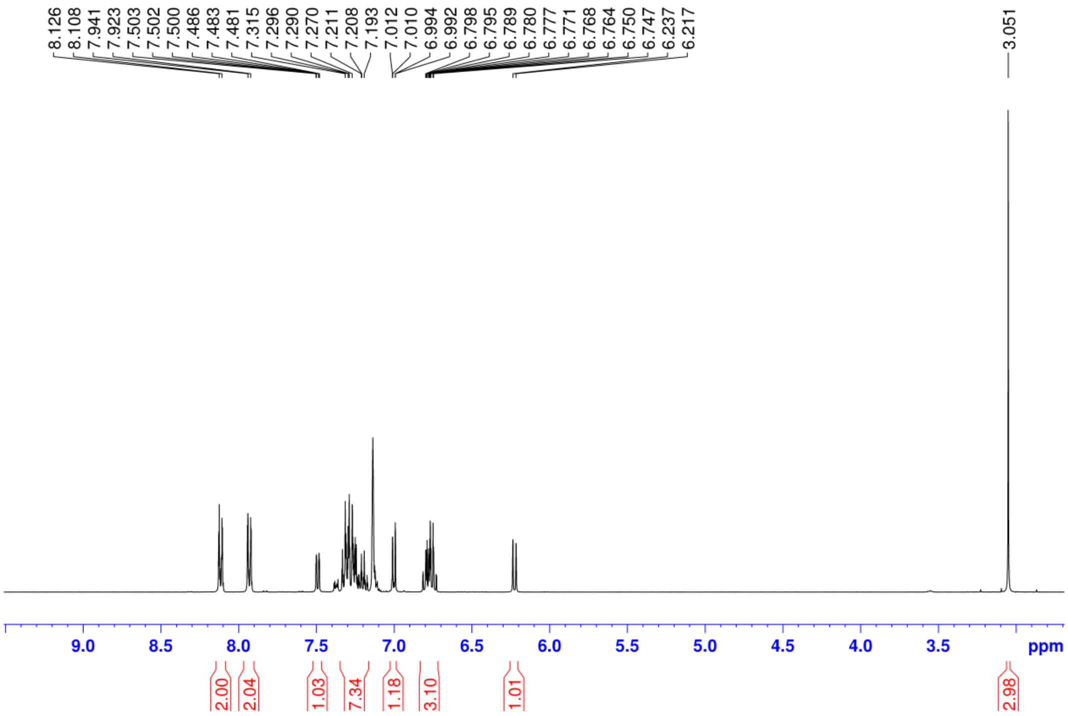

Figure S11 ^{1}H NMR spectrum of 1-methoxy-9-10-diphenyl-9-10-epoxyanthracene in C_6D_6.

Preparation of [PMDETA·Li(F)Al(*i*Bu)$_2$TMP] 5: A mixture of LiTMP (147 mg, 1 mmol) and iBu$_2$AlTMP (281 mg, 1 mmol) in hexane 8 mL were cooled to -78 °C and 3-F-anisole (0.11 ml, 1 mmol) was added via syringe. PMDETA (0.21 mL, 1 mmol) was then added and the reaction was stirred for 1 hour. causing formation of a pale orange oil. Single crystals of **5** were grown after placing the reaction mixture at -30 °C for several days. (264 mg, 0.55 mmol, 55 % yield).

Elemental analysis (%) calculated for $C_{26}H_{59}Al_1F_1Li_1N_4$: C 64.96, H 12.37, N 11.66; found: C 64.91, H 12.24, N 12.01.

Characterisation of [PMDETA·Li(F)Al(*i*Bu)$_2$TMP] 5 in C_6D_6

^{1}H NMR (400.1 MHz, C_6D_6 300K): δ 2.33 (2H, m, iBu methine*H)*, 1.97 (2H, m, TMP γ-*H*), 1.91 (3H, s, PMDETA *Me*), 1.74 (4H, m, TMP β-*H*), 1.69 (12H, s, TMP *Me*) 1.56 (4H, br. s,PMDETA *C*H$_2$), 1.46 (12H, s, iBu *Me*), 0.38 (2H, m, iBu methylene*H*) and 0.30 ppm (2H, m, iBu methylene*H*).

Here the resonances corresponding to PMDETA are very broad and uninterpretable.

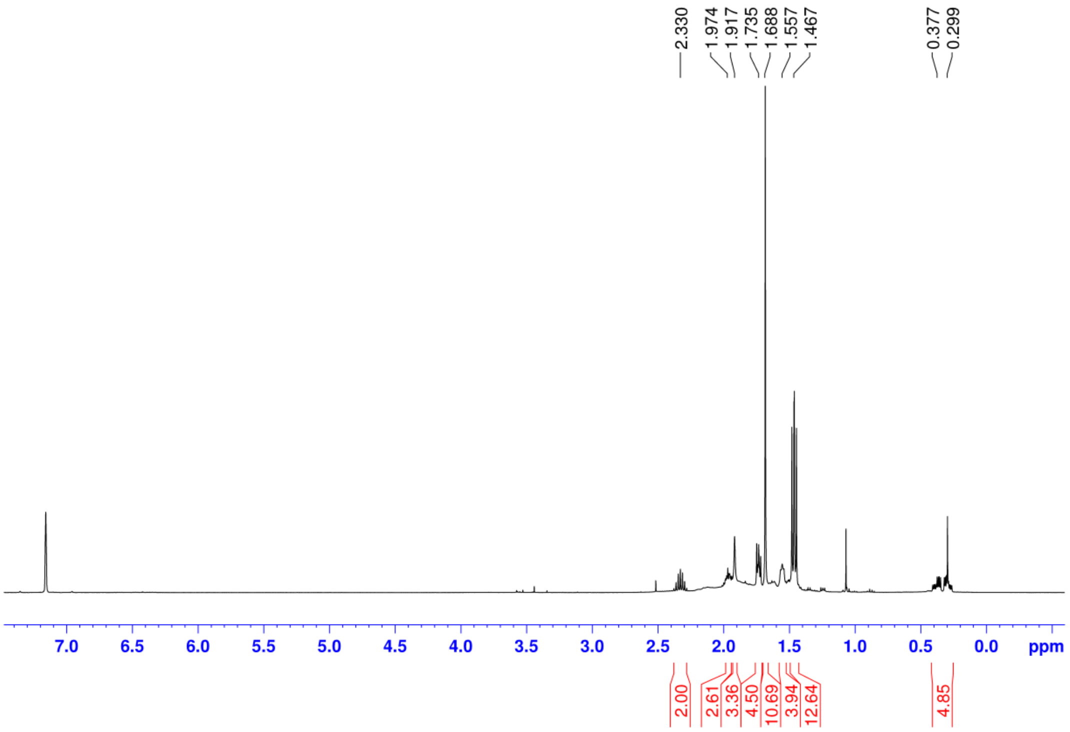

Figure S12 ^{1}H NMR spectrum of [PMDETA·Li(F)Al(*i*Bu)$_2$TMP] **5** in C_6D_6.

^{13}C NMR (100.6 MHz, C_6D_6 300K) δ 56.9 (s, TMP α-*C*), 53.0 (s, PMDETA CH_2), 51.7 (s, PMDETA CH_2), 45.4 (br. s, PMDETA) 43.9 (s, TMP β-*C*), 34.5 (s, TMP *Me*), 29.9 (s, iBu *Me*), 29.6 (s, iBu *Me*), 27.8 (s, iBu methine *C*) and 19.8 ppm (s, TMP γ-*C*).

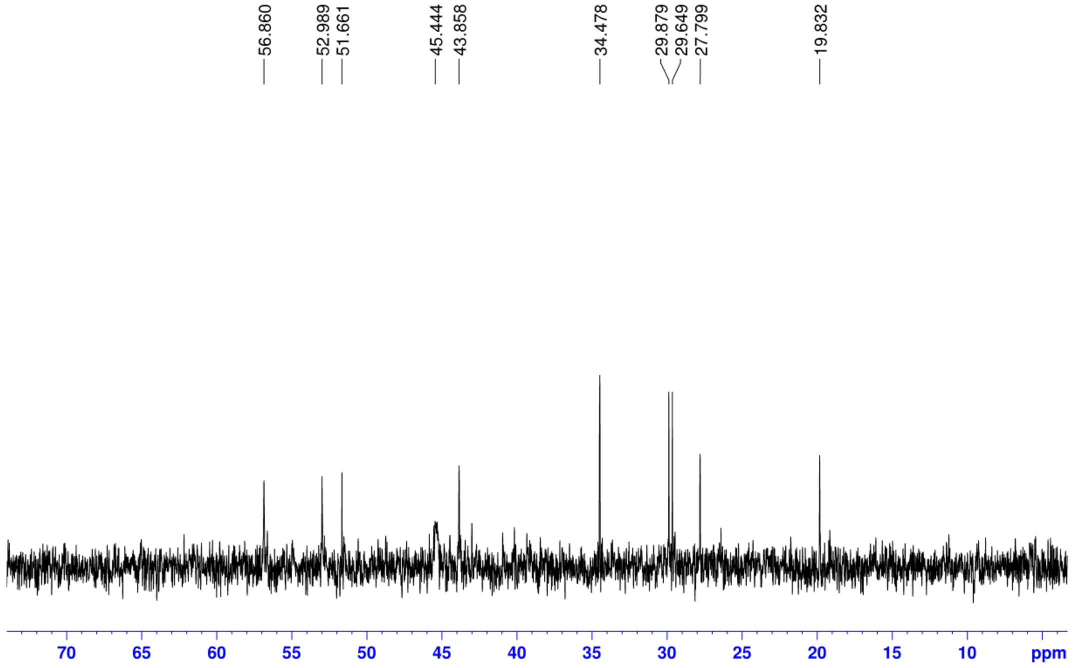

Figure S13 ^{13}C NMR spectrum of [PMDETA·Li(F)Al(*i*Bu)$_2$TMP] **5** in C_6D_6.

^{7}Li NMR (155.5 MHz, C_6D_6 300K): doublet centred on δ 0.4 ppm, $^{1}J_{Li\text{-}F}$ 96.8 Hz.

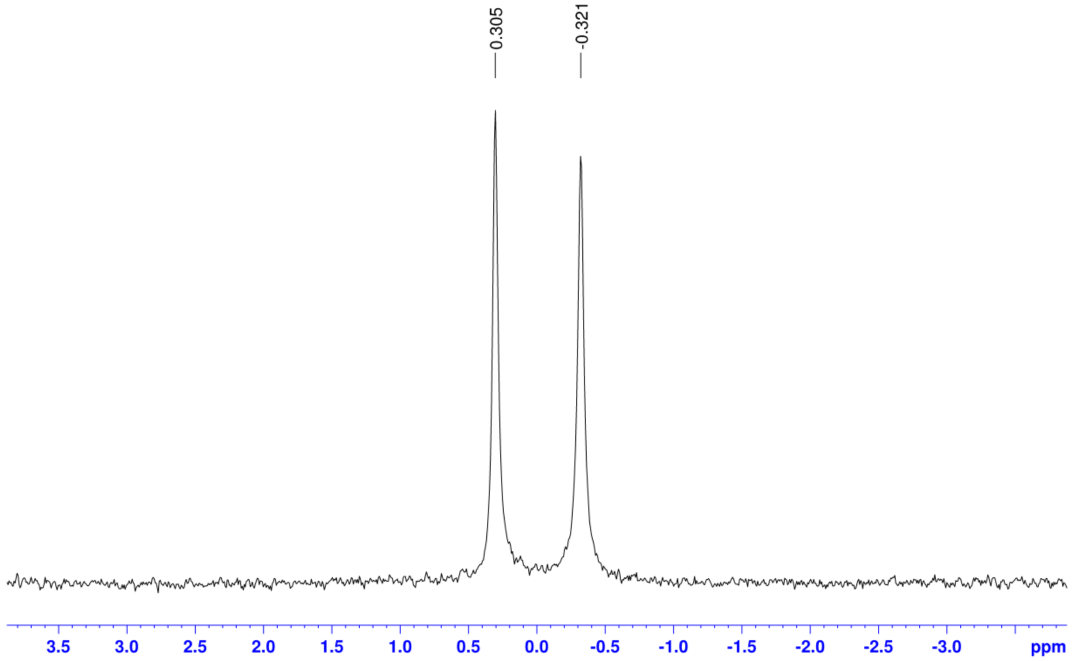

Figure S14 ^{7}Li NMR spectrum of [PMDETA·Li(F)Al(*i*Bu)$_2$TMP] **5** in C_6D_6.

^{19}F NMR (376.5 MHz, C_6D_6 300K): δ -161.3 ppm, quartet $^{1}J_{F\text{-}Li}$ 94.3 Hz.

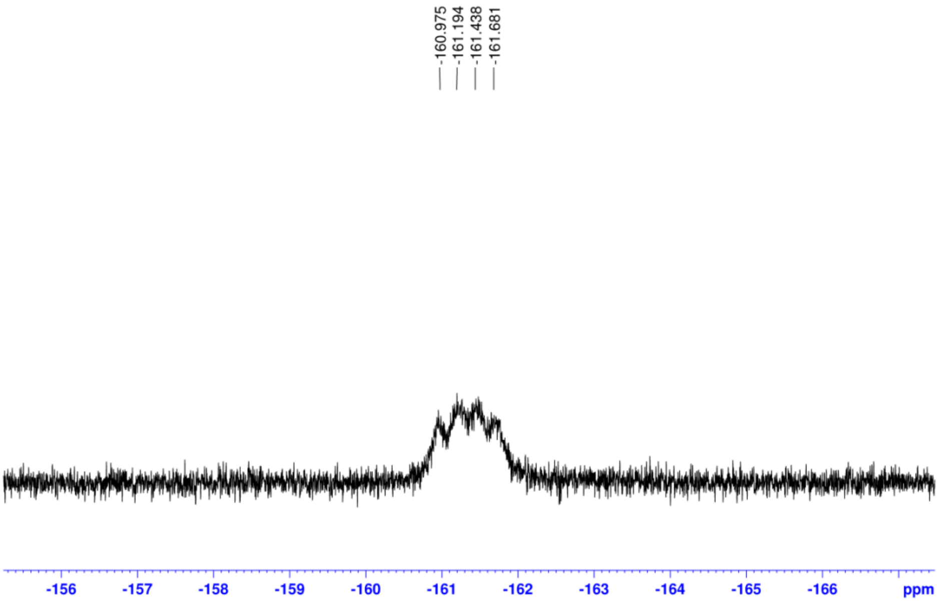

Figure S15 ^{19}F NMR spectrum of [PMDETA·Li(F)Al(*i*Bu)$_2$TMP] **5** in C_6D_6.

Characterisation of [PMDETA·Li(F)Al(*i*Bu)$_2$TMP] 5 in *d*$_8$-THF

^{1}H NMR (400.1 MHz, *d*$_8$-THF 300K): δ 2.44 (4H, m, PMDETA C*H*$_2$), 2.32 (4H, m, PMDETA C*H*$_2$), 2.22 (3H, S, PMDETA C*H*$_3$), 2.19 (12H, S, PMDETA C*H*$_3$), 1.84 (2H, septet, iBu methine*H)*, 1.56 (2H, m, TMP γ-*H*), 1.29 (4H, m, TMP *β-H*), 1.22 (12H, s, TMP *Me*) 0.91 (12H, s, iBu *Me*) and -0.22 ppm (4H, m, iBu methylene*H*).

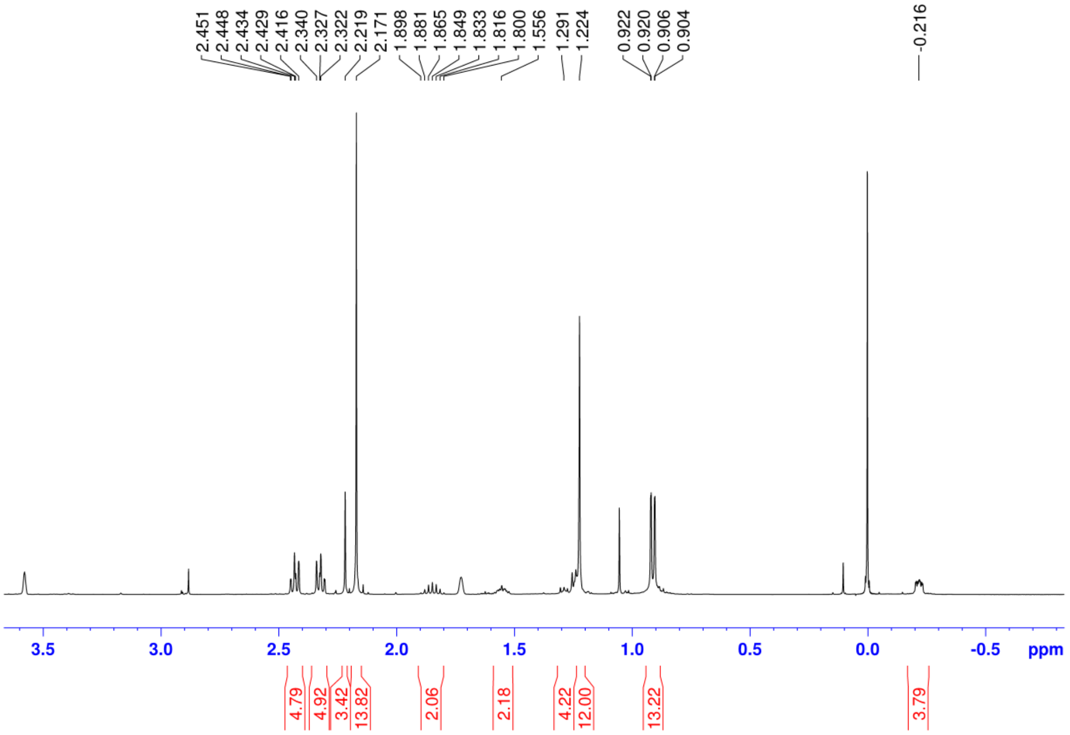

Figure S16 ^{1}H NMR spectrum of [PMDETA·Li(F)Al(*i*Bu)$_2$TMP] **5** in *d*$_8$-THF.

^{13}C NMR (100.6 MHz, $CDCl_3$ 300K) δ 58.6 (s, PMDETA CH_2), 56.9 (s, PMDETA CH_2), 51.6 (s, TMP α-C), 46.0 (s, PMDETA *Me*), 44.0 (s, TMP β-C), 43.4 (s, PMDETA *Me*), 34.2 (s, TMP, *Me*), 30.2 (s, iBu CH_2), 29.5 (s, iBu *Me*), 29.3 (s, iBu *Me*), 27.7 (s, iBu methine *C*) and 19.9 ppm (s, TMP γ-C).

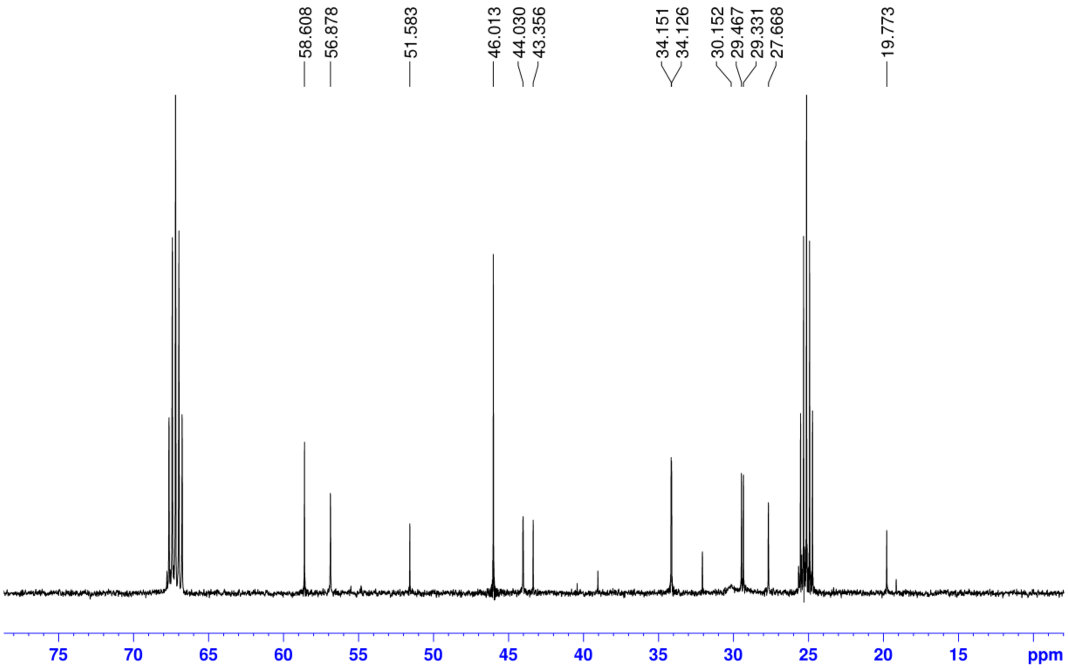

Figure S17 ^{13}C NMR spectrum of [PMDETA·Li(F)Al(*i*Bu)$_2$TMP] **5** in d_8-THF.

^7Li NMR (155.5 MHz, d_8-THF, 300K): singlet δ 1.5 ppm.

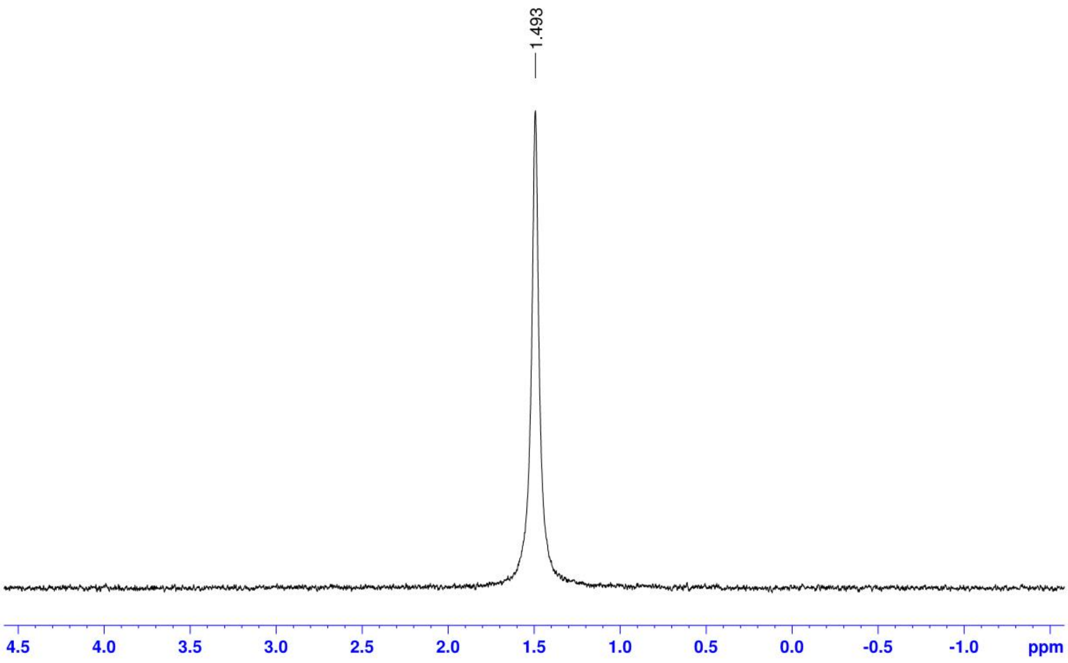

Figure S18 ^7Li NMR spectrum of [PMDETA·Li(F)Al(*i*Bu)$_2$TMP] **5** in d_8-THF.

^{19}F NMR (376.5 MHz, d_8-THF, 300K): broad singlet δ -160.7 ppm.

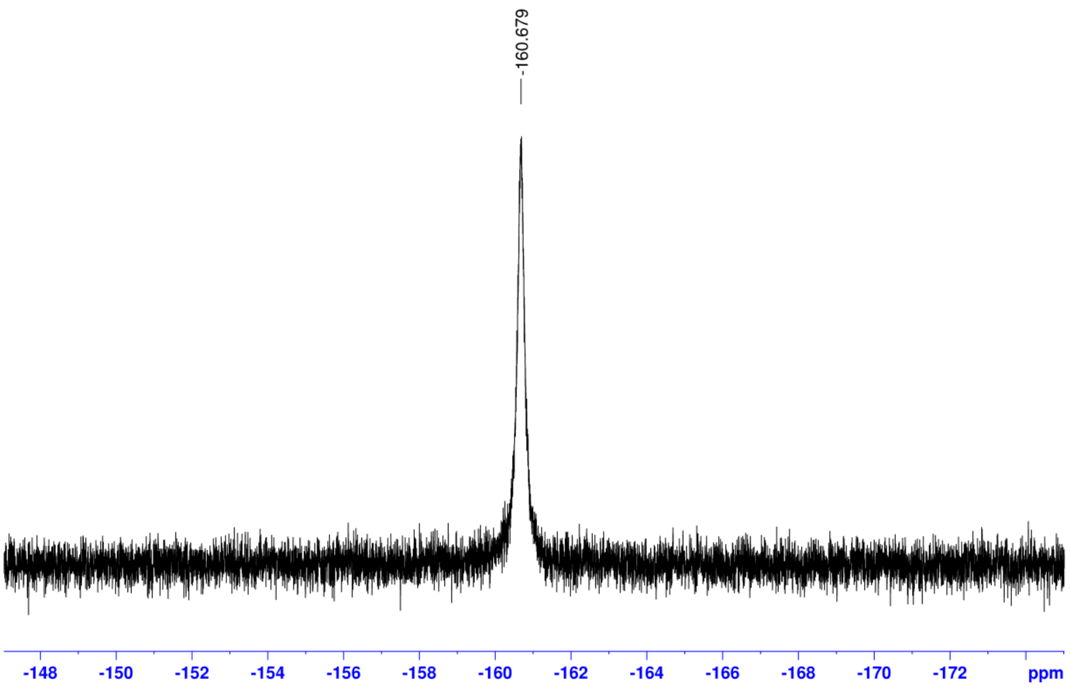

Figure S19 ^{19}F NMR spectrum of [PMDETA·Li(F)Al(*i*Bu)$_2$TMP] **5** in d_8-THF.

Preparation of 6-9

Preparation of 2-Ga(CH_2SiMe_3)$_3$-1-F-C_6H_4·Li(PMDETA) 6: A mixture of LiTMP (74 mg, 0.5 mmol) and Ga(CH_2SiMe_3)$_3$ (165 mg, 0.5 mmol) in hexane 8 mL were cooled to -78 °C and fluorobenzene (0.05 ml, 0.5 mmol) was added via syringe. The reaction was stirred for 1 hour then PMDETA (0.11 mL 0.5 mmol) was added and the reaction stirred for a further 30 mins. The resulting white precipitate was recrystallised by dropwise addition of toluene with gentle warming until a clear solution was obtained, then cooling at -20 °C overnight. (203 mg, 0.34 mmol, 67 % yield).

Elemental analysis (%) calculated for $C_{27}H_{60}Ga_1F_1Li_1N_3Si_3$: C 52.51, H 10.17, N 7.07; found: C 52.47, H 9.88, N 7.78.

Preparation of 7-9: Complexes **7**-**9** were prepared by a directly analogous procedure.

General comments on **6**-**9:** All 4 complexes were isolated as two isomeric arrangements. NMR studies in d_8-THF results in one solvent separated arrangement whereas in benzene the distinct conformers are retained. For example, warming a sample (here **9** see below) sharpens the two singlets in the ^{7}Li spectrum, thus it appears that, at higher temperature there are two distinct Li environments exchanging faster than the NMR timescale that in giving rise to two resonances contrasts with the solid state structure. This can be ascribed to either a CIP–SSIP process or two CIP scenarios, the first resembling the crystal structure arrangement, and the second includes a Li-π interaction with the aromatic ring. The latter would presumably relieve the congested environment observed in the solid state. Collecting ^{1}H NMR data of **6**-**9** in donor solvent, d_8-THF reveals that they all adopt a SSIP arrangement with a [Li(THF)$_4$][Ga(CH_2Me_3)$_3$ArF] formulation. Further, the ^{7}Li NMR spectrum shows only one clear sharp resonance in each case, giving credence to the situation of two distinct yet similar CIP isomers present in the ^{7}Li spectra in C_6D_6.

Characterisation of 2-Ga$(CH_2SiMe_3)_3$-1-F-C_6H_4·Li(PMDETA) 6 in C_6D_6

^{1}H NMR (400.1 MHz, C_6D_6 300K): δ 8.25 (1H, m, Ar*H*), 7.22 (1H, tdd, Ar*H*), 7.05 (1H, qd, Ar*H*), 6.43 (1H, td, Ar*H*), 1.87 (3H, br. s, PMDETA *Me*), 1.77 (12H,br. s, PMDETA *Me*), 1.57 (8H, br. s, PMDETA C*H*$_2$), 0.43 (27H, s, -CH_2Si*Me*$_3$) and -0.27 ppm (6H, s, -C*H*$_2$SiMe$_3$).

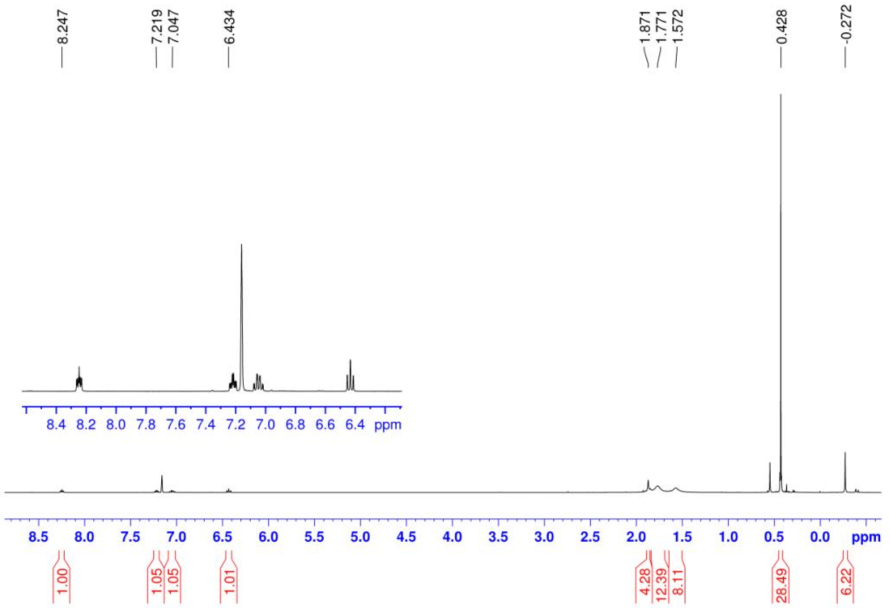

Figure S20 ^{1}H NMR spectrum of 2-Ga$(CH_2SiMe_3)_3$-1-F-C_6H_4·Li(PMDETA), **6** in C_6D_6.

^{13}C NMR (100.6 MHz, C_6D_6 300K) δ 170.7 (d, Ar*C*-F, $^1J_{C\text{-}F}$ 205.4 Hz),142.2 (d, Ar*C*-H, $^3J_{C\text{-}F}$ 28.6 Hz), 126.3 (d, Ar*C*H, $^3J_{C\text{-}F}$ 9.0 Hz), 124.9 (s, Ar*C*H), 110.9 (d, Ar*C*H, $^2J_{C\text{-}F}$ 34.2 Hz), 56.7 (s, PMDETA *C*H$_2$), 53.0 (s, PMDETA *C*H$_2$), 45.2 (s, PMDETA *Me*), 44.3 (s, PMDETA *Me*), 4.1 (s, -CH_2Si*Me*$_3$) and 1.2 ppm (s, -*C*H$_2$SiMe$_3$).

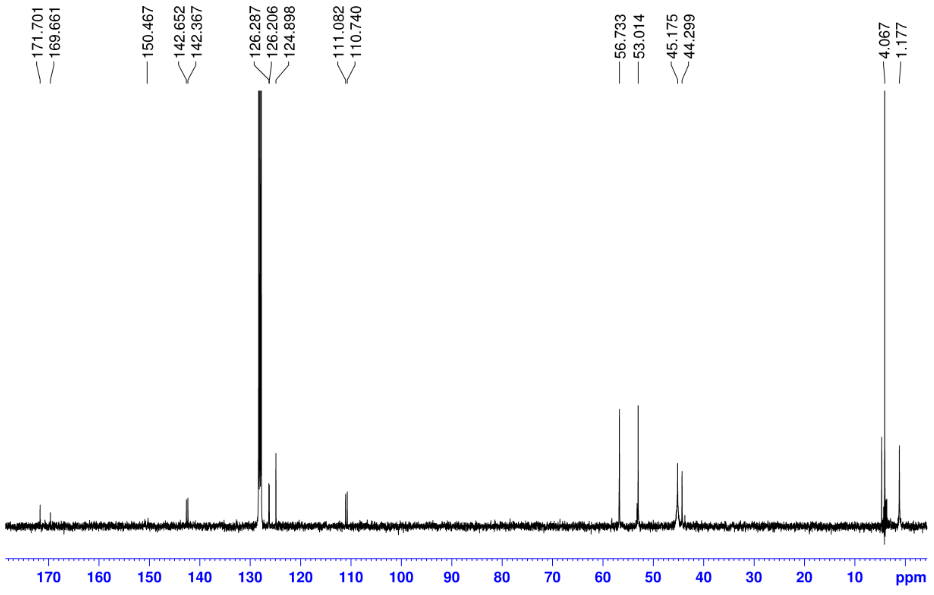

Figure S21 ^{13}C NMR spectrum of 2-Ga$(CH_2SiMe_3)_3$-1-F-C_6H_4·Li(PMDETA), **6** in C_6D_6.

^7Li NMR (155.5 MHz, C_6D_6 300K): singlets at δ 0.52 and -0.21 ppm, broad resonance at δ 0.09 ppm.

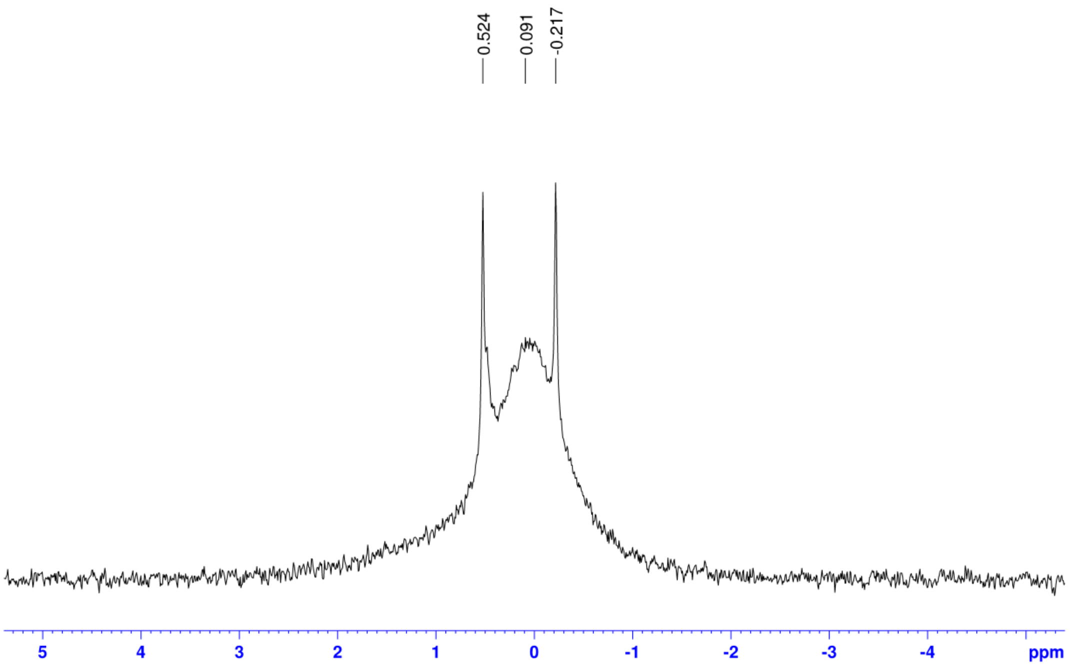

Figure S22 ^7Li NMR spectrum of 2-Ga(CH_2SiMe_3)$_3$-1-F-C_6H_4·Li(PMDETA), **6** in C_6D_6. Spectra indicates a fluxional process (see later)

^{19}F NMR (376.5 MHz, C_6D_6 300K): δ -111.3 ppm, singlet.

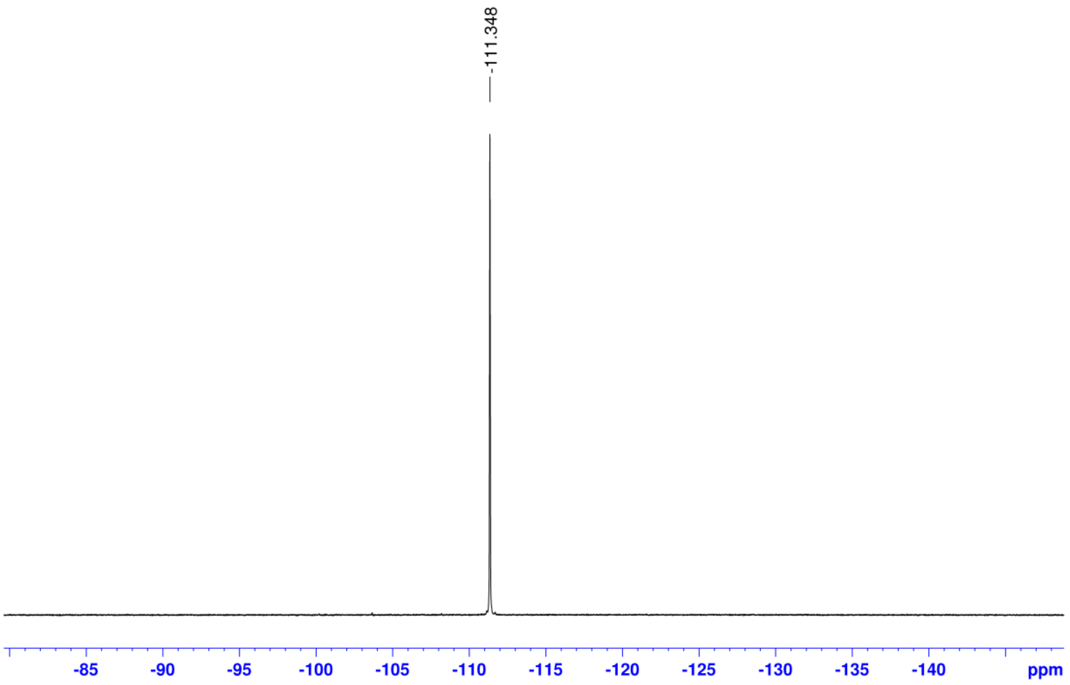

Figure S23 ^{19}F NMR spectrum of 2-Ga(CH_2SiMe_3)$_3$-1-F-C_6H_4·Li(PMDETA), **6** in C_6D_6.

Characterisation of 2-Ga(CH_2SiMe_3)$_3$-1-F-C_6H_4·Li(PMDETA), 6 in d_8-THF

^{1}H NMR (400.1 MHz, d_8-THF 300K): δ 7.94 (1H, m, Ar*H*), 6.77 (1H, m, Ar*H*), 7.00 (1H, m, Ar*H*), 6.49 (1H, td, Ar*H*), 2.46 (4H, m, PMDETA C*H*$_2$), 2.45 (4H, m, PMDETA C*H*$_2$), 2.24 (3H, s, PMDETA *Me*), 2.18 (12H, s, PMDETA *Me*), -0.18 (27H, s, -CH_2Si*Me*$_3$) and -0.83 ppm (6H, s, -C*H*$_2$SiMe$_3$).

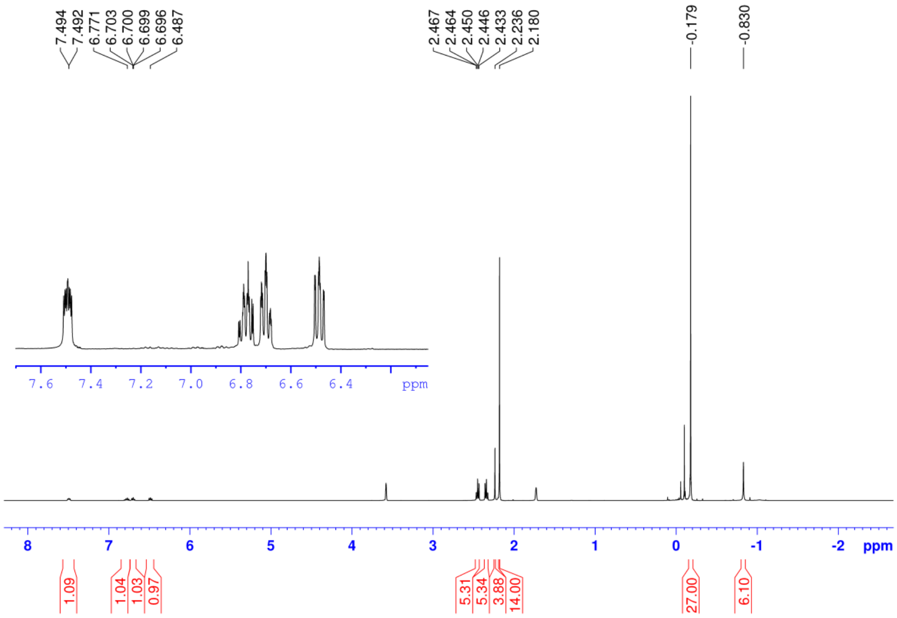

Figure S24 ^{1}H NMR spectrum of 2-Ga(CH_2SiMe_3)$_3$-1-F-C_6H_4·Li(PMDETA), **6** in d_8-THF.

^{13}C NMR (100.6 MHz, d_8-THF 300K) δ 140.0 (d, Ar*C*-H, $^3J_{C\text{-}F}$ 22.5 Hz), 125.2 (d, Ar*C*H, $^3J_{C\text{-}F}$ 6.4 Hz), 121.9 (s, Ar*C*H), 112.2 (d, Ar*C*H, $^2J_{C\text{-}F}$ 33.1 Hz), 58.5 (s, PMDETA *C*H$_2$), 56.6 (s, PMDETA *C*H$_2$), 45.9 (s, PMDETA *Me*), 44.3 (s, PMDETA *Me*), 3.9 (s, -*C*H$_2$SiMe$_3$) and 3.5 ppm (s, -CH$_2$Si*Me*$_3$).

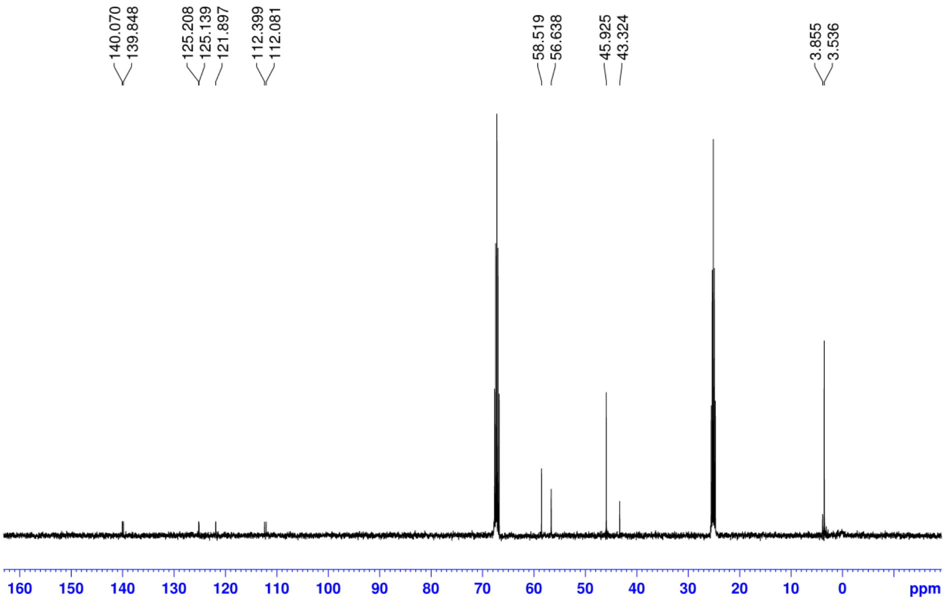

Figure S25 ^{13}C NMR spectrum of 2-Ga(CH_2SiMe_3)$_3$-1-F-C_6H_4·Li(PMDETA), **6** in d_8-THF.

^{7}Li NMR (155.5 MHz, C_6D_6 300K): singlets at δ -0.27 ppm

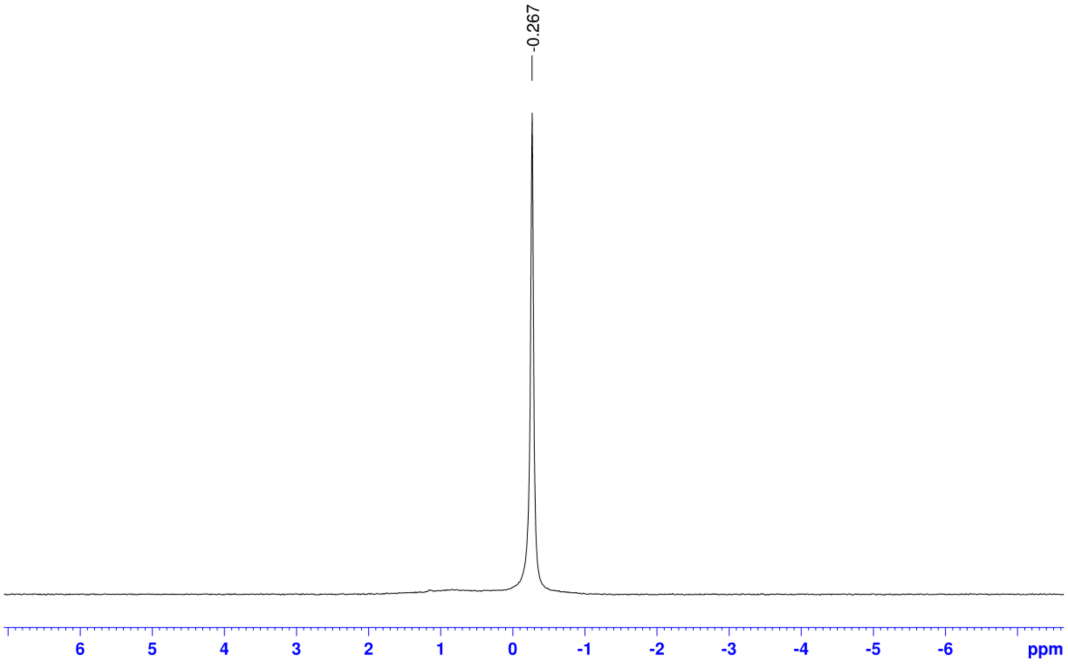

Figure S26 ^{7}Li NMR spectrum of 2-Ga(CH_2SiMe_3)$_3$-1-F-C_6H_4·Li(PMDETA), **6** in d_8-THF.

^{19}F NMR (376.5 MHz, d_8-THF 300K): δ -93.1 ppm, singlet.

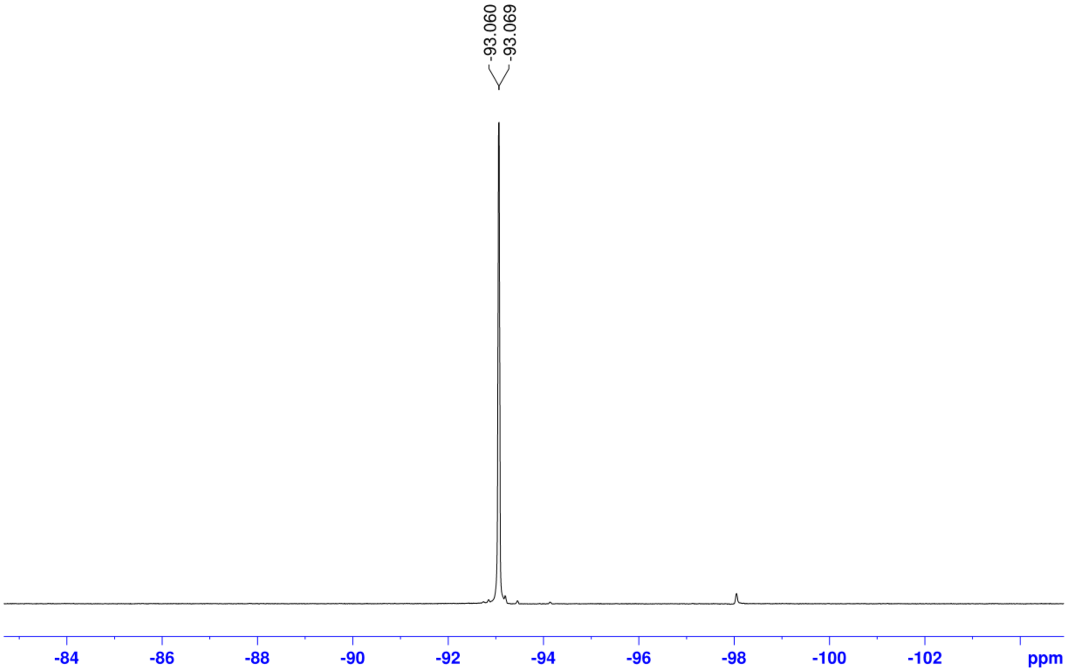

Figure S27 ^{19}F NMR spectrum of 2-Ga(CH_2SiMe_3)$_3$-1-F-C_6H_4·Li(PMDETA), **6** in d_8-THF.

Variable temperature ^{7}Li nmr study of (2-Ga(CH_2SiMe_3)$_3$-1,3,4,5-F_4-C_6H_1·Li(PMDETA), **9**

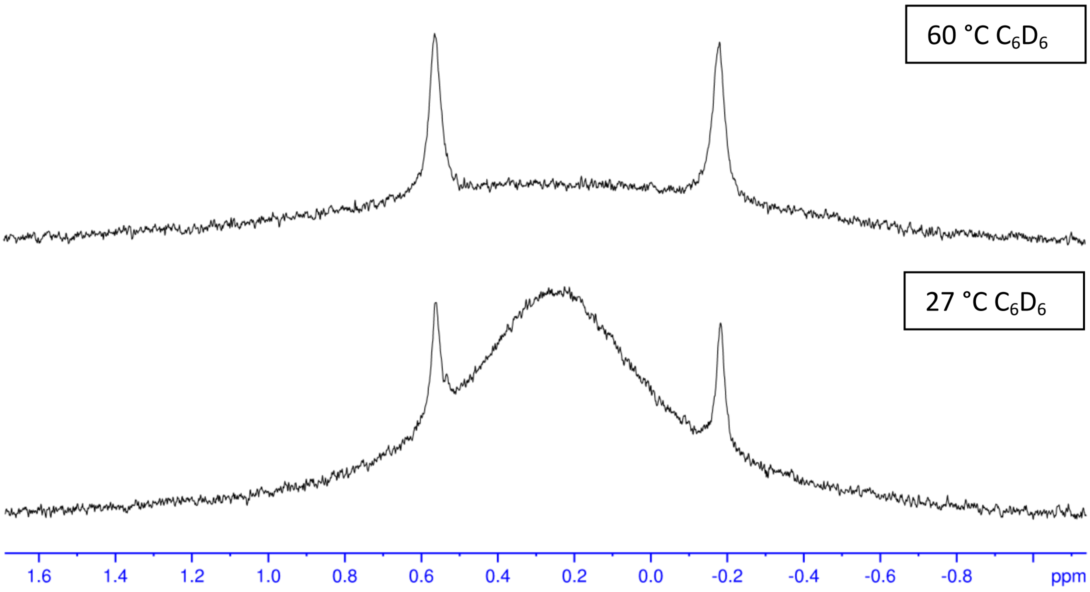

Figure S28 ^{7}Li NMR spectra of (2-Ga(CH_2SiMe_3)$_3$-1,3,4,5-F_4-C_6H_1·Li(PMDETA), **9** in C_6D_6 at 27 and 60 °C.

Characterisation of 2-Ga(CH_2SiMe_3)$_3$-1,3-F_2-C_6H_3·Li(PMDETA), 7

Elemental analysis (%) calculated for $C_{27}H_{59}Ga_1F_2Li_1N_3Si_3$: C 51.91, H 9.52, N 6.73; found: C 51.55, H 9.69, N 7.05.

^{1}H NMR (400.1 MHz, C_6D_6 300K): δ 6.92 (1H, apparent quintet, Ar*H*), 6.56 (2H, dd, Ar*H*), 1.91 (3H, br. s, PMDETA *Me*), 1.79 (12H, br. s, PMDETA *Me*), 1.64 (8H, br. s, PMDETA C*H*$_2$), 0.43 (27H, s, -CH_2Si*Me*$_3$) and -0.18 ppm (6H, s, -C*H*$_2$SiMe$_3$).

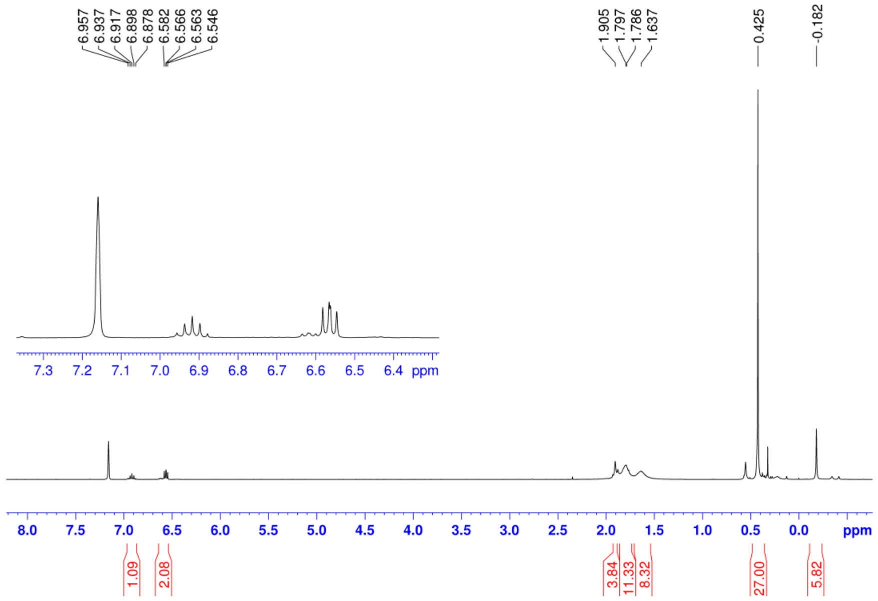

Figure S29 ^{1}H NMR spectrum of 2-Ga(CH_2SiMe_3)$_3$-1,3-F_2-C_6H_3·Li(PMDETA), **7** in C_6D_6. Minor resonance in the aromatic region corresponds to minor conformer of **7**.

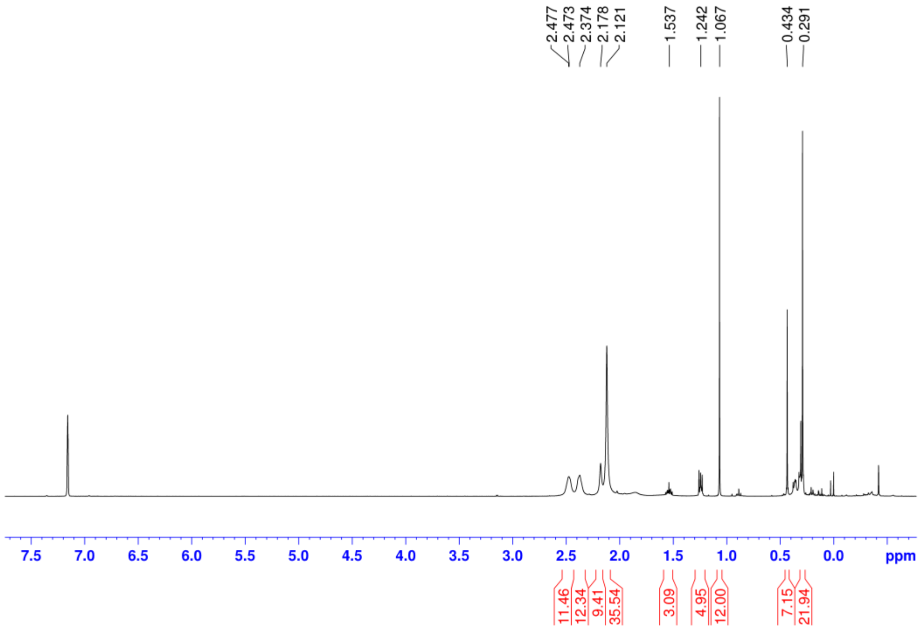

Figure S30 ^{1}H NMR spectrum of 2-Ga(CH_2SiMe_3)$_3$-1,3-F_2-C_6H_3·Li(PMDETA), **7** reaction filtrate in C_6D_6. Resonances corresponding to PMDETA, TMPH and Ga(CH_2Me_3)$_3$ are present. Crucially no sign of decomposition (autometallation as is the case with **1**) is detected.

^{13}C NMR (100.6 MHz, C_6D_6 300K) δ 170.5 (dd, Ar*C*-F, $^1J_{C\text{-}F}$ 222.8 Hz, Ar*C*-F, $^3J_{C\text{-}F}$ 30.7 Hz), 127.32 (t, Ar*C*-H, $^3J_{C\text{-}F}$ 9.5 Hz), 109.7 (dd, Ar*C*H, $^2J_{C\text{-}F}$ 33.9 Hz, Ar*C*-F, $^4J_{C\text{-}F}$ 2.7 Hz), 56.7 (s, PMDETA *C*H_2), 53.0 (s, PMDETA *C*H_2), 45.2 (s, PMDETA *Me*), 44.4 (s, PMDETA *Me*), 3.9 (s, -CH_2Si*Me*$_3$) and 2.1 ppm (s, -*C*H_2SiMe_3).

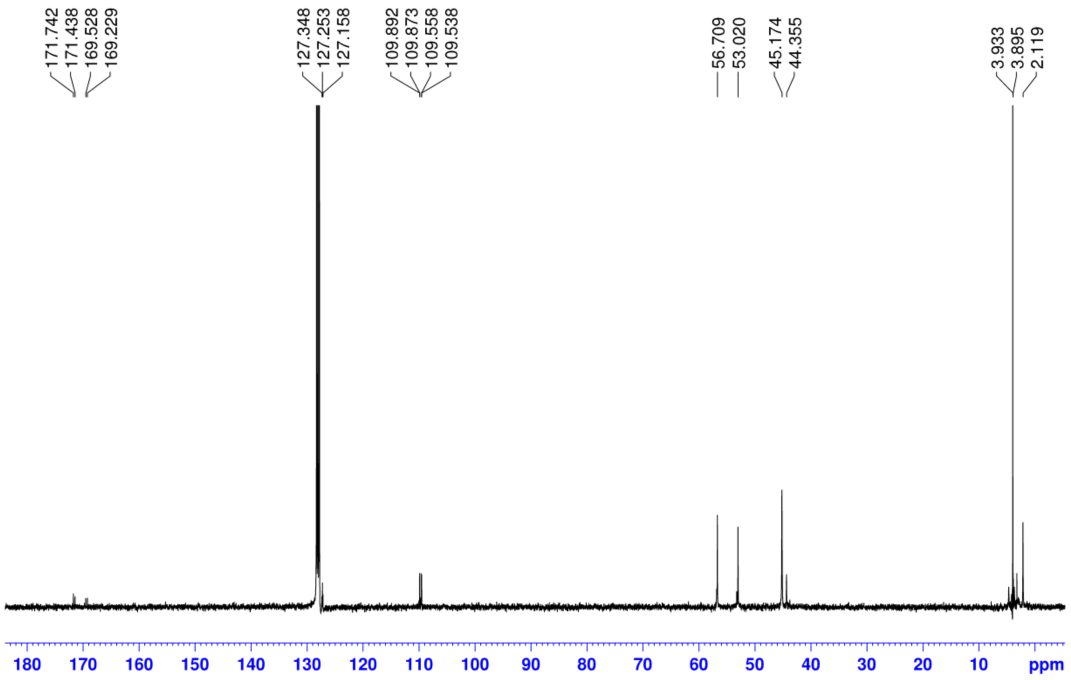

Figure S31 ^{13}C NMR spectrum of 2-Ga(CH_2SiMe_3)$_3$-1,3-F_2-C_6H_3·Li(PMDETA), **7** in C_6D_6.

^7Li NMR (155.5 MHz, C_6D_6 300K): singlets at δ 0.56 and -0.19 ppm.

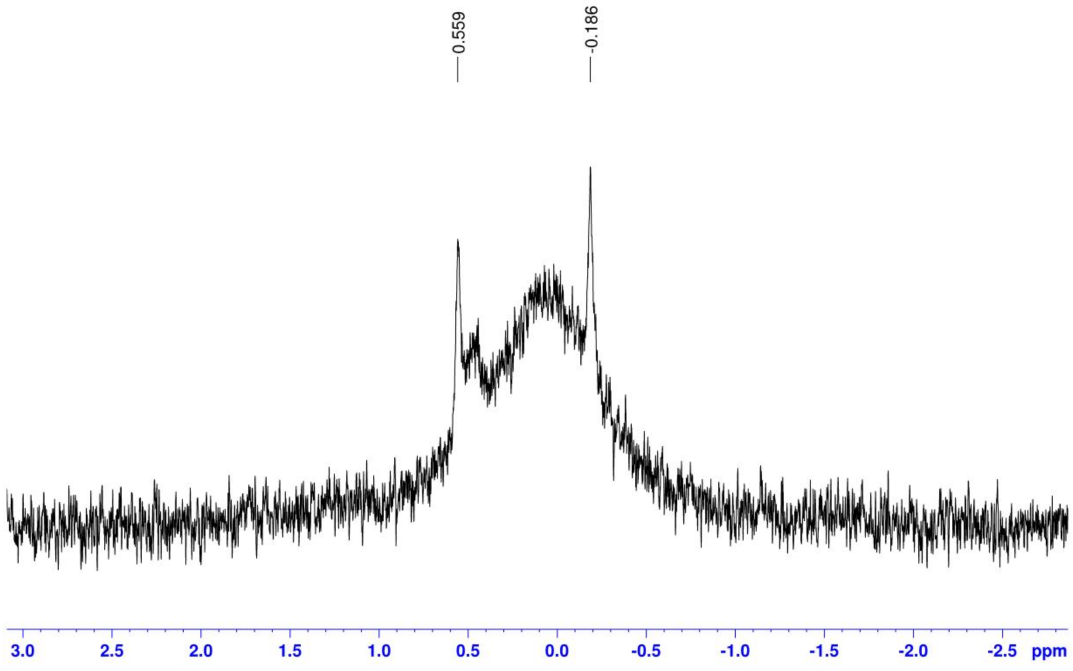

Figure S32 ^7Li NMR spectrum of 2-Ga(CH_2SiMe_3)$_3$-1,3-F_2-C_6H_3·Li(PMDETA), **7** in C_6D_6.

^{19}F NMR (376.5 MHz, C_6D_6 300K): δ -94.6 and -95.7 ppm, singlets.

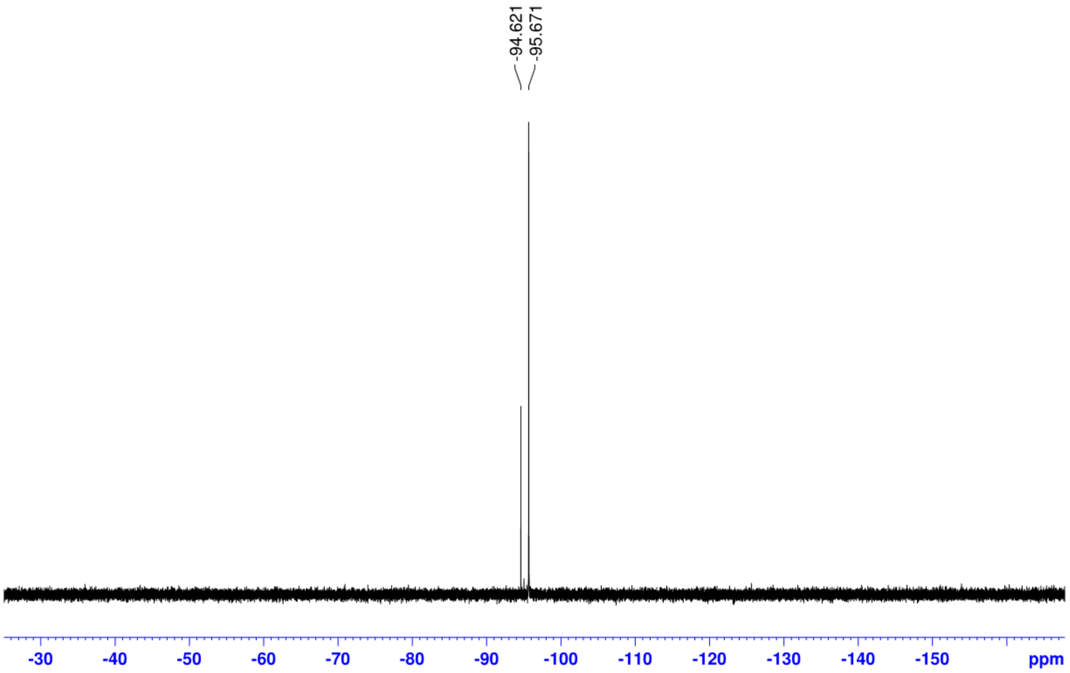

Figure S33 ^{19}F NMR spectrum of 2-Ga(CH_2SiMe_3)$_3$-1,3-F_2-C_6H_3·Li(PMDETA), **7** in C_6D_6. Two resonances correspond to the two isomeric compounds present in the reaction mixture.

Characterisation of 2-Ga(CH_2SiMe_3)$_3$-1,3-F_2-C_6H_3·Li(PMDETA), 7 in d_8-THF: Demonstrates that in the polar solvent, a SSIP arrangement is obtained, as indicated most clearly in the ^{7}Li and ^{19}F spectra.

^{1}H NMR (400.1 MHz, d_8-THF 300K): δ 6.78 (1H, apparent quintet, Ar*H*), 6.36 (2H, dd, Ar*H*), 2.45 (4H, m, PMDETA C*H*$_2$), 2.35 (4H, m, PMDETA C*H*$_2$), 2.24 (3H, s, PMDETA *Me*), 2.19 (12H, s, PMDETA *Me*), -0.17 (27H, s, -CH_2Si*Me*$_3$) and -0.75 ppm (6H, s, -C*H*$_2$SiMe$_3$).

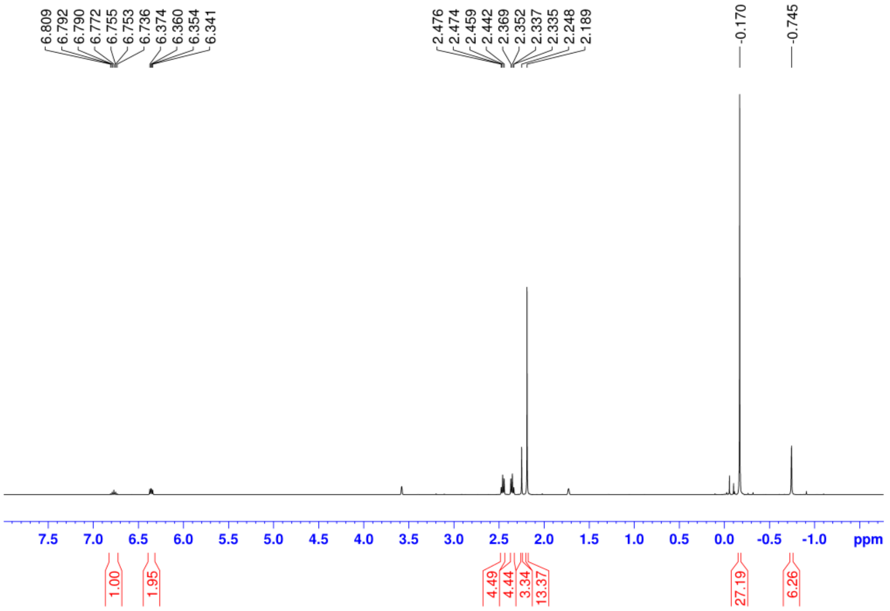

Figure S34 ^{1}H NMR spectrum of 2-Ga(CH_2SiMe_3)$_3$-1,3-F_2-C_6H_3·Li(PMDETA), **7** in d_8-THF.

^{13}C NMR (100.6 MHz, d_8-THF 300K) δ 170.5 (dd, Ar*C*-F, $^1J_{C-F}$ 231.7 Hz, Ar*C*-F, $^3J_{C-F}$ 31.7 Hz), 126.0 (t, Ar*C*-H, $^3J_{C-F}$ 10.5 Hz), 108.9 (dd, Ar*C*H, $^2J_{C-F}$ 33.5 Hz, Ar*C*-F, $^4J_{C-F}$ 3.8 Hz), 58.5 (s, PMDETA *C*H$_2$), 56.5 (s, PMDETA *C*H$_2$), 45.9 (s, PMDETA *Me*), 43.4 (s, PMDETA *Me*), 3.4 (s, -CH_2Si*Me*$_3$) and 1.2 ppm (s, -*C*H$_2$SiMe$_3$).

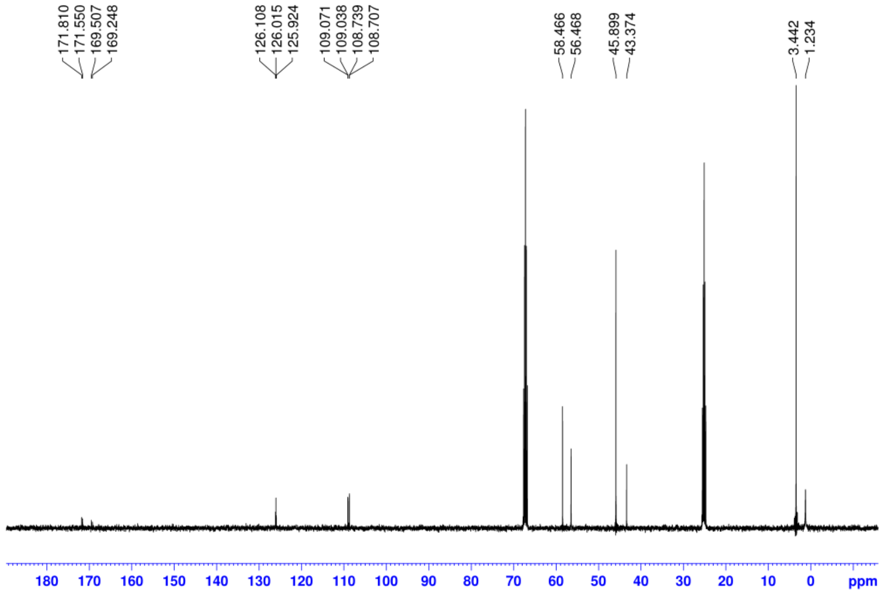

Figure S35 ^{13}C NMR spectrum of 2-Ga(CH_2SiMe_3)$_3$-1,3-F_2-C_6H_3·Li(PMDETA), **7** in d_8-THF.

^{7}Li NMR (155.5 MHz, C_6D_6 300K): singlet at δ -0.23 ppm

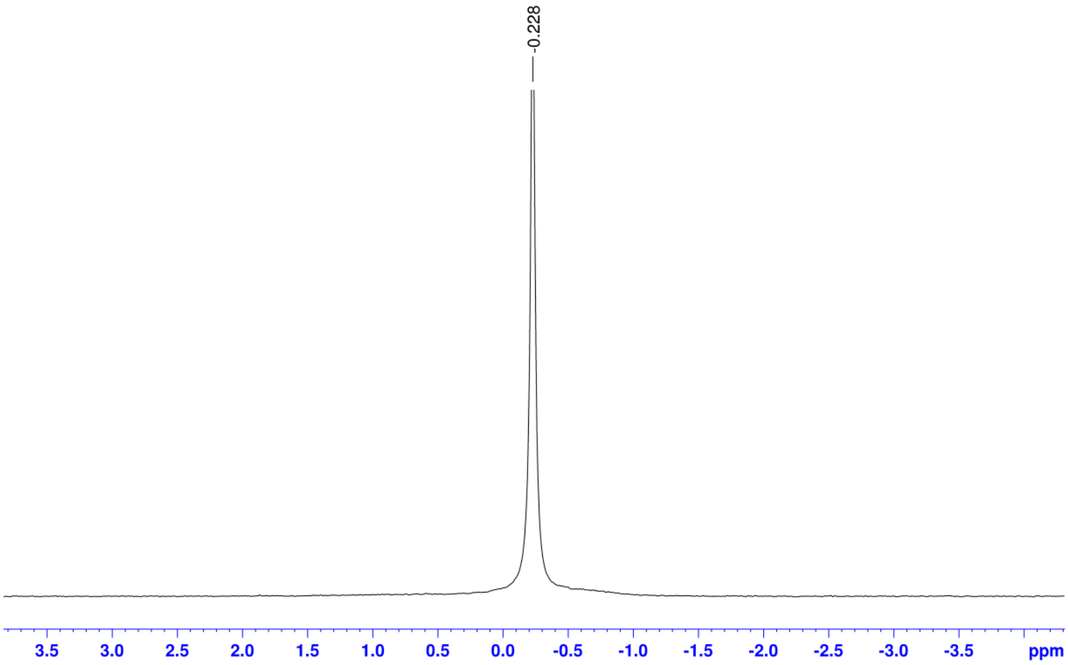

Figure S36 ^{7}Li NMR spectrum of 2-Ga(CH_2SiMe_3)$_3$-1,3-F_2-C_6H_3·Li(PMDETA), **7** in d_8-THF.

^{19}F NMR (376.5 MHz, d_8-THF 300K): δ -89.7 ppm.

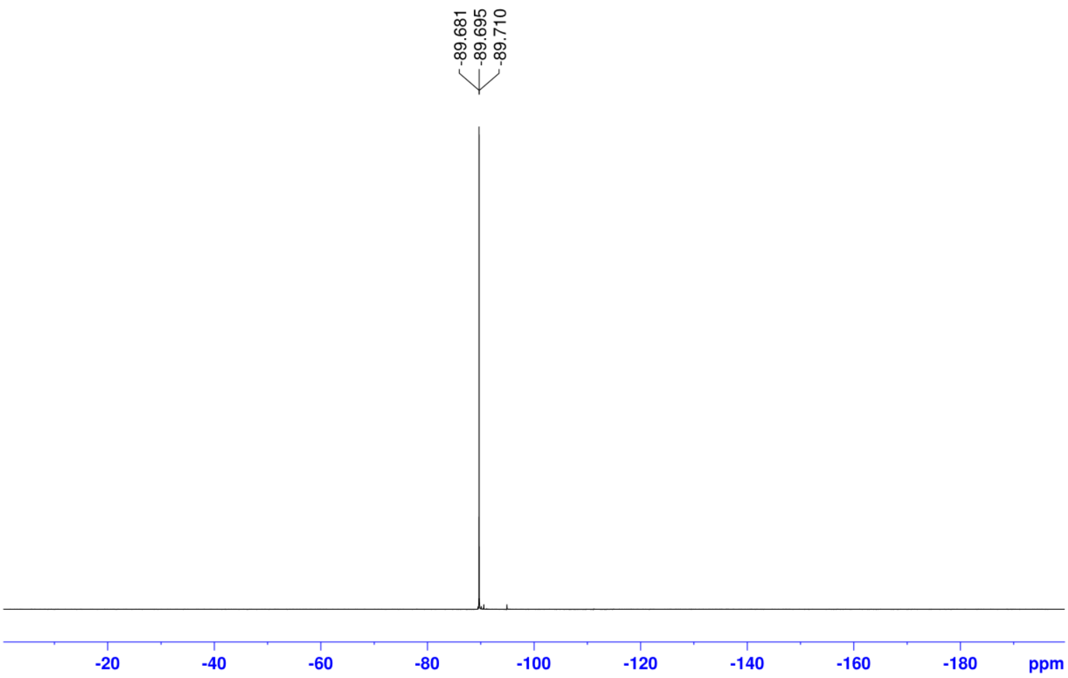

Figure S37 ^{19}F NMR spectrum of 2-Ga(CH_2SiMe_3)$_3$-1,3-F_2-C_6H_3·Li(PMDETA), **7** in d_8-THF.

Characterisation of (2-Ga(CH_2SiMe_3)$_3$-1,3,5-F_3-C_6H_2·Li(PMDETA), 8

Elemental analysis (%) calculated for $C_{27}H_{58}Ga_1F_3Li_1N_3Si_3$: C 50.46, H 9.10, N 6.54; found: C 49.95, H 9.60, N 7.28.

^{1}H NMR (400.1 MHz, C_6D_6 300K): δ 6.43 (2H, m, Ar*H*), 1.92 (3H, br. s, PMDETA *Me*), 1.86 (12H,br. s, PMDETA *Me*), 1.67 (8H, br. s, PMDETA *CH$_2$*), 0.42 (27H, s, -CH_2Si*Me$_3$*) and -0.20 ppm (6H, s, -*CH$_2$*$SiMe_3$).

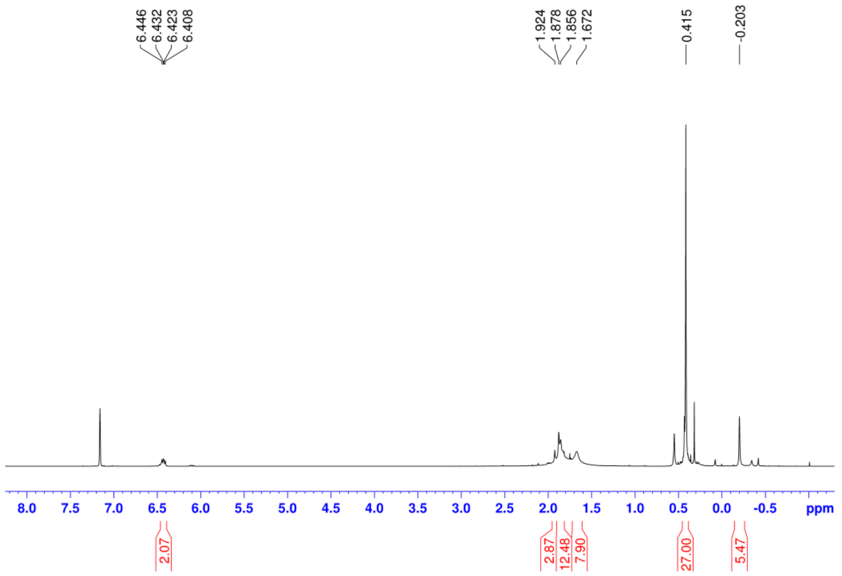

Figure S38 ^{1}H NMR spectrum of (2-Ga(CH_2SiMe_3)$_3$-1,3,5-F_3-C_6H_2·Li(PMDETA), **8** in C_6D_6.

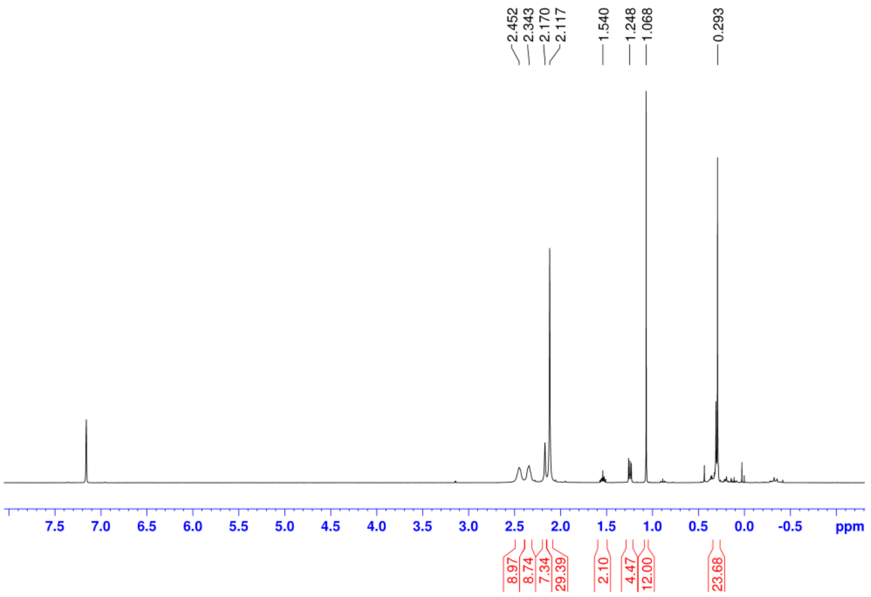

Figure S39 ^{1}H NMR spectrum of (2-Ga(CH_2SiMe_3)$_3$-1,3,5-F_3-C_6H_2·Li(PMDETA), **8** reaction filtrate in C_6D_6. Resonances corresponding to PMDETA, TMPH and Ga(CH_2Me_3)$_3$ are present. No sign of decomposition (autometallation as is the case with **1**) is detected.

^{13}C NMR (100.6 MHz, C_6D_6, 300K) δ 98.2 (qd, Ar*C*H, $^{2}J_{C\text{-}F}$ 22.9 Hz, $^{4}J_{C\text{-}F}$ 4.5 Hz), 56.7 (s, PMDETA *C*H_2), 53.0 (s, PMDETA *C*H_2), 45.1 (s, PMDETA *Me*), 44.2 (s, PMDETA *Me*), 3.9 (s, -CH_2Si*Me*$_3$) and 2.0 ppm (s, -*C*H_2SiMe_3).

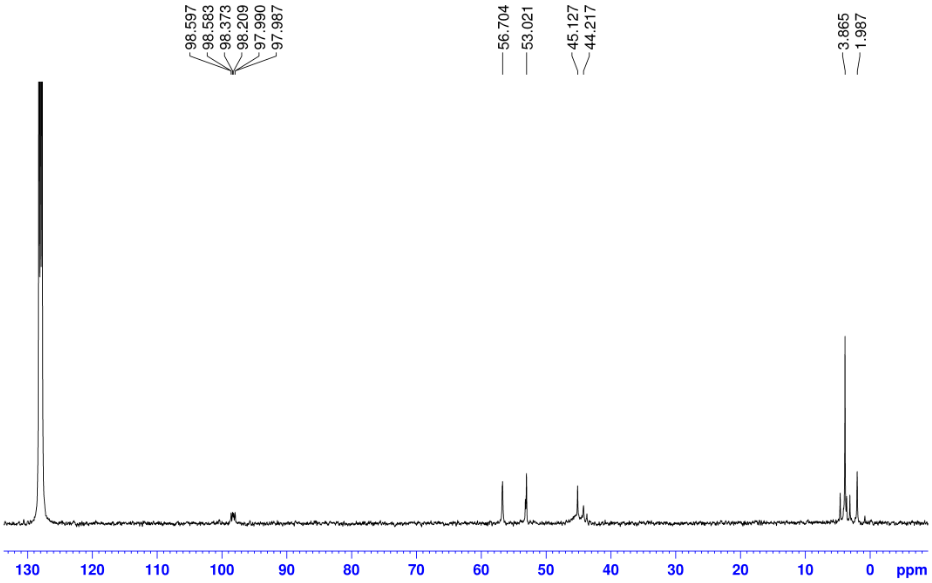

Figure S40 ^{13}C NMR spectrum of (2-Ga(CH_2SiMe_3)$_3$-1,3,5-F_3-C_6H_2·Li(PMDETA), **8** in C_6D_6.

^{7}Li NMR (155.5 MHz, C_6D_6 300K): singlets at δ 0.56 and -0.18 ppm.

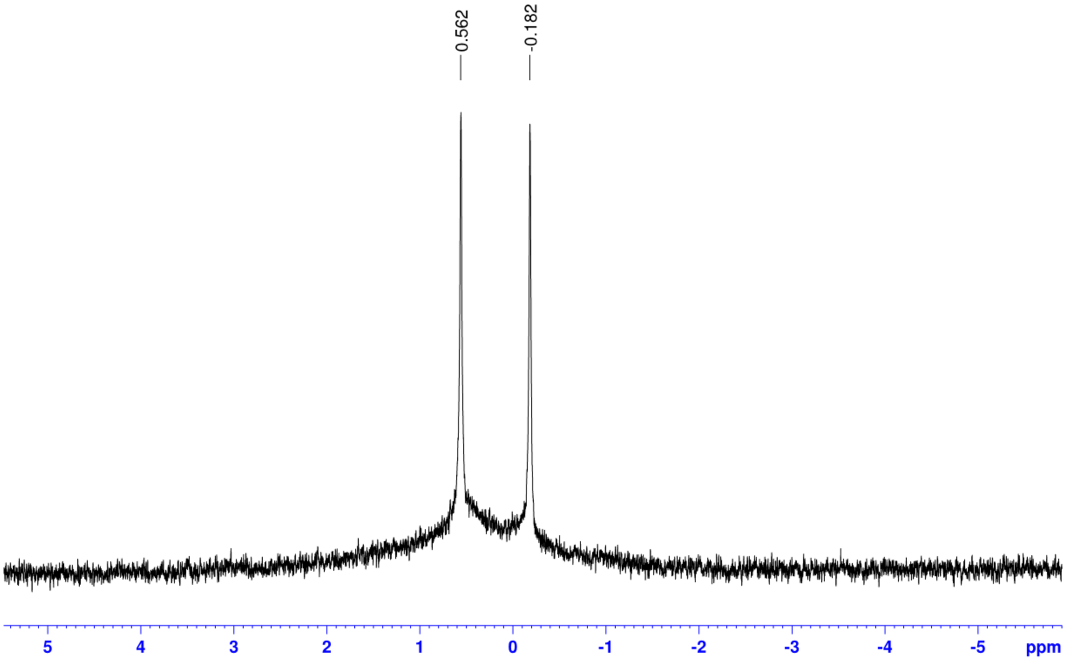

Figure S41 ^{7}Li NMR spectrum of (2-Ga(CH_2SiMe_3)$_3$-1,3,5-F_3-C_6H_2·Li(PMDETA), **8** in C_6D_6. Two singlets in agreement with two CIP isomers present in solution.

^{19}F NMR (376.5 MHz, C_6D_6 300K): δ -92.2 and -116.8 ppm (major conformer), -91.5 and -116.2 ppm (minor conformer).

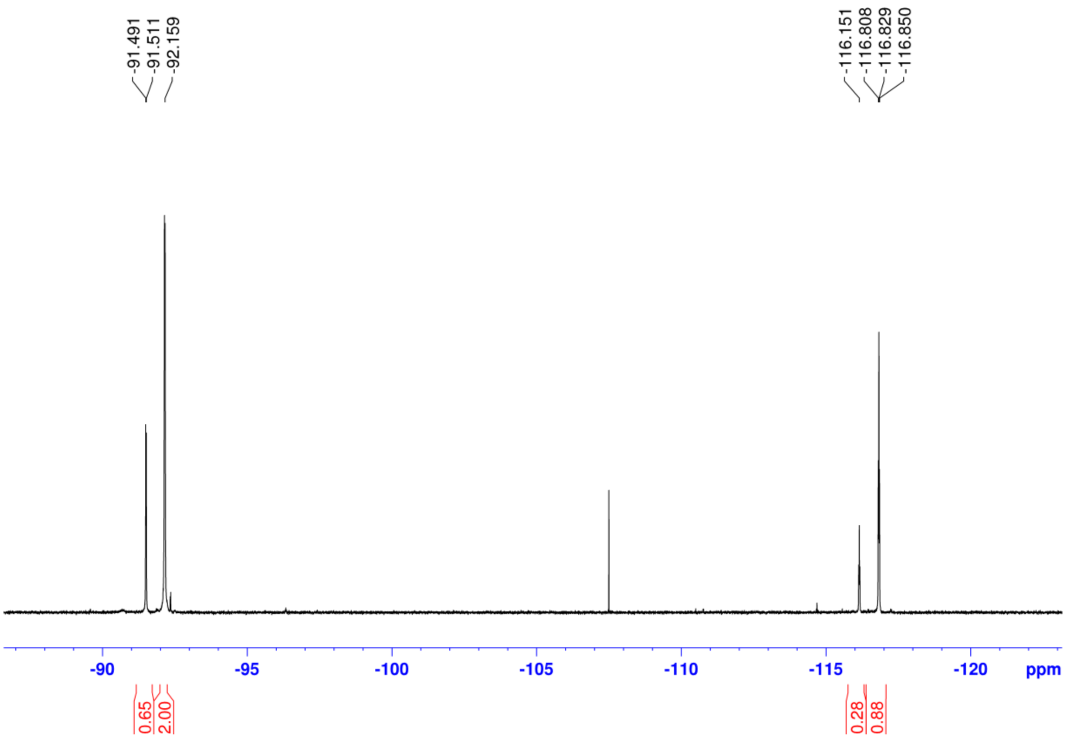

Figure S42 ^{19}F NMR spectrum of (2-Ga(CH_2SiMe_3)$_3$-1,3,5-F_3-C_6H_2·Li(PMDETA), **8** in C_6D_6. Minor peaks are consistent with a second conformer present in solution. Resonance at -107 corresponds to small amount of hydrolysis in solution.

Characterisation of (2-Ga(CH_2SiMe_3)$_3$-1,3,5-F_3-C_6H_2·Li(PMDETA), 8 in *d*$_8$-THF: Demonstrates that in the polar solvent, a SSIP arrangement is obtained, as indicated most clearly in the ^{7}Li and ^{19}F spectra.

^{1}H NMR (400.1 MHz, *d*$_8$-THF 300K): δ 6.17 (2H, m, Ar*H*), 6.77 (1H, m, Ar*H*), 2.44 (4H, m, PMDETA C*H*$_2$), 2.33 (4H, m, PMDETA C*H*$_2$), 2.22 (3H, s, PMDETA *Me*), 2.17 (12H, s, PMDETA *Me*), -0.17 (27H, s, -CH_2Si*Me*$_3$) and -0.25 ppm (6H, s, -C*H*$_2$SiMe$_3$).

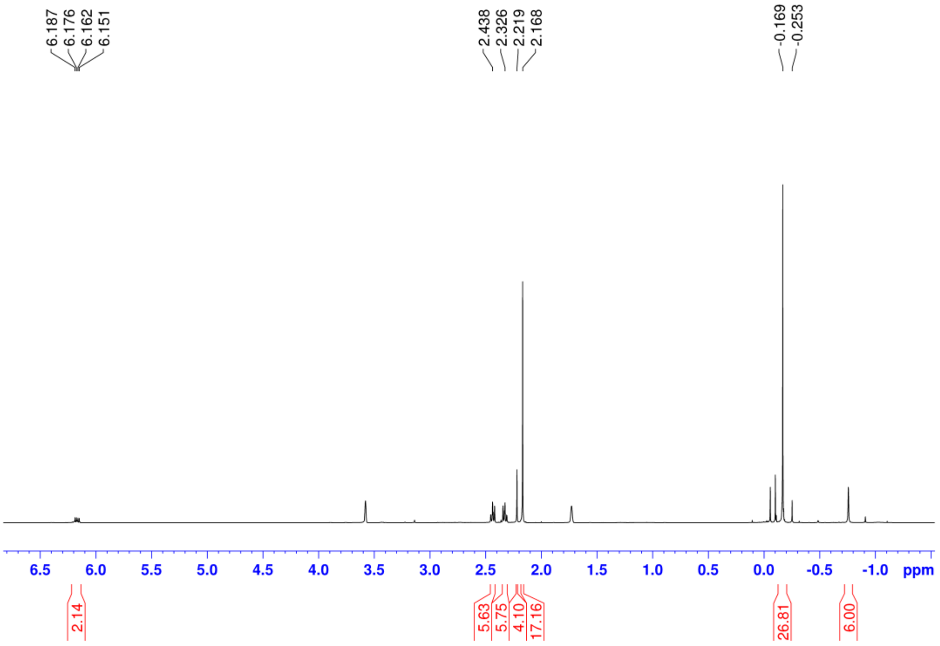

Figure S43 ^{1}H NMR spectrum of (2-Ga(CH_2SiMe_3)$_3$-1,3,5-F_3-C_6H_2·Li(PMDETA), **8** in *d*$_8$-THF.

^{13}C NMR (100.6 MHz, *d*$_8$-THF 300K) δ 97.2 (qd, Ar*C*-H, $^2J_{C\text{-}F}$ 23.5 Hz $^4J_{C\text{-}F}$ 5.2 Hz), 58.6 (s, PMDETA *C*H$_2$), 56.9 (s, PMDETA *C*H$_2$), 46.0 (s, PMDETA *Me*), 44.2 (s, PMDETA *Me*), 3.4 (s, -CH_2Si*Me*$_3$) and 1.1 ppm (s, -*C*H$_2$SiMe$_3$).

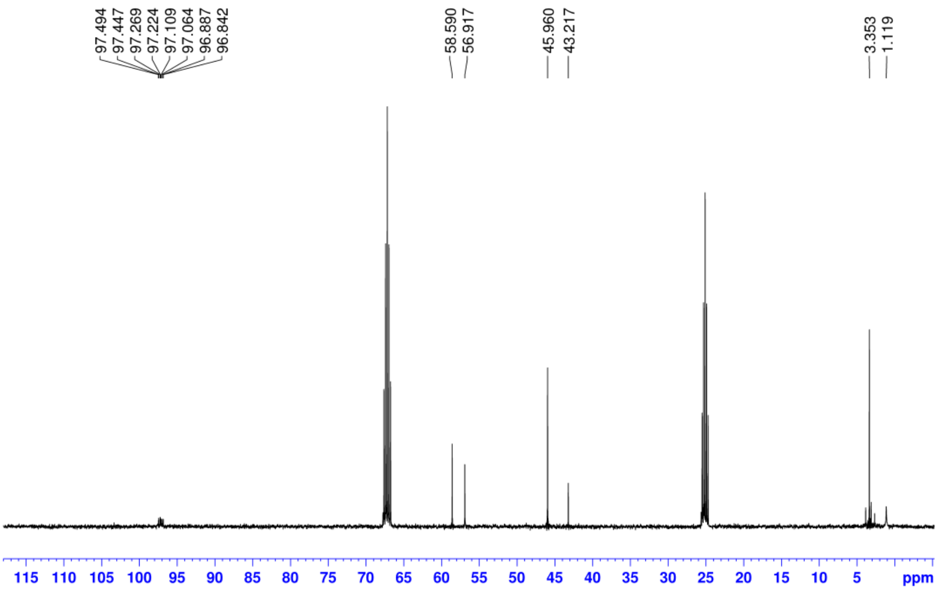

Figure S44 ^{13}C NMR spectrum of (2-Ga(CH_2SiMe_3)$_3$-1,3,5-F_3-C_6H_2·Li(PMDETA), **8** in *d*$_8$-THF.

^{7}Li NMR (155.5 MHz, C_6D_6 300K): singlet at δ -1.62 ppm

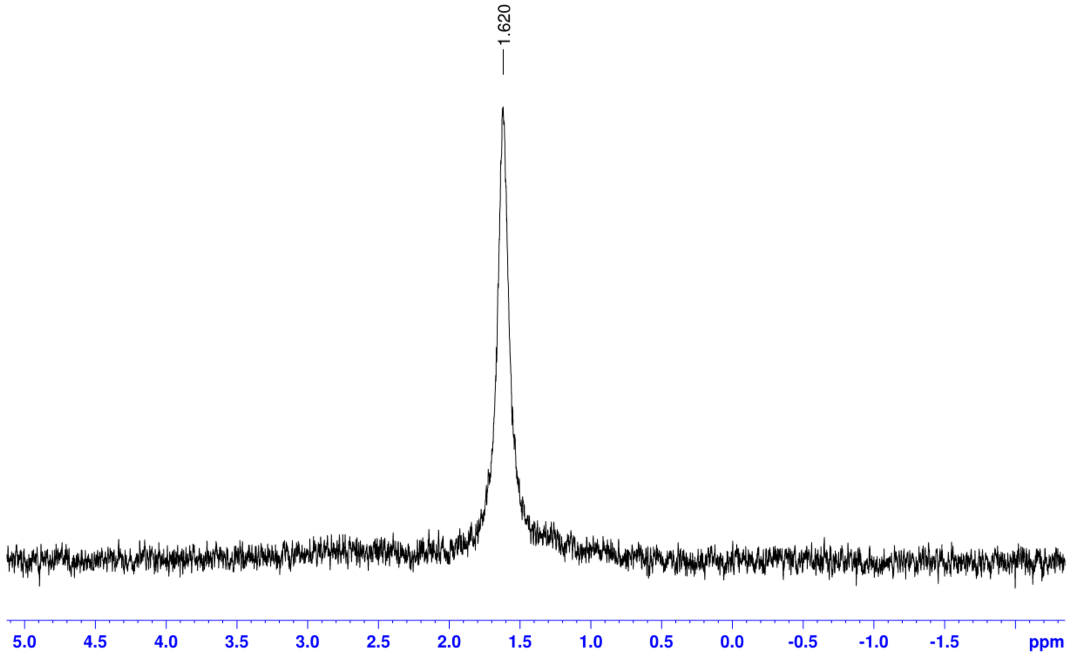

Figure S45 ^{7}Li NMR spectrum of (2-Ga(CH_2SiMe_3)$_3$-1,3,5-F_3-C_6H_2·Li(PMDETA), **8** in d_8-THF.

^{19}F NMR (376.5 MHz, d_8-THF 300K): δ -85.3 and -118.7 ppm.

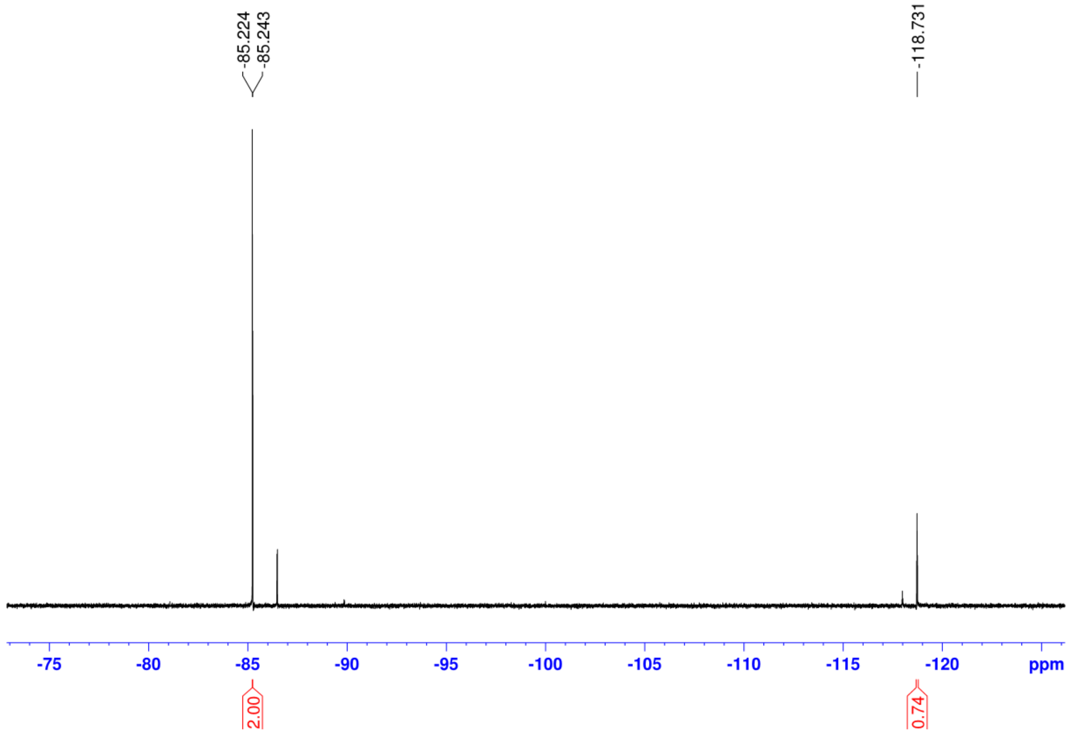

Figure S46 ^{19}F NMR spectrum of (2-Ga(CH_2SiMe_3)$_3$-1,3,5-F_3-C_6H_2·Li(PMDETA), **8** in d_8-THF.

Characterisation of (2-Ga(CH_2SiMe_3)$_3$-1,3,4,5-F_4-C_6H_1·Li(PMDETA) 9

Elemental analysis (%) calculated for $C_{27}H_{57}Ga_1F_4Li_1N_3Si_3$: C 49.09, H 8.70, N 6.36; found: C 48.75, H 8.70, N 6.36.

^{1}H NMR (400.1 MHz, C_6D_6 300K): δ 6.49 (1H, m, Ar*H*), 1.96 (3H, s, PMDETA *Me*), 1.73 (20H,br. s, PMDETA *Me* and C*H*$_2$), 0.37 (27H, s, -CH_2Si*Me*$_3$) and -0.15 ppm (6H, s, -C*H*$_2$SiMe$_3$).

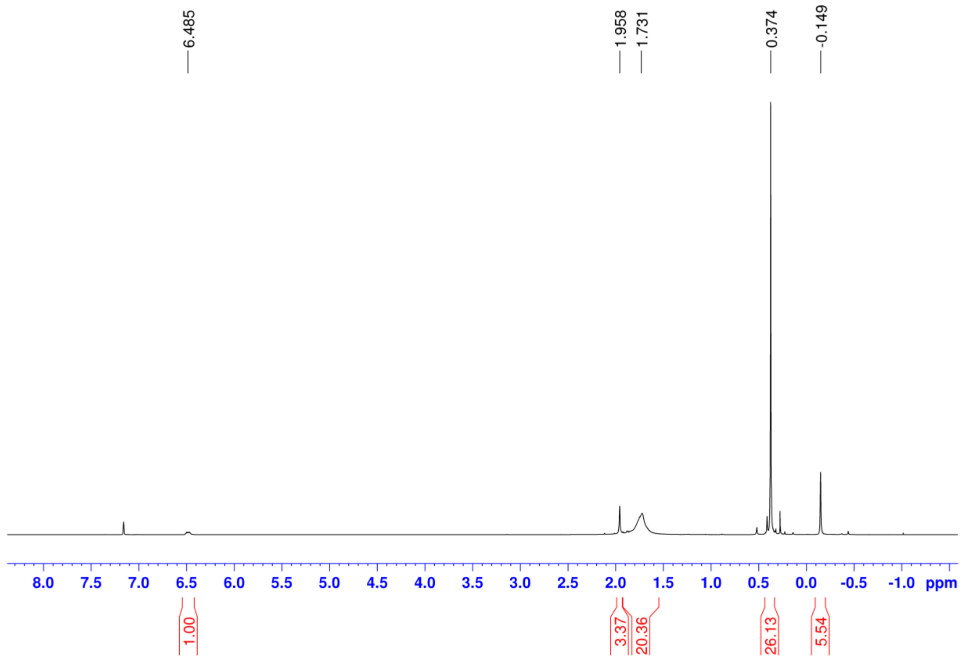

Figure S47 ^{1}H NMR spectrum of (2-Ga(CH_2SiMe_3)$_3$-1,3,4,5-F_4-C_6H_1·Li(PMDETA), **9** in C_6D_6.

^{13}C NMR (100.6 MHz, C_6D_6 300K) δ 100.8 (dd, Ar*C*-H, $^2J_{C\text{-}F}$ 40.6 Hz, $^3J_{C\text{-}F}$ 18.1 Hz), 56.5 (s, PMDETA *C*H$_2$), 52.9 (s, PMDETA *C*H$_2$), 44.6 (s, PMDETA *Me*), 43.9 (s, PMDETA *Me*), 3.7 (s, -CH_2Si*Me*$_3$) and 1.9 ppm (s, -*C*H$_2$SiMe$_3$).

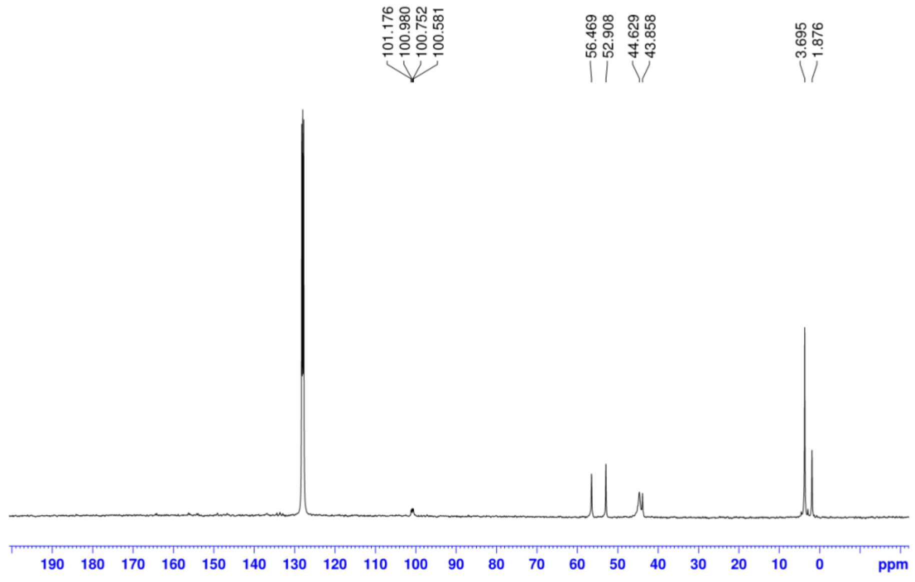

Figure S48 ^{13}C NMR spectrum of (2-Ga(CH_2SiMe_3)$_3$-1,3,4,5-F_4-C_6H_1·Li(PMDETA), **9** in C_6D_6.

^{7}Li NMR (155.5 MHz, C_6D_6 300K): singlets at δ 0.56 and -0.18 ppm, broad resonance between.

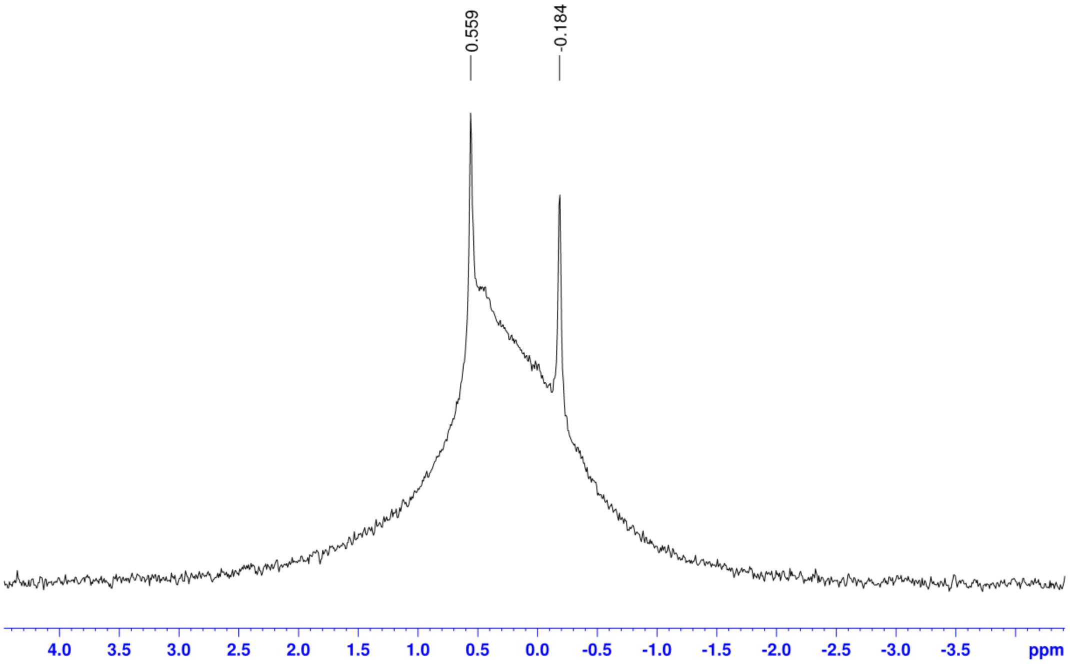

Figure S49 ^{7}Li NMR spectrum of (2-Ga(CH_2SiMe_3)$_3$-1,3,4,5-F_4-C_6H_1·Li(PMDETA), **9** in C_6D_6. Spectra indicates a fluxional process between two different conformers in the relatively non coordinating solvent.

^{19}F NMR (376.5 MHz, C_6D_6 300K): δ -88.0, -128.1, -141.3 and -177.6 ppm, singlets.

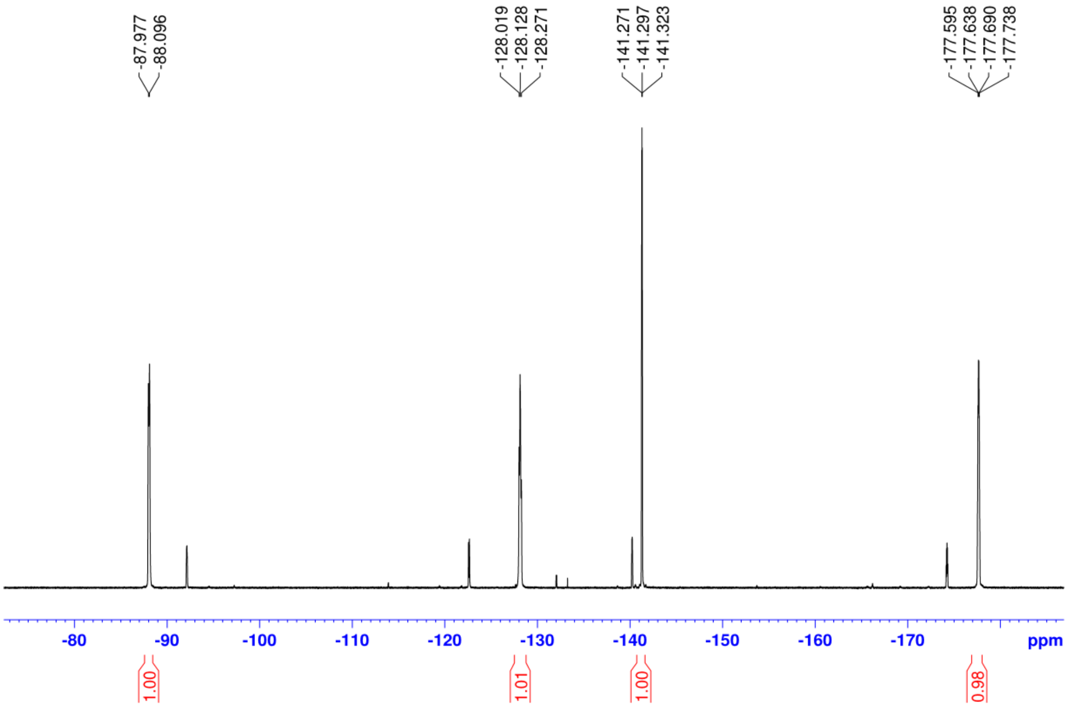

Figure S50 ^{19}F NMR spectrum of (2-Ga(CH_2SiMe_3)$_3$-1,3,4,5-F_4-C_6H_1·Li(PMDETA), **9** in C_6D_6. Minor peaks are consistent with a second conformer present in solution.

Decomposition study of 2-Ga(CH_2SiMe_3)$_3$-1-F-C_6H_4·Li(PMDETA) 6 at room temperature in C_6D_6 in a J. Young nmr tube using ferrocene as an internal standard.

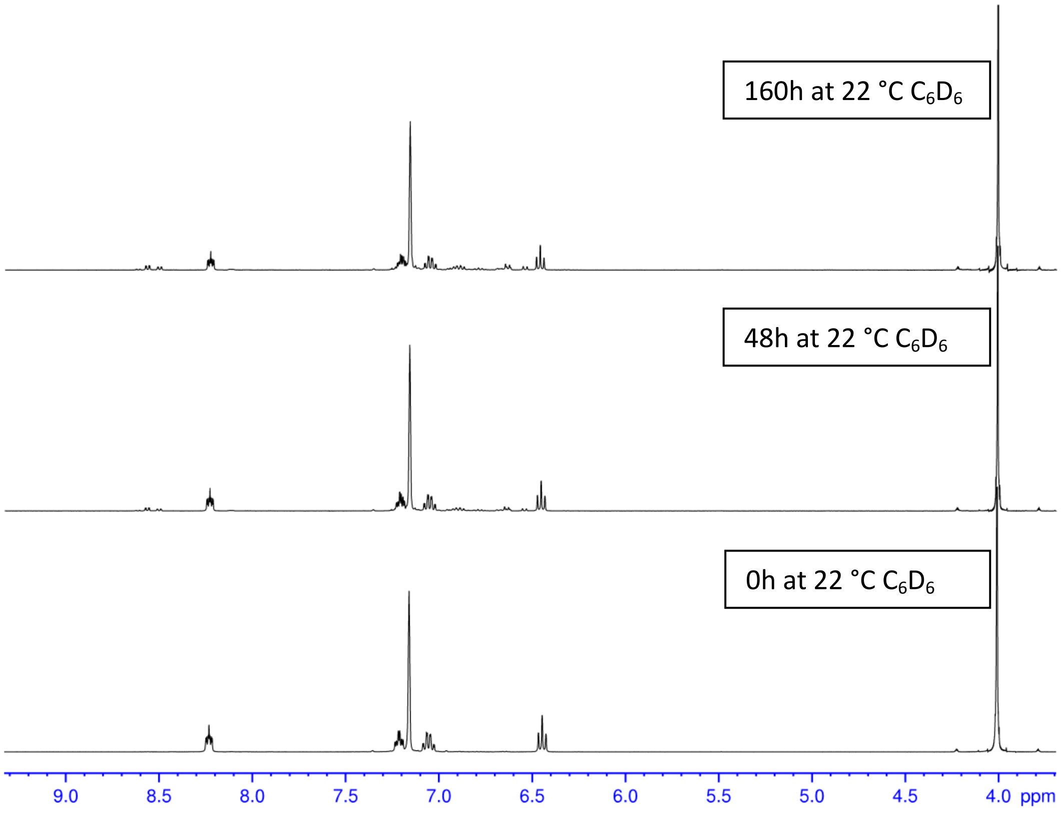

Figure S51 Decomposition of 2-Ga(CH_2SiMe_3)$_3$-1-F-C_6H_4·Li(PMDETA), **6** in C_6D_6, referenced against ferrocene as an internal standard.

Reaction monitoring of 2-Ga(CH_2SiMe_3)$_3$-1,3,5-F_3-C_6H_2·Li(PMDETA), 8 in d_8-THF

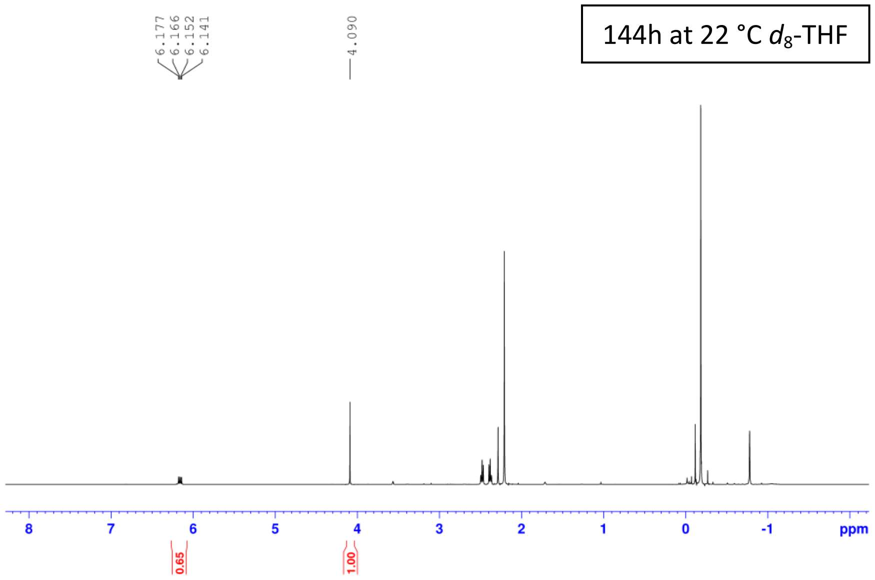

Figure S52 ^{1}H NMR spectrum showing essentially no decomposition of 2-Ga(CH_2SiMe_3)$_3$-1,3,5-F_3-C_6H_2·Li(PMDETA), **8** in d_8-THF after 144 h, referenced against ferrocene as an internal standard.

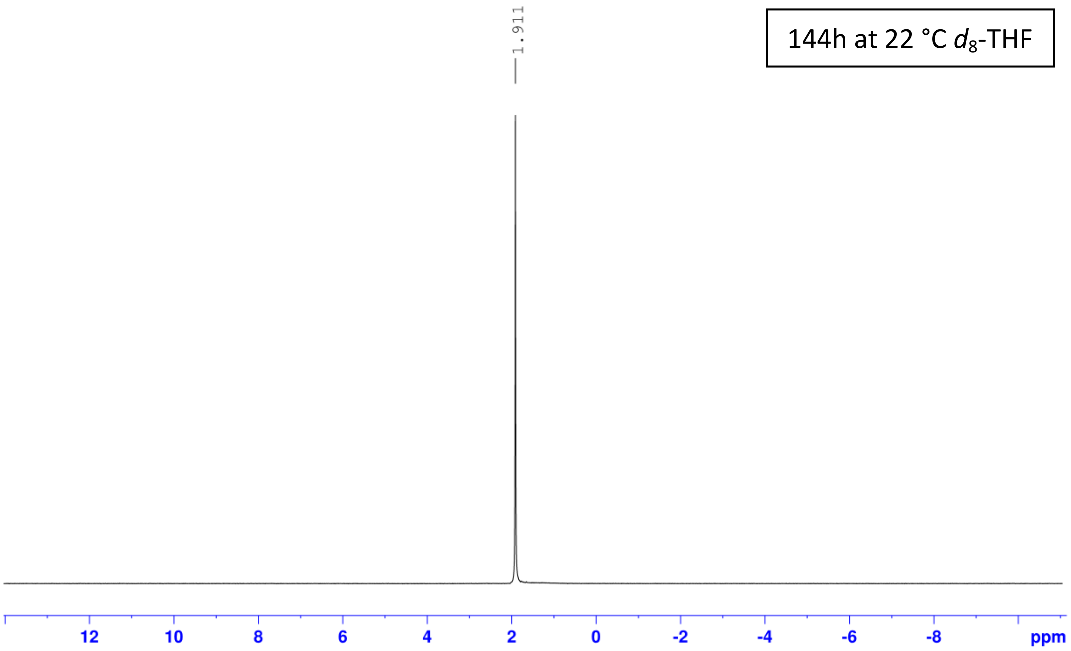

Figure S53 ^{7}Li NMR spectrum showing essentially no decomposition of 2-Ga(CH_2SiMe_3)$_3$-1,3,5-F_3-C_6H_2·Li(PMDETA), **8** in d_8-THF after 144 h, referenced against ferrocene as an internal standard.

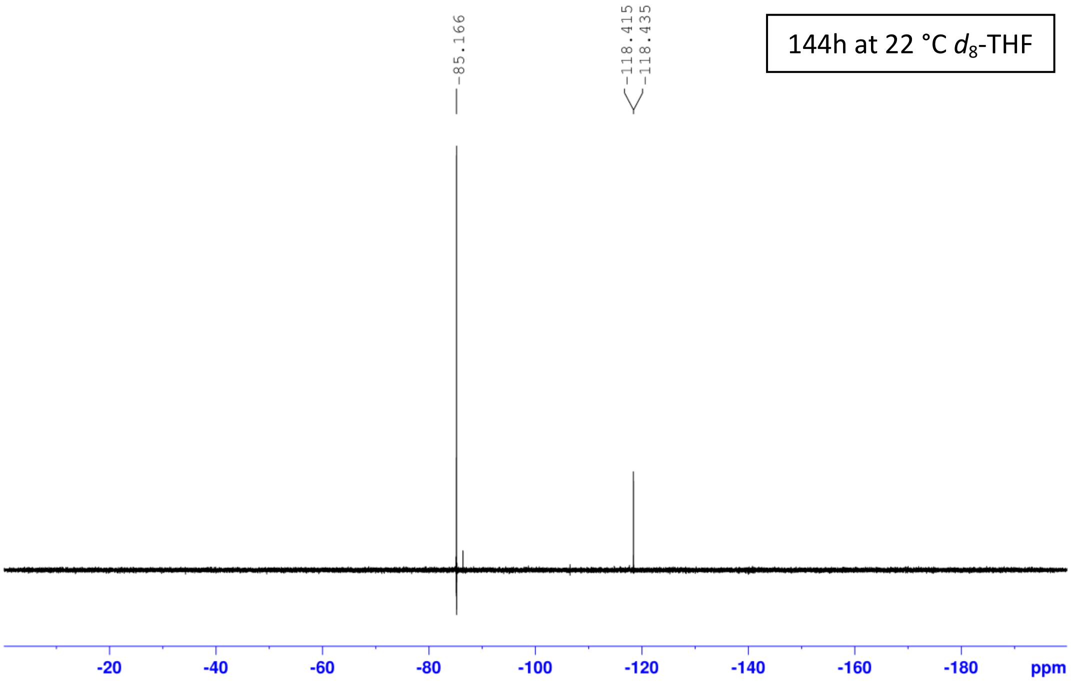

Figure S54 ^{19}F NMR spectrum showing essentially no decomposition of 2-Ga(CH_2SiMe_3)$_3$-1,3,5-F_3-C_6H_2·Li(PMDETA), **8** in d_8-THF after 144 h, referenced against ferrocene as an internal standard.

Electrophilic quenching studies:

1. Metallation of 1,3,5-trifluorobenzene by LiTMP followed by benzoyl chloride interception

An oven dried Schlenk tube was charged with LiTMP (0.5 mmol, 74 mg) and THF (10 mL) and cooled to -78 °C. 1,3,5-trifluorobenzene was added (0.5 mmol, 0.05 mL) and the bright yellow solution was stirred for 1 hour at -78 °C. Then, benzoyl chloride (0.4 mmol, 0.05 mL) was added and the solution was stirred for another hour at -78 °C. The reaction mixture was hydrolysed with water, organic products extracted with diethyl ether and the solvent was removed in vacuo. To the obtained crude mixture ferrocene (9 mg) was added as an internal standard and the mixture was dissolved in $CDCl_3$. The integration *versus* ferrocene revealed 20% of phenyl(2,4,6-trifluorophenyl)methanone (**10**).

It should be noted when the metallation was performed at room temperature, no product was obtained and decomposition seems to be the main outcome as no resonances in ^{19}F NMR can be detected.

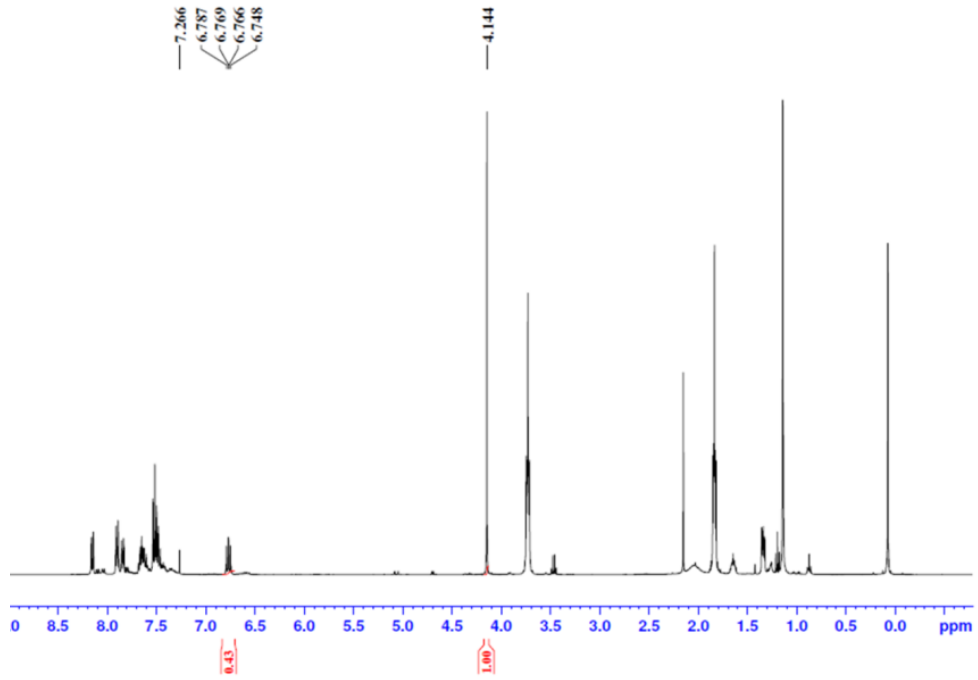

Figure S55 ^1H NMR spectrum of obtained reaction mixture in $CDCl_3$.

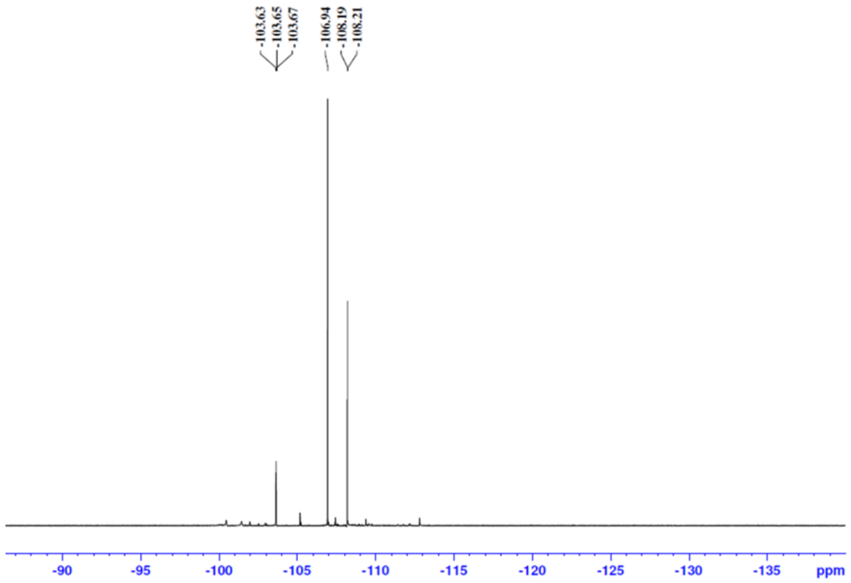

Figure S56 $^{19}F\{^{1}H\}$ NMR spectrum of obtained reaction mixture in $CDCl_3$.

2. LiTMP/iBu$_2$AlTMP metallation followed by benzoyl chloride interception

A mixture of LiTMP (74 mg, 0.5 mmol) and iBu$_2$AlTMP (141 mg, 1 mmol) in hexane 4 mL were cooled to -78 °C and 1,3,5-trifluorobenzene (0.05 ml, 0.5 mmol) was added via syringe. The reaction was stirred for 1 hour causing formation of a white precipitate. THF was added dropwise until the precipitate dissolved and all volatiles were removed in vacuo. The residue was dissolved in 10 mL of THF, to which benzoyl chloride (0.5 mmol, 0.06 mL) and Pd(PPh$_3$)$_4$ catalyst (29 mg, 0.025 mmol) were added. Obtained orange solution was stirred at room temperature for 5h, hydrolysed with water and organic products extracted with diethyl ether and the solvent removed in vacuo. To the obtained crude mixture ferrocene (9 mg) was added as an internal standard and the mixture was dissolved in $CDCl_3$. The integration *versus* ferrocene revealed 6% of phenyl(2,4,6-trifluorophenyl)methanone (**10**).

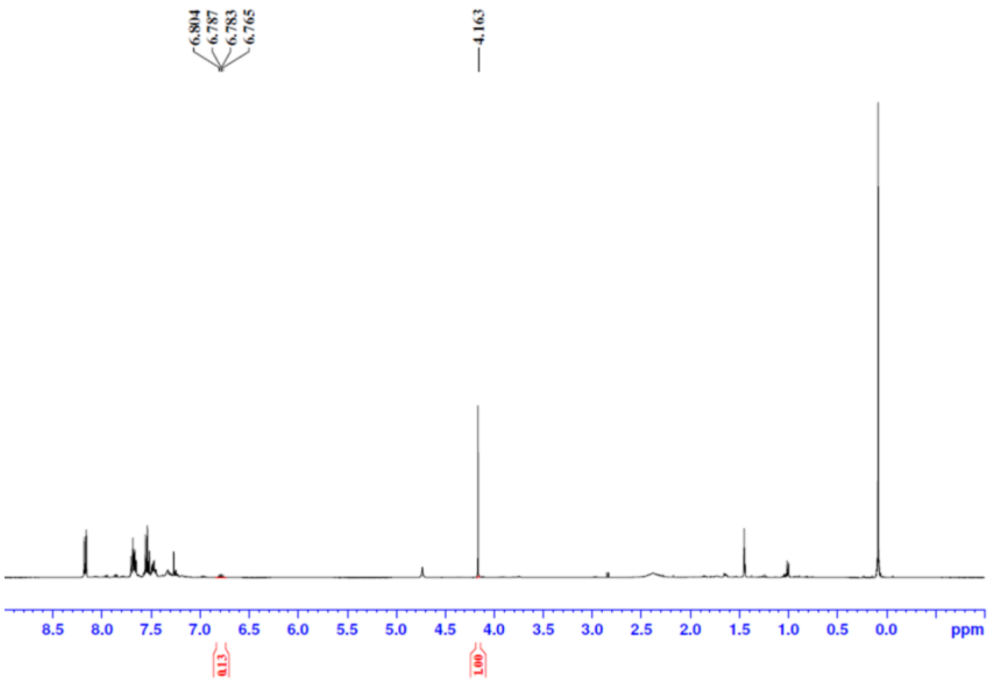

Figure S57 ^{1}H NMR spectrum of obtained reaction mixture in $CDCl_3$.

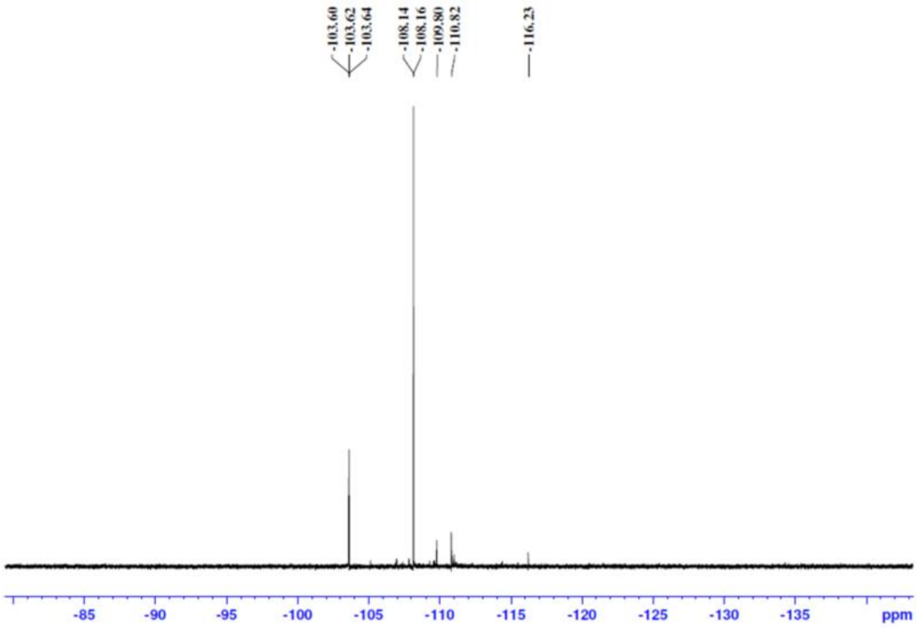

Figure S58 $^{19}F\{^{1}H\}$ NMR spectrum of obtained mixture in $CDCl_3$.

3. LiTMP/GaR_3 metallation followed by benzoyl chloride interception

An oven dried Schlenk tube was charged with 2-$Ga(CH_2SiMe_3)_3$-1,3,5-F_3-C_6H_2·LiPMDETA (161 mg, 0.25 mmol) and THF (5 mL). To this solution benzoyl chloride (0.03 mL, 0.25 mmol) was added followed by $Pd(PPh_3)_4$ catalyst (14 mg, 0.012 mmol). Bright yellow solution was refluxed at 70 °C for

4h. The reaction mixture was left to cool down to room temperature, hydrolysed with water and product extracted with diethyl ether and the solvent removed in vacuo. To the obtained crude mixture ferrocene (10 mg) was added as an internal standard and the mixture was dissolved in $CDCl_3$. The integration *versus* ferrocene revealed 79% of phenyl(2,4,6-trifluorophenyl)methanone (**10**).

It should be noted that when the reaction with benzoyl chloride was performed with the in situ mixture rather than isolated gallated intermediate, the final product was obtained in 62 % yield.

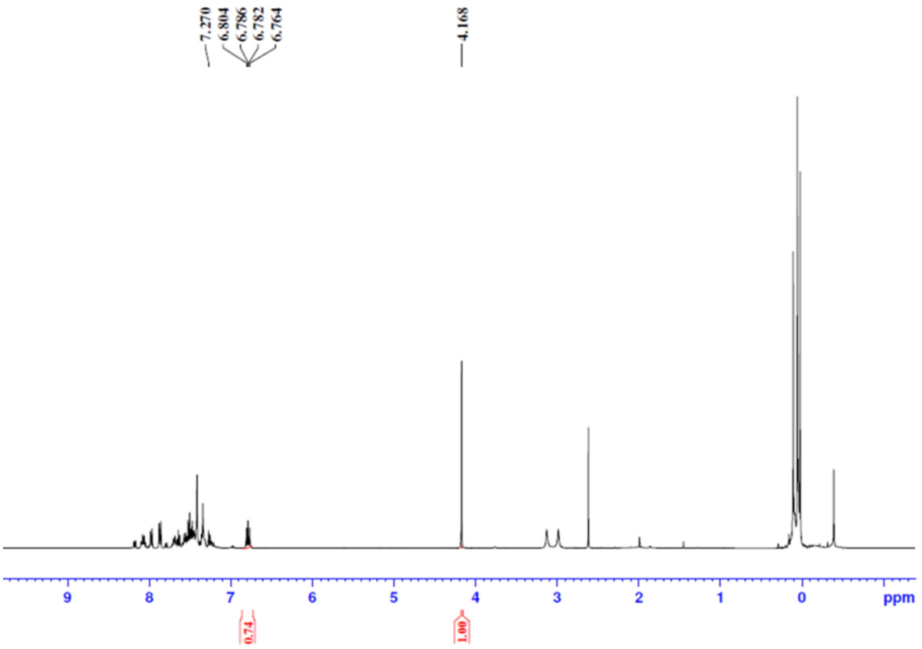

Figure S59 ^{1}H NMR spectrum of reaction mixture in $CDCl_3$.

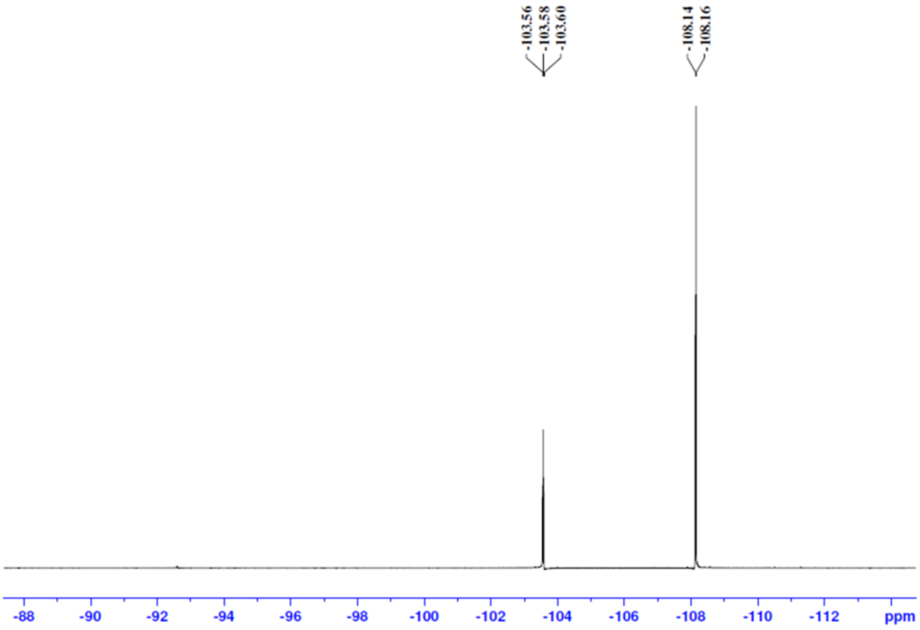

Figure S60 ^{19}F {^{1}H} NMR of reaction mixture in $CDCl_3$.

Preparation of phenyl(2,4,6-trifluorophenyl)methanone[6] (**10)**

An oven dried Schlenk tube was charged with 2-Ga(CH_2SiMe_3)$_3$-1,3,5-trifluorobenzene·LiPMDETA (0.321 g, 0.5 mmol) and THF (10 mL). To this solution benzoyl chloride (0.05 mL, 0.4 mmol) was added followed by Pd(PPh_3)$_4$ catalyst (29 mg, 0.025 mmol). Bright yellow solution was refluxed at 70 °C for 4h. The reaction mixture was left to cool down to room temperature, hydrolysed with water and product extracted with diethyl ether. The combined organic extracts were dried with $MgSO_4$ and the solvent was removed in vacuo. The crude product was subjected to flash column-cromatography (SiO_2, hexane-diethyl ether = 8:2) furnishing **10** as colourless liquid (78 mg, 66 %).

Characterisation of 10 in $CDCl_3$

^{1}H NMR (400.1 MHz, $CDCl_3$, 300 K) δ (ppm) 6.76-6.82 (m, 2H), 7.48-7.52 (m, 2H), 7.62-7.67 (m, 1H), 7.85-7.87 (m, 2H).

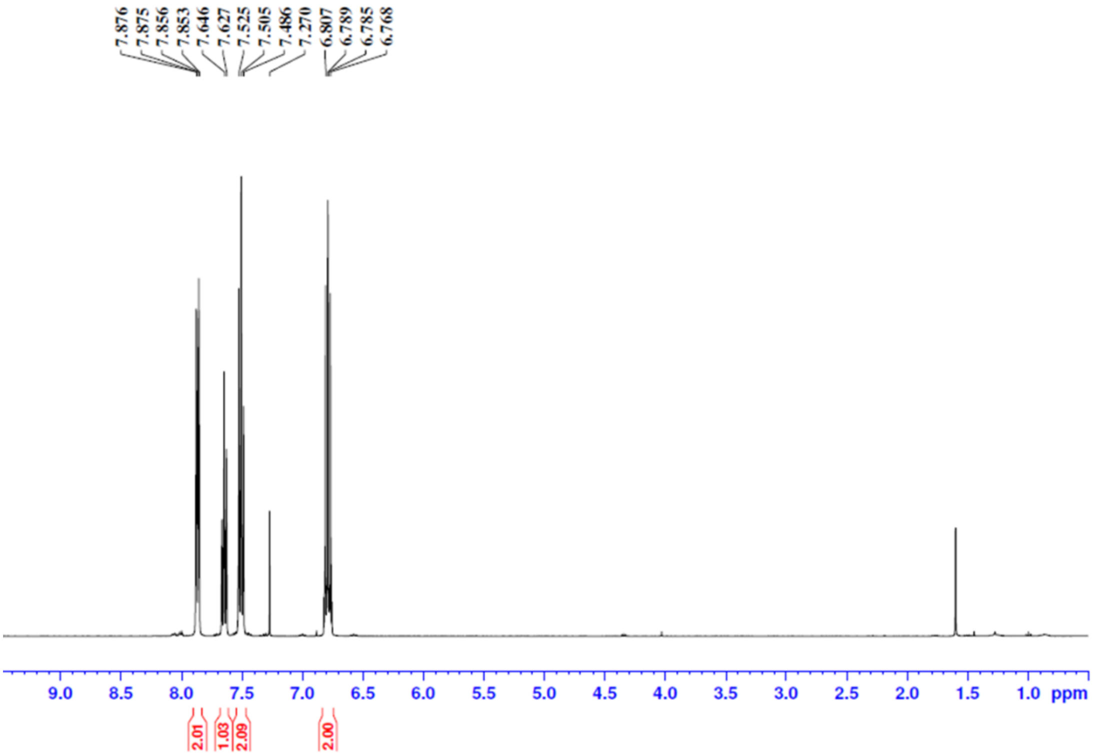

Figure S61 ^{1}H NMR spectrum of **10** in $CDCl_3$.

^{13}C NMR (100.6 MHz, $CDCl_3$, 300 K) δ (ppm) 100.9 (m), 113.7 (m), 128.8, 129.6, 134.3, 136.8, 159.1 (m), 161.6 (m), 162.4 (m), 165.0 (m), 187.8.

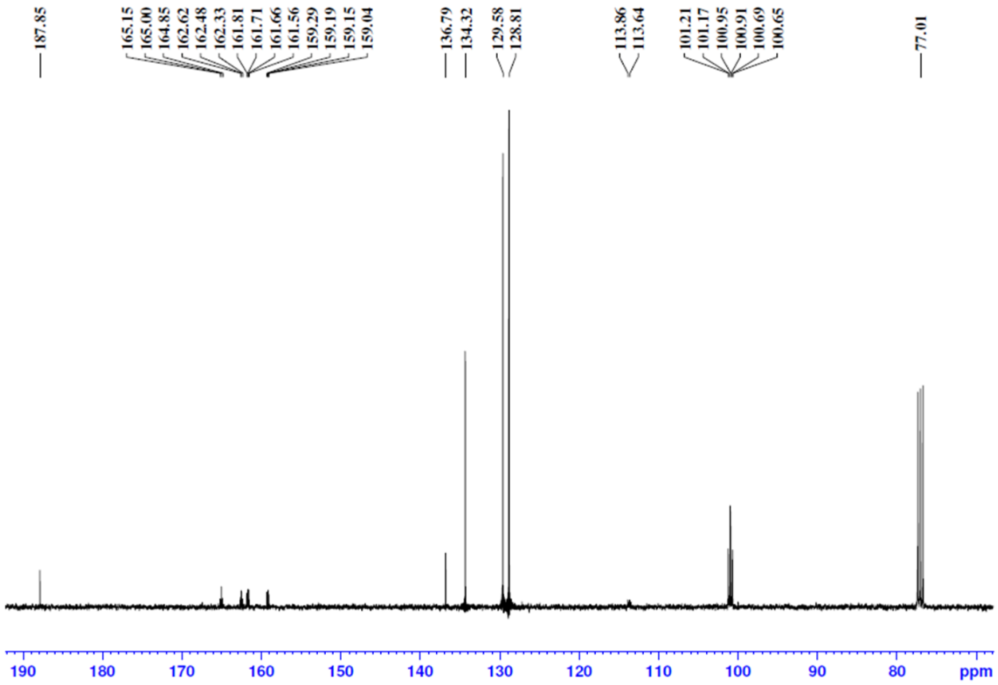

Figure S62 $^{13}C\{^1H\}$ NMR spectrum of **10** in $CDCl_3$.

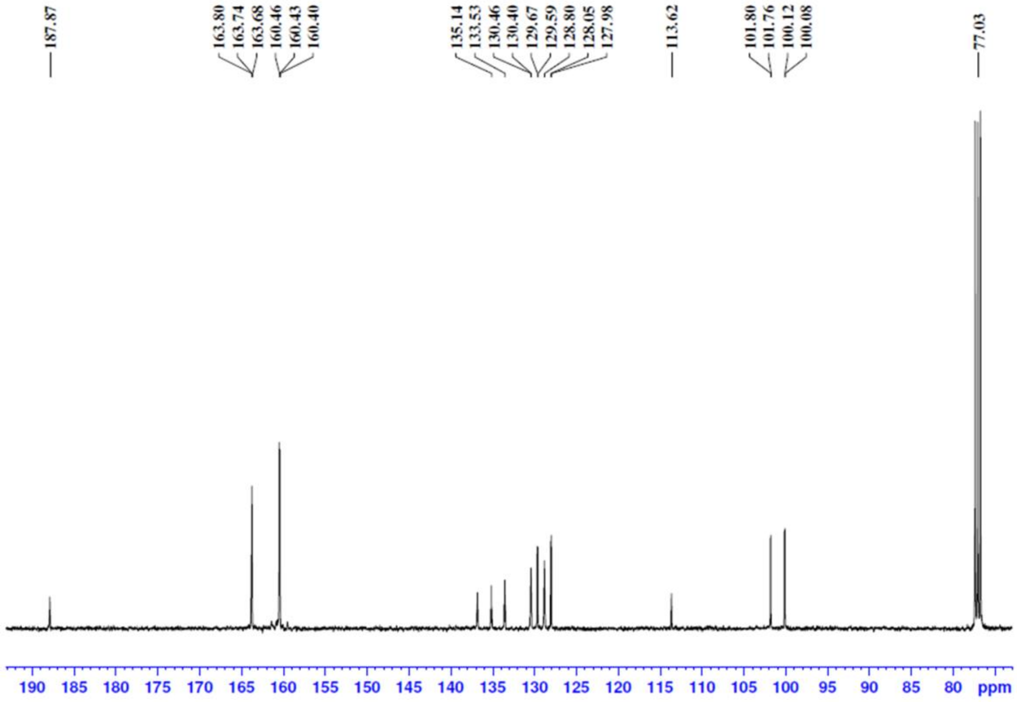

Figure S63 $^{13}C\{^{19}F\}$ NMR spectrum of **10** in $CDCl_3$.

$^{19}F\{^1H\}$ NMR (376.5 MHz, $CDCl_3$, 300 K) δ (ppm) -103.6 (t, 1F), -108.2 (d, 2F).

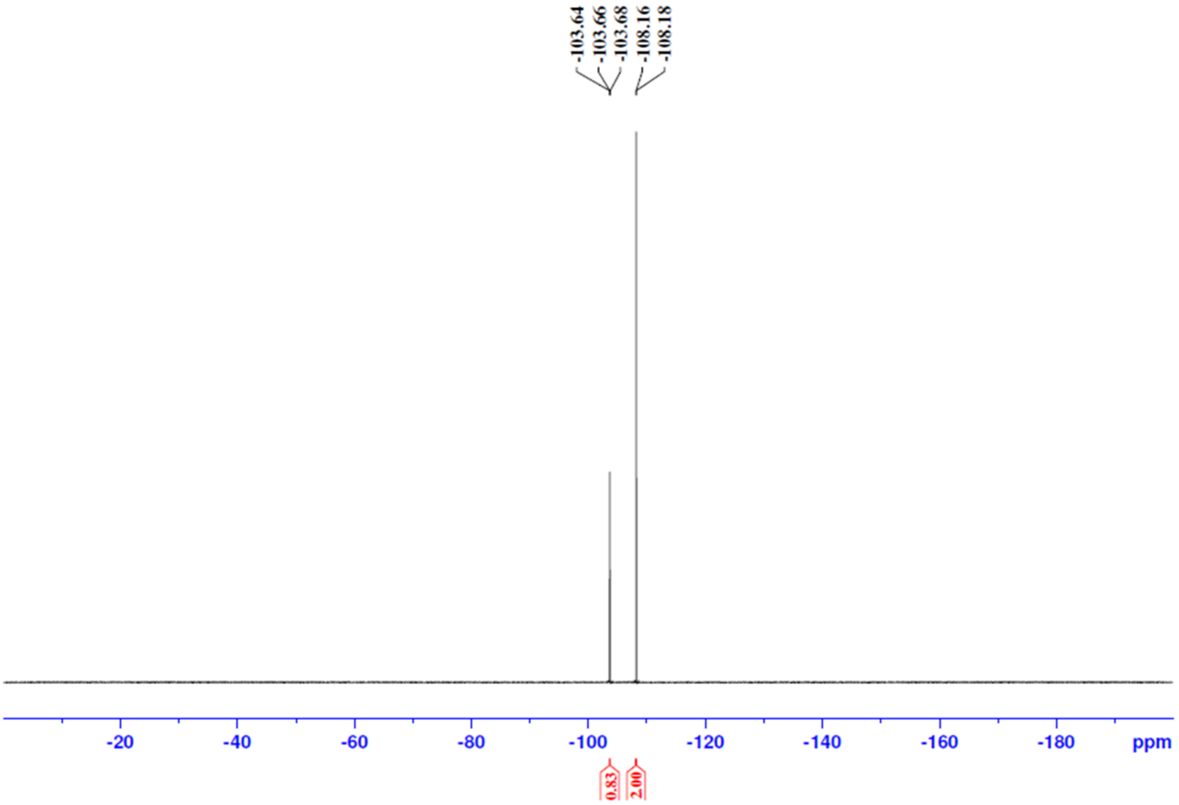

Figure S64 $^{19}F\{^1H\}$ NMR spectrum of **10** in $CDCl_3$.

1

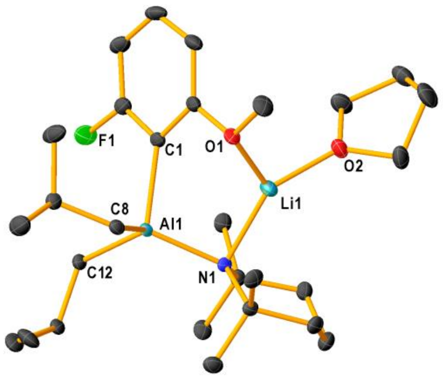

C-Al 2.0872(17) Å

2

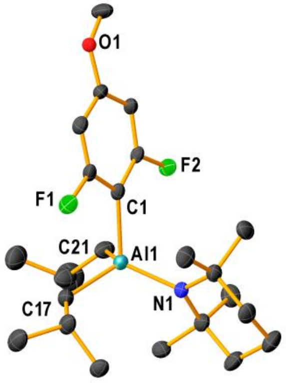

C-Al 2.076(4) Å

3

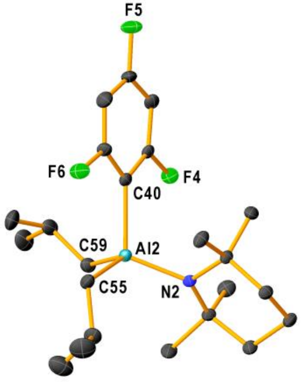

C-Al 2.090(3) Å

4

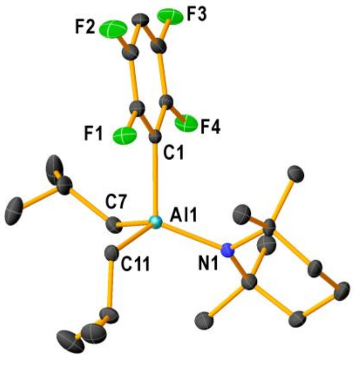

C-Al 2.106(4) Å

5

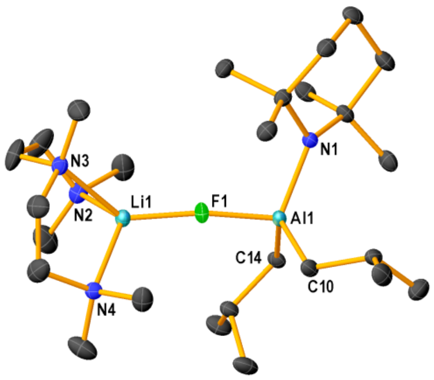

6

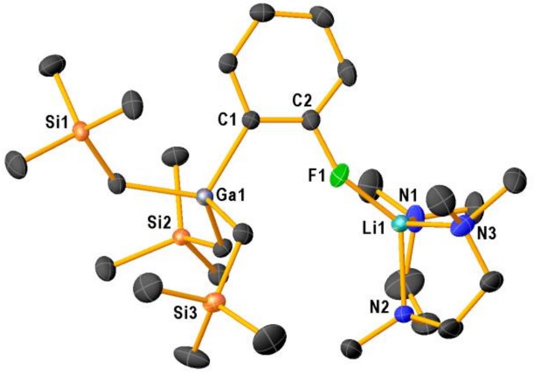

C-Ga 2.051(3) Å

8

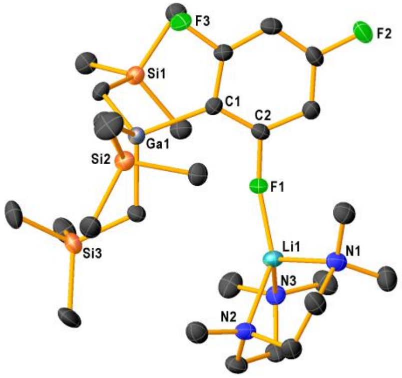

C-Ga 2.086(4) Å

9

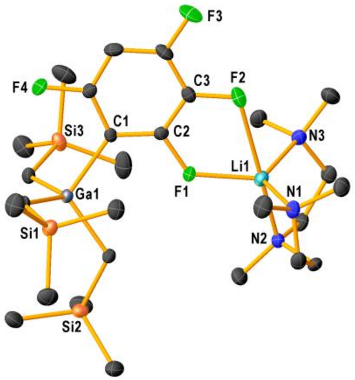

C-Ga 2.093(3) Å

Table S1 Molecular structures of 1-6, 8 and 9. 2-4 $Li(THF)_4$ cation omitted for clarity, All H-atoms omitted and thermal ellipsoids are drawn at 30% probability.

Table S2 **Crystallographic data and refinement details for complexes 1-4.**

	**1**	**2**	**3**	**4**
Empirical formula	$C_{28}H_{50}AlFLiNO_2$	$C_{41.25}H_{75.25}AlFLiNO_{5.25}$	$C_{39}H_{70}AlF_3LiNO_4$	$C_{39}H_{69}AlF_4LiNO_4$
Mol. Mass	485.61	741.19	707.88	725.87
Crystal system	monoclinic	triclinic	monoclinic	orthorhombic
a/ Å	11.5163(11)	11.7974(6)	16.4189(6)	23.5016(9)
b/ Å	14.9261(18)	18.2311(8)	15.7091(5)	10.7939(5)
c/ Å	17.6578(16)	21.2432(9)	32.7030(13)	16.4745(7)
α/ °	90	85.469(4)	90	90
β/ °	107.189(10)	89.119(4)	97.178(4)	90
γ/ °	90	76.569(4)	90	90
V/ Å^3	2899.2(5)	4430.1(4)	8368.8(5)	4179.2(3)
Z	4	4	8	4
λ/ Å	0.71073	0.71073	0.71073	0.71073
Measured reflections	14919	33556	37539	22749
Unique reflections	7062	15550	16410	9917
R_{int}	0.0329	0.0457	0.0573	0.0352
Observed rflns [I>2σ(I)]	5172	7983	9311	7411
GooF	1.050	1.015	1.033	1.082
R [on *F*, obs rflns only]	0.0589	0.0883	0.0721	0.0703
ωR [on F^2, all data]	0.1278	0.2571	0.1623	0.1637
Largest diff. Peak/hole. e/ Å^{-3}	0.333 / -0.189	0.721 / -0.397	0.528 / -0.302	0.633 / -0.239

Table S1 (continued) Crystallographic data and refinement details for complexes 5, 6, 8 and 9.

	**5**	**6**	**8**	**9**
Empirical formula	$C_{26}H_{59}AlFLiN_4$	$C_{27}H_{60}FGaLiN_3Si_3$	$C_{27}H_{58}F_3GaLiN_3Si_3$	$C_{27}H_{57}F_4GaLiN_3Si_3$
Mol. Mass	480.69	606.71	642.69	660.68
Crystal system	monoclinic	monoclinic	monoclinic	triclinic
a/ Å	9.1995(4)	11.9509(4)	18.5440(6)	11.4844(5)
b/ Å	17.1972(7)	16.7425(4)	11.3242(3)	12.5989(5)
c/ Å	19.5257(8)	18.6257(6)	18.0613(5)	13.1501(5)
α/ °	90	90	90	88.047(3)
β/ °	94.159(4)	98.175(3)	103.122(3)	85.539(3)
γ/ °	90	90	90	85.393(3)
V/ $Å^3$	3080.9(2)	3688.9(2)	3693.77(19)	1890.06(13)
Z	4	4	4	2
λ/ Å	0.71073	0.71073	1.54184	0.71073
Measured reflections	15627	20035	32121	17592
Unique reflections	7484	9137	7327	8535
R_{int}	0.0308	0.0348	0.0422	0.0619
Observed rflns [I>2σ(I)]	5155	6342	6467	5957
GooF	1.015	1.026	1.042	1.051
R [on *F*, obs rflns only]	0.0689	0.0573	0.0428	0.0548
ωR [on F^2, all data]	0.1789	0.1352	0.1207	0.1187
Largest diff. Peak/hole. e/ $Å^{-3}$	0.796 / -0.398	1.079 / -0.804	0.654 / -0.362	0.597 / -0.600

References

1. E. Hevia, A. R. Kennedy, R. E. Mulvey, D. L. Ramsay and S. D. Robertson, *Chem*. *Eur*, *J*., 2013, **19**, 14069-14075.

2. R. E. Mulvey, D. R. Armstrong, B. Conway, E. Crosbie, A. R. Kennedy and S. D. Robertson, *Inorg*. *Chem*., 2011, **50**, 12241-12251.

3. L. M. Dennis and W. Patnode, *J. Am*. *Chem*. *Soc*., 1932, **54**, 182-188.

4. G. M. Sheldrick, Acta Crystallogr. **2007**, A64, 112-122.

5. O. V. Dolomanov; L. J. Bourhis; R. J. Gildea; J. A. K. Howard; H. Puschmann, J. Appl. Cryst. **2009**, 42, 339-341.

6. The ^{1}H NMR spectrum of phenyl (2,4,6-trifluorophenyl)methanone is in agreement with the one previously reported in T. J. Luker, R. T. Mohammed, S. Thom, A. Patel, PCT Int. Appl. **2007**, WO2007039736 A1 20070412. Note that this ^{1}H NMR was the only piece of characterisation of this compound.
